# Efficient Hamiltonian, structure and trace distance learning of Gaussian states

**DOI:** 10.1038/s41467-026-77405-3

**Published:** 2026-09-16

**Authors:** Marco Fanizza, Cambyse Rouzé, Daniel Stilck França

**Affiliations:** 1 Inria, Télécom Paris - LTCI, Institut Polytechnique de Paris, Palaiseau, France; 2 Department of Mathematical Sciences, University of Copenhagen, Copenhagen, Denmark; 3 Física Teòrica: Informació i Fenòmens Quàntics, Departament de Física, Universitat Autònoma de Barcelona, Bellaterra (Barcelona), Spain; 4 Univ Lyon, ENS Lyon, UCBL, CNRS, Inria, LIP, Lyon, France

**Keywords:** Quantum information, Computer science

## Abstract

When learning descriptions of many-body quantum systems, assuming locality of interactions can dramatically reduce the required number of observations and computational time compared to unstructured approaches. In this work, we uncover an infinite-dimensional instance of this phenomenon, initiating the study of Hamiltonian learning for positive-temperature bosonic Gaussian states. We present sample- and computationally efficient protocols for inferring the parameters and graph structure of underlying quadratic Hamiltonians, provided that temperature, squeezing, displacement, and the interaction graph’s maximal degree are bounded. Notably, our protocol relies solely on heterodyne measurements, which are often experimentally feasible, and achieves a sample complexity that scales only logarithmically with the number of modes. Furthermore, our techniques allow for the learning of positive-temperature Gaussian states in trace distance with quadratic scaling in precision and polynomial scaling in the number of modes.

## Abstract (web summary)

This study shows how accessible measurements can efficiently reveal the interaction strengths and connectivity of positive-temperature bosonic Gaussian systems with local interactions, with observations growing only logarithmically with system size.

## Introduction

Inferring parameters of quantum systems from measurement data is a fundamental task in quantum information science that has seen significant advancements in recent years^1–15^. Although it has been known for roughly a decade that complete tomography of general quantum states inevitably requires a sample complexity scaling exponentially in the system’s size^16–18^, recent works have shown that it is possible to obtain a lot of information of practical interest much more efficiently. This is exemplified by the framework of classical shadows^19^. Another fruitful approach to obtain efficient algorithms is to consider the learning of more restricted and physically motivated quantum states and evolutions, such as those generated by local Hamiltonians^6,8–11^ or their Gibbs states^1–3,5,7,12^, where the problem of inferring the parameters of the underlying Hamiltonian is broadly referred to as the Hamiltonian learning problem. This line of work culminated in sample- and computationally efficient algorithms under various assumptions. A sample-efficient algorithm for an arbitrary constant-temperature local quantum Hamiltonian was given by ref. ^2^; however, the sample complexity was still polynomial in system size and the postprocessing was only efficient under further assumptions on the model, as it required solving a maximum entropy optimization problem. This was followed by work of ref. ^1^, which focused on the high-temperature regime. Resorting to cluster expansion techniques, they were able to obtain optimal sample complexity bounds and efficient post-processing. More recently, ref. ^3^ gave Hamiltonian learning algorithms for general Hamiltonians with efficient sample and computational complexity based on sums-of-squares relaxations.

This program can be understood as a quantum version of the widely studied classical problem of learning a discrete graphical or Ising model^20–26^. For classical discrete graphical models it is known how to learn the graph with a number of samples that scales logarithmically in the system’s size^22–24^, and known algorithms are essentially optimal^20^. Substantial progress is still required to put the quantum protocols on an equal footing with their classical counterparts. At the time the first version of the present work was made publicly available, the best known general-purpose computationally efficient quantum protocols for Hamiltonian learning from a Gibbs state of spin systems required previous knowledge of the graph with respect to which the Hamiltonian is local and had a sample complexity that is polynomial in the system’s size^3,12^. Afterward, logarithmic sample complexity was proven by ref. ^27^. Furthermore, the computationally efficient quantum algorithms have a much worse scaling in precision^3,12,27^ compared to the classical case and, in a nutshell, the main technical barrier for quantum algorithms is the lack of (exact) conditional independence structures for the underlying states, which is central to most classical results.

**Fig. 1:**
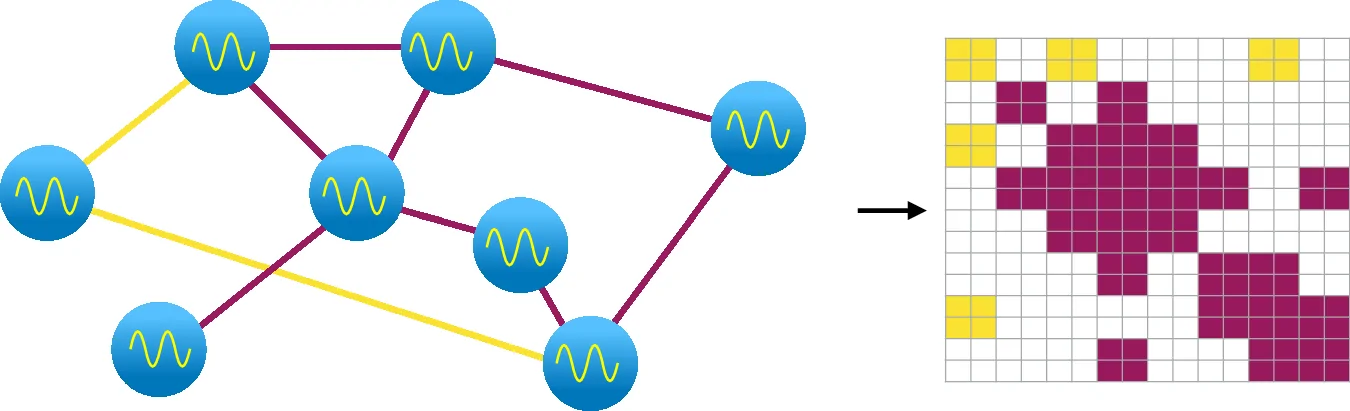
Graph of interactions for 8 modes and corresponding structure of the Hamiltonian matrix. Vertices are numbered from left to right. The submatrices corresponding to the neighborhood of vertex 1 at distance 1, i.e., {1, 3, 7}, are highlighted in yellow. The degree of this graph is 4.

In the classical case, there is also an extensive literature on learning Gaussian graphical models^28–30^, which can be understood as continuous-variable versions of discrete graphical models. In the last years, many fundamental questions related to this problem were settled, such as finding a sample-optimal algorithm for graph recovery in the bounded-degree regime^31^ matching information-theoretic bounds^32^. Related work has also studied Gaussian learning under differential privacy^33^ and estimation of Gaussian mixtures^34^. The natural quantum variation of learning a Gaussian model is that of learning bosonic Gaussian states^35^, which correspond to Gibbs and ground states of quadratic Hamiltonians. Their use as a learning resource is physically motivated by the equilibration of open quantum systems^36–39^. Bosonic Hamiltonians with a finite number of modes are used to describe a multitude of interacting many-body quantum systems, such as cold atoms in optical lattices, magnetization fields, optomechanical systems, trapped ions, and electromagnetic fields in cavities, interacting with matter^40^. In many cases of interest, the interaction strength decays with the distance with respect to a graph. Sparse Hamiltonians considered in this work are models where the interactions are assumed to be zero if vertices are not connected, for example, because the corresponding sites are further than some fixed distance on a lattice. This includes the approximation of considering non-zero interactions only between first neighbors. In Fig. 1, we show an example of an interaction graph and its corresponding structure of the Hamiltonian matrix. Experimentally realizing local (and thus sparse) Hamiltonians, including bosonic ones, is the goal of analog quantum simulation^41^, both for reproducing models of condensed matter physics and for creating and studying meta-materials that cannot be found in nature^42–44^. Current programmable simulators can moreover implement Hamiltonians with engineered graph structures in a variety of platforms, see Supplementary Note 2 for a short discussion. Here, we mention that the closest work to our idealized setting is ref. ^45^, which reports the engineering of various interaction graphs, including rings, chains, ladders, non-Archimedean geometries, in an array of atomic ensembles with 18 sites and 10^4^ Rubidium atoms per site, interacting via spin-exchange. The collective magnetizations at each site can be treated as bosonic variables in the relevant regime, with a quadratic Hamiltonian. The paper also shows procedures to verify the interactions from two-point correlation functions of the magnetization, using certain approximations and assumptions. Other notable examples of platforms with programmable bosonic Hamiltonians are refs. ^46–48^. In all the above, quadratic hopping Hamiltonians already explore non-trivial physics and testing their correct implementation is paramount to go forward to the simulation of interacting models. The problem of Gaussian Hamiltonian learning can also be seen as a non-asymptotic instance of Gaussian multiparameter and many-body sensing^49–52^.

Somewhat surprisingly, given the prevalence and importance of bosonic Gaussian Hamiltonians, to the best of our knowledge, previous works that considered bosonic Hamiltonian learning problems focused on learning parameters from dynamics^6,9^, not Gibbs states. In our work, we address this question by studying three related tasks for bosonic Gaussian states: Hamiltonian learning, graph learning and trace distance learning. We show that it is possible to learn all the parameters of a quadratic Hamiltonian on *m* modes on a known interaction graph of bounded degree with a number of samples scaling logarithmically in *m*, the natural measure of the size of the system, with nearly inverse-quadratic dependence on the target error, when all the other parameters are bounded. Furthermore, the classical postprocessing of the data is efficient and we resort only to heterodyne measurements, which are often simple to implement in the lab. We also show how to recover an unknown interaction graph, assuming bounded parameters and a lower bound on every nonzero interaction. The corresponding sample complexity is logarithmic in *m* and nearly inverse-quadratic in the interaction threshold parameter. Thus, our method only requires experimentally feasible measurements, is highly scalable, and it provides quantitative guarantees on the precise estimation of the interactions and the graph in bosonic simulators that hold at any positive temperature and that are not model-specific. In the context of sensing, our results imply that without trying to achieve the optimal information-theoretic limits, which are highly sensitive to the precise values of the parameters themselves, one can ensure that the estimate of any parameter encoded linearly in the Hamiltonian matrix is robust with respect to uncertainties in the other parameters. In addition, we obtain a protocol to learn a positive-temperature Gaussian state in trace distance with an inverse-quadratic scaling in error. Previous work^53,54^ obtained polynomial dependence on the number of modes but inverse-quartic scaling in error, while imposing fewer assumptions on the state compared to our result. Closely contemporaneous work also obtained an inverse-quadratic scaling by a different method that also applies to pure states, and a different concrete trace distance bound^55^.

**Fig. 2:**
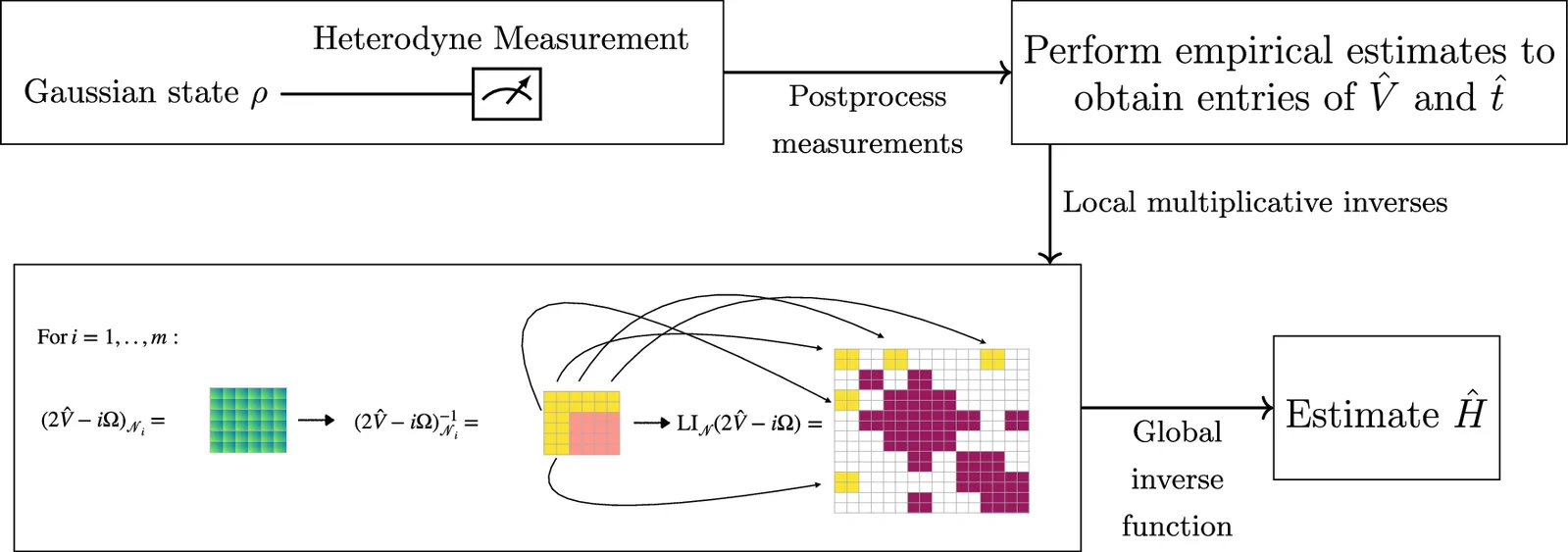
Schematic workflow for our Hamiltonian learning protocol for a known graph. We first perform heterodyne measurements on copies of a Gaussian state *ρ*. We then use the outcomes to obtain empirical estimates of the entries of the covariance matrix and the mean. Importantly, for most of our results, we will only require that the precision with which we estimate each entry does not scale with the number of modes in the system. The reason for that is the next step, our local inversion procedure. Here, we are given a neighborhood structure ${\{{{{\mathcal{N}}}}_{i}\}}_{i\in [m]}$. Roughly speaking, for each *i*, ${{{\mathcal{N}}}}_{i}$ contains all the vertices that are at most a distance *l* from *i*, where *l* depends on the target precision. We then only invert the matrix $(2\hat{V}-i\Omega )$ on the submatrix corresponding to each neighborhood ${{{\mathcal{N}}}}_{i}$ (in the figure, we illustrate this step for the neighborhood of vertex 1 of Fig. 1, for *l* = 1) and “stitch together” the global matrix combining all estimates from the various neighborhoods. We then apply the function that computes *H* from (2*V*−*i*Ω)^−1^ to the resulting matrix to form our guess of the Hamiltonian.

From a technical perspective, our main innovations are various perturbation bounds for covariance and Hamiltonian matrices of Gaussian states that are of independent interest, combined with what we call the local inversion subroutine, a key part of our algorithms for graphical models. This allows us to estimate the Hamiltonian of Gaussian states by accessing only small blocks of the covariance matrix at a time, with the precision of the estimates only scaling with the size of the blocks and not the whole system. The workflow of the Hamiltonian learning algorithm is represented in Fig. 2.

## Results

### Notations and background

Let us review basic concepts of continuous variable systems and explain the connection between Gaussian quantum states and classical Gaussian graphical models. We refer to ref. ^35^ for a more complete treatment and proofs of the statements here. A continuous variable (CV) system with *m* modes is defined on the Hilbert space ${{{\mathcal{H}}}}_{m}:={L}^{2}({{\mathbb{R}}}^{m})$, and we denote the canonical operators on each mode as *X*_*j*_, *P*_*j*_ (*j* = 1, …, *m*). Define the formal vector

$$R:={\left({X}_{1},{P}_{1},\ldots,{X}_{m},{P}_{m}\right)}^{\top }$$

and the symplectic form

$$\Omega :={\bigoplus }_{i=1}^{m}\left(\begin{array}{cc}0&1\\ -1&0\end{array}\right)$$

(this is a block diagonal matrix, where all diagonal blocks are 2 × 2 matrices), in terms of which the canonical commutation relations read (at least when evaluated on Schwartz functions)

1 $$[{R}_{j},{R}_{k}]=i{\Omega }_{jk}\,.$$

With a slight abuse of notation, we will often denote by the same symbol Ω the symplectic form for different sets of modes. The quantum covariance matrix *V* and mean vector *t* of a state *ρ* are defined by

2 $${t}_{j}:=\,{{\mbox{tr}}}\,\left[\rho {R}_{j}\right]\,,\quad {V}_{jk}:=\frac{1}{2}\,{{\mbox{tr}}}\,\left[\rho \left\{{R}_{j}-{t}_{j},\,{R}_{k}-{t}_{k}\right\}\right]\,,$$

provided that these expressions are well defined, and it holds that $V\ge \pm \frac{i}{2}\Omega$, where *A* ≥ 0 means that *A* is positive semi-definite.

Gaussian states are those of the form [ref. ^35^, Theorem 12.23]

3 $$\rho (t,H)=\,\frac{{e}^{-{(R-t)}^{\top }H(R-t)}}{\,{\mbox{tr}}\,[{e}^{-{(R-t)}^{\top }H(R-t)}]}\,,$$

or limits thereof, where the matrix *H* is real symmetric and positive-definite, it is referred to as the (Gaussian) Hamiltonian of the system and satisfies

4 $$2i\Omega V=\,{\mbox{coth}}\,(iH\Omega )\,.$$

Inverting this relation, we have

5 $$H=\frac{1}{2}\ln \left(\frac{2i\Omega V+I}{2i\Omega V-I}\right)i\Omega=\frac{1}{2}\ln \left(I+\frac{2}{2i\Omega V-I}\right)i\Omega .$$

A key tool in our analysis is to express the above via an integral representation of the matrix logarithm. In particular, by writing $K=\frac{2}{2V-i\Omega }$, we have

6 $$H=f(K):=\frac{1}{2}\,Ki\Omega \int_{0}^{\infty }\frac{1}{I+\frac{t}{t+1}Ki\Omega }\frac{dt}{{(t+1)}^{2}}i\Omega \,.$$

A fundamental property of *H* that we will repeatedly use is the following. For any Hamiltonian *H*, let a symplectic diagonalization be

7 $$H={S}^{-\top }D{S}^{-1}\,,$$

with $D={\oplus }_{i=1}^{m}{d}_{i}{I}_{2}$, with *d*_*i*_ > 0, and $S\in {{\rm{Sp}}}(2m,{\mathbb{R}})$. In the following, $\parallel S\parallel,{d}_{\min },{d}_{\max }$ will be used as parameters, as they have a natural physical interpretation. See Section “Parametrization” for a discussion on this choice of parameters and their relation with the norm and the condition number of *H*.

Finally, the locality structure will be also fundamental in what follows. We will say that a Hamiltonian matrix *H* has graph $G=([m],{\mathsf{E}})$ made of *m* vertices, where two distinct vertices *i*, *j* ∈ [*m*] are connected by an edge whenever the 2 × 2 sub-matrix ${\left\{{H}_{2i-\delta,2j-{\delta }^{{\prime} }}\right\}}_{\delta,{\delta }^{{\prime} }\in \{0,1\}}$ is non-zero.

#### Relation to Gaussian graphical models

Let us briefly recall the extensively studied problem of learning Gaussian graphical models, initiated by refs. ^28,29^, and make the connection to the quantum problems we will study. In the problem of estimating a Gaussian graphical model, one is given *n* i.i.d. ${X}_{1},\ldots,{X}_{n}\in {{\mathbb{R}}}^{m}$ samples from a multivariate normal distribution ${{\mathcal{N}}}(t,\Sigma )$ with mean vector $t\in {{\mathbb{R}}}^{m}$ and covariance matrix $\Sigma \in {{\mathbb{R}}}^{m\times m}$. It is then known that the inverse of the covariance matrix *Θ* = *Σ*^−1^, often also called the precision matrix, encodes the conditional independence structure of the random variables. Furthermore, the pdf of the random variables is given by:

8 $${f}_{t,\Sigma }(x)=\frac{1}{{(2\pi )}^{m/2}\sqrt{\det (\Sigma )}}\exp \left(-\frac{1}{2}{(x-t)}^{\top }{\Sigma }^{-1}(x-t)\right)$$

This is in direct analogy with Eq. (3), where we see that the quantum state can be expressed in a similar way, albeit with a different functional dependence on the covariance matrix for the matrix in the exponent. But the interpretation in the quantum case of the nonzero entries of *H* is similar to the classical case, as these represent the systems that “interact” with each other.

In the literature on learning of Gaussian graphical models, one is often interested in learning the conditional independence structure of the random variables, which corresponds to estimating the sparsity structure of *Θ*. From that, one is also usually interested in estimating the parameters of *Θ*, while having a scaling of the sample complexity that only scales logarithmically with the dimension of the random variables. Thus, we see that the problem of learning the sparsity structure of *H* is in direct correspondence with that of learning the conditional independence structure graph of a Gaussian graphical model. And that of estimating the entries of *H* in turn corresponds to that of estimating the parameters of the Gaussian graphical model.

Furthermore, the two problems also share some technical aspects; for instance, in contrast to the discrete case, the underlying objects we sample from typically take unbounded values and a finer understanding of concentration is required. But the quantum problem poses unique challenges that are not present in the classical case. One difference is that the uncertainty principle obstructs from sampling from a multivariate normal with covariance matrix *V* measuring one copy of the state: we resort to heterodyne measurement, whose output is a multivariate normal with covariance matrix *V* + *I*/2. More importantly, the more complicated functional dependence between the covariance matrix and *H* makes the analysis more challenging, since entries of *V* corresponding to the neighborhood of a vertex *i* are not sufficient by themselves to reconstruct exactly the interactions of *i* with its neighbors (matrix elements *H*_*ij*_). This fact can be understood as related to the lack of conditional independence in the quantum case: in general, one cannot reconstruct the state of the system by applying a channel on the complement of vertex *i* that acts nontrivially only on the vertices connected to *i*. Our local inversion method, instead, goes further in the graph to account for the lack of conditional independence.

The problem of estimating Gaussian graphical models with sparsity is a central theme of high-dimensional statistics, and we refer to books such as refs. ^56–58^ for a more in-depth history of the problem and we provide a more detailed review of technical results in the classical literature in Supplementary Note 2. Here we mention that, when the graph is known, a simple procedure using local inversions for each neighborhood suffices to find the coefficients^56^ with a number of copies scaling as ${{\mathcal{O}}}(\frac{\log m}{{\epsilon }^{2}})$ under bounded degree and ∥*Θ*∥_*∞*_, with a matching lower bound^32^. In fact, most of the literature has directly dealt with the harder problem of estimating the precision matrix when the graph is not known, as well as estimating the graph itself. A variety of efficient and practical algorithms is available^30,59–65^ having computation complexity polynomial in the number of variables. The sample complexity is ${{\mathcal{O}}}(\log m)$, but only if appropriate assumptions, that can be broadly linked to the condition number of *Θ* being bounded, hold^31^. For the problem of graph selection, ref. ^31^ showed a sample complexity ${{\mathcal{O}}}(\frac{\Delta \log m}{{\kappa }^{2}})$, with *κ* being a lower bound on the relative strengths of the interactions, and no condition number dependence, and matching the *κ* and *m* dependence in the lower bounds in ref. ^32^. The tradeoff is a worse scaling in computational complexity, still polynomial in *m* but with a degree depending on the degree of the graph, since for each node one needs to test all possible neighborhoods (improvements for particular model classes are possible^66^). Our graph learning algorithm is inspired by the approach in ref. ^31^ (especially their DICE algorithm). However, we were not able to remove the condition number dependence and leave it to future work.

### Main theorems

In this paper, we make substantial progress on three fundamental problems related to learning Gaussian quantum states: learning in trace distance, Hamiltonian learning and graph learning. In our derivations and results, we use a suitable parametrization to control the complexity of the Gaussian states assumed as input to the algorithm. In particular, we consider the amount of squeezing in the Gaussian state (namely the operator norm of the symplectic diagonalization matrix *S*), the “temperature” of the state (as parametrised  by the smallest and largest symplectic eigenvalues ${d}_{\min },{d}_{\max }$ of *H  *), the maximal displacement ${t}_{\max }$, the number of modes *m* and the maximal degree of the graph of the Hamiltonian, equal to Δ−1. For all our results, we will assume we have constant-size bounds on the relevant parameters. In particular, in the Hamiltonian and graph learning results stated in the main text we will adopt a *bounded parameters condition* stating that Δ, ∥*S*∥_*∞*_, ${d}_{\max },{d}_{\min }^{-1},{t}_{\max }:={{\max }_{i}}| {t}_{i}|$ are all smaller than some known constant. More detailed results are available in the Supplementary Information. See Table 1 and the “Methods” for a recap of notation, interpretations and relations between the parameters. We summarize our results and the comparison with previous and concurrent work in Table 2.

**Table 1:** Notation and relations between the several parameters used in the analysis

Symbol	Description	Useful relations
*m*	Number of modes	–
*H*	Hamiltonian matrix	–
Δ	Degree of the interaction graph +1	$\parallel H{\parallel }_{\infty }\le 2\Delta {{\max }_{i,j}}| {H}_{i,j}|$
${\mathsf{E}}$	Edges of the interaction graph	$0 < {{\max }_{{\delta }_{1},{\delta }_{2}\in \{0,1\}}}| {H}_{2i-{\delta }_{1},2j-{\delta }_{2}}|$ iff $(i,j)\in {\mathsf{E}}$
*κ*	Lower bound on absolute values of Hamiltonian interaction matrix elements	$\kappa < {{\max }_{{\delta }_{1},{\delta }_{2}\in \{0,1\}}}| {H}_{2i-{\delta }_{1},2j-{\delta }_{2}}|$ for any $(i,j)\in {\mathsf{E}}$.
*D*, *S*	Williamson’s normal form of *H*	*H* = *S*^−⊤^*DS*^−1^
*V*	Covariance matrix	$V=\frac{1}{2}S\coth (D){S}^{\top }$
*t*	Vector of first moments	–
*E*	Expectation value of the total energy	$\,{\mbox{tr}}\,[V]+\parallel t{\parallel }_{2}^{2}=2E$
${d}_{\max }$	Maximum symplectic eigenvalue of *H*, maximum inverse temperature of a normal mode, closeness to non-full-rank states	$\parallel D{\parallel }_{\infty }={d}_{\max }\le \parallel H{\parallel }_{\infty }$
${d}_{\min }$	Minimum symplectic eigenvalue of *H*, minimum inverse temperature of a normal mode, spectral gap of *R*^⊤^*HR*	$$\begin{array}{l}\parallel {D}^{-1}{\parallel }_{\infty }={d}_{\min }^{-1}\le \parallel {H}^{-1}{\parallel }_{\infty }\\ {(1-{e}^{-2{d}_{\min }})}^{-1}\le 8{E}^{2}\end{array}$$
∥*S*∥_*∞*_	Operator norm of *S*, maximum amount of squeezing	$$\begin{array}{rcl}1\le \parallel S{\parallel }_{\infty }^{2}&\le &\frac{\parallel H{\parallel }_{\infty }}{{d}_{\min }}\\ \parallel S{\parallel }_{\infty }&=&\parallel {S}^{-1}{\parallel }_{\infty }\\ \parallel S{\parallel }_{\infty }^{2}&\le &4E\end{array}$$

**Table 2:** Summary of results and comparison with relevant work

Work	Task	Assumptions	Measurements	Sample complexity
^53^	Trace distance	–	Homodyne	${{\mathcal{O}}}(\frac{{m}^{7}{E}^{3}}{{\epsilon }^{4}})$
^55^	Trace distance	–	Heterodyne	${{\mathcal{O}}}\left(\frac{m{E}^{4}}{{\epsilon }^{2}}\right)$
This work	Trace distance	Bounded parameters condition	Heterodyne	${{\mathcal{O}}}\left(\frac{{m}^{3}\log m}{{\epsilon }^{2}}\right)$
This work	Hamiltonian learning	Bounded parameters condition, known graph	Heterodyne	${{\mathcal{O}}}\left(\frac{\log m}{{\epsilon }^{2+\gamma }}\right)\,\,\forall \gamma > 0$
This work	Graph learning	Bounded parameters condition, minimum interaction strength	Heterodyne	${{\mathcal{O}}}\left(\frac{\log m}{{\kappa }^{2+\gamma }}\right)\,\,\forall \gamma > 0$

All the protocols require *O*(poly(*m*)) computation time to learn the relevant parametrization. In the case of graph learning, the polynomial degree depends on the other parameters, obeying the bounded parameters condition.

Moreover, all of our results only require heterodyne measurements, which are easy to implement experimentally, and have polynomial postprocessing in the number of modes. As is well-known and explained in detail in Supplementary Note 3, given a Gaussian state *ρ* with covariance matrix *V*, performing a heterodyne measurement corresponds to sampling from a Gaussian random vector with covariance matrix given by *V* + *I*/2. Thus, by resorting to standard concentration inequalities of Gaussian random variables^67^, we show in Supplementary Note 3 that to estimate the entries of the covariance matrix it is sufficient to compute the empirical estimator of the covariance matrix of the heterodyne output and subtract *I*/2. This procedure is the bread and butter of all our algorithms, as we always depart from some estimate of *V*. In particular, we show that it suffices to take

9 $$N={{\mathcal{O}}}\left(\frac{{{\max }_{i}}{\left(2{V}_{i,i}+1+| {t}_{i}{| }^{2}\right)}^{2}{(1+{t}_{\max })}^{2}}{{\epsilon }^{2}}\ln \left(\frac{m}{\delta }\right)\right)$$

samples to obtain a covariance matrix estimate such that every entry differs from the corresponding true entry by at most *ϵ* with probability of success at least 1−*δ*.

The second ingredient that is present in our proofs are continuity bounds for the Hamiltonian in terms of the covariance matrix, and vice-versa, which are discussed in Supplementary Note 5. In fact, the key continuity bound for Hamiltonian learning actually involves ${K}_{i}:=2{(2i\Omega {V}_{i}-I)}^{-1}$. We show that, assuming that ∥*V*_1_−*V*_2_∥_*∞*_, respectively ∥*K*_1_−*K*_2_∥_*∞*_, are small with respect to a certain function of the parameters ${d}_{\max },\parallel S{\parallel }_{\infty }$ of the Hamiltonian *H*_1_, then the Hamiltonian difference is bounded as:

10 $$\parallel {H}_{1}-{H}_{2}{\parallel }_{\infty }\le {f}_{1}({d}_{\max },\parallel S{\parallel }_{\infty })\parallel {V}_{1}-{V}_{2}{\parallel }_{\infty },$$

11 $$\parallel {H}_{1}-{H}_{2}{\parallel }_{\infty }\le {f}_{2}({d}_{\max },\parallel S{\parallel }_{\infty })\parallel {K}_{1}-{K}_{2}{\parallel }_{\infty },$$

for some functions *f*_1_, *f*_2_. We also obtain a converse bound $\parallel {V}_{1}-{V}_{2}{\parallel }_{\infty }\le {f}_{3}({d}_{\max },{d}_{\min },\parallel S{\parallel }_{\infty })\parallel {H}_{1}-{H}_{2}{\parallel }_{\infty },$ for ∥*H*_1_ − *H*_2_∥_*∞*_ smaller than a function of ${d}_{\max },{d}_{\min },\parallel S{\parallel }_{\infty }$. These are analogous to bounds derived in ref. ^2^ for spin system, although our proofs are based on techniques from matrix analysis and the integral representation of the Hamiltonian (Eq. (6)), while theirs more on techniques from many-body systems. We believe that these continuity bounds are of independent interest. Finally, the key tool that makes Hamiltonian and graph learning efficient is the local inversion subroutine, which gives a sample- and computationally efficient estimate of (2*V*−*iΩ*)^−1^ from heterodyne measurements, which is overviewed in the “Methods” and analysed in detail in Supplementary Note 6.

We will first present our result on learning in trace distance. Roughly speaking (see Supplementary Definition 4.1 for a formal definition), the trace distance learning problem requires to output a covariance matrix or Hamiltonian and mean vectors such that the corresponding Gaussian state is *ϵ*-close in trace distance to the true state with probability at least 1−*δ*, given access to measurement data. It is analogous to the classical problem of estimating a multivariate Gaussian in total variation distance. We then show in Supplementary Note 4:

#### Theorem 1

(Learning Gaussian states in trace distance (informal)) Let *ρ*(*t*, *H*) be a Gaussian state on *m* modes. Then, for 1 > *ϵ*, *δ* > 0, it suffices to take

12 $$N={{\mathcal{O}}}\left({\epsilon }^{-2}{m}^{3}\ln (m{\delta }^{-1}){{\rm{poly}}}\left(\parallel S{\parallel }_{\infty },\frac{{d}_{\max }+{({e}^{2{d}_{\max }}-1)}^{2}}{{(1-{e}^{-2{d}_{\min }})}^{2}},{t}_{\max }\right)\right)$$

copies of *ρ* to obtain an estimate of *ρ* up to trace distance *ϵ* with success probability at least 1 − *δ*.

Note that this clarifies that an *ϵ*^−2^ scaling is possible for this task, answering an open problem in ref. ^53^. This fact was also observed by the concurrent work ref. ^55^. In fact, ref. ^53^ used a different parametrization than ours and expressed the sample complexity upper bounds in terms of an upper bound on the expected energy of the state (i.e., the expectation value of *E*_*m*_). Theorem 4 in ref. ^53^ shows an upper bound ${{\mathcal{O}}}(\frac{{m}^{7}{E}^{3}}{{\epsilon }^{4}})$, while Theorem 5 in the concurrent paper ref. ^55^ improved this to ${{\mathcal{O}}}\left(\frac{{E}^{4}}{{\epsilon }^{2}}\left(m+\log \frac{2}{\delta }\right)\right)$. We can express our bound in terms of an upper bound on the expectation value of the energy of the state to learn, and of a bound ${\beta }_{\max }\ge {d}_{\max }$ as $N={{\mathcal{O}}}\left(\frac{{m}^{3}{E}^{5}{({e}^{2{\beta }_{\max }}-1)}^{2}}{{\epsilon }^{2}}\log \frac{m}{\delta }\right)$, see Supplementary Remark 4.2. We see that we have to assume not only bounded energy but also bounded value of ${\beta }_{\max }$: highest values mean that the state is closer to being non-full rank, and in that case the Hamiltonian is not well-defined and our approach cannot work. This means, in particular, our approach does not cover pure state learning. We also mention that alternative trace distance bounds with ${{\mathcal{O}}}(\sqrt{\epsilon })$ scaling were obtained by refs. ^54,68^, who argued that while suboptimal for the trace distance, this behaviour is tight for the Bures distance^68^.

Our guarantee on Hamiltonian learning for Gaussian states is discussed in Supplementary Note 7. In essence, this problem asks us to recover the Hamiltonian *H* from measurements of *ρ* in operator norm, up to error *ϵ* > 0, with probability of success at least 1−*δ*. The result is stated here to highlight the joint dependence in the number of modes and precision in operator norm, while enforcing the bounded parameters condition. See Supplementary Corollary 7.1 for a more detailed account on the combined dependence on the parameters.

#### Theorem 2

(Gaussian Hamiltonian learning (informal)) Let *ρ* be a Gaussian state with Hamiltonian *H* with known graph *G* of maximal degree Δ−1 and such that the bounded parameters condition holds. Then, it suffices to take

13 $$N={{\mathcal{O}}}\left(\frac{1}{{\epsilon }^{2+\gamma }}\ln \left(\frac{m}{\delta }\right)\right)\,\,\forall \gamma > 0$$

copies of *ρ* to obtain an estimate $\hat{H}$ satisfying $\parallel H-\hat{H}{\parallel }_{\infty }\le \epsilon$ with probability of success at least 1−*δ*, using heterodyne measurements to estimate the covariance matrix and Algorithm LearnHamiltonianGaussian (Box 1) to reconstruct the estimate of the Hamiltonian.

#### Remark 1

The computational complexity of the above procedure is *O*(poly(*m*)) when the other parameters are fixed, dominated by the classical post-processing of LearnHamiltonianGaussian in Box 1, and with exponent of *m* independent from the other parameters. In fact, each of the *m* calls to the local inversion subroutine in Supplementary Definition 6.3 to output the estimator ${{\mbox{LI}}}_{{{\mathcal{N}}}}(2\hat{V}-i\Omega )$ requires a matrix inversion of a submatrix of $2\hat{V}-i\Omega$, and the final logarithm can be implemented in *O*(poly(*m*)) time, e.g., via Jordan decomposition of the matrix ${{\mbox{LI}}}_{\hat{{{\mathcal{N}}}}}(2\hat{V}-i\Omega )i\Omega$.

**Fig. 3:**
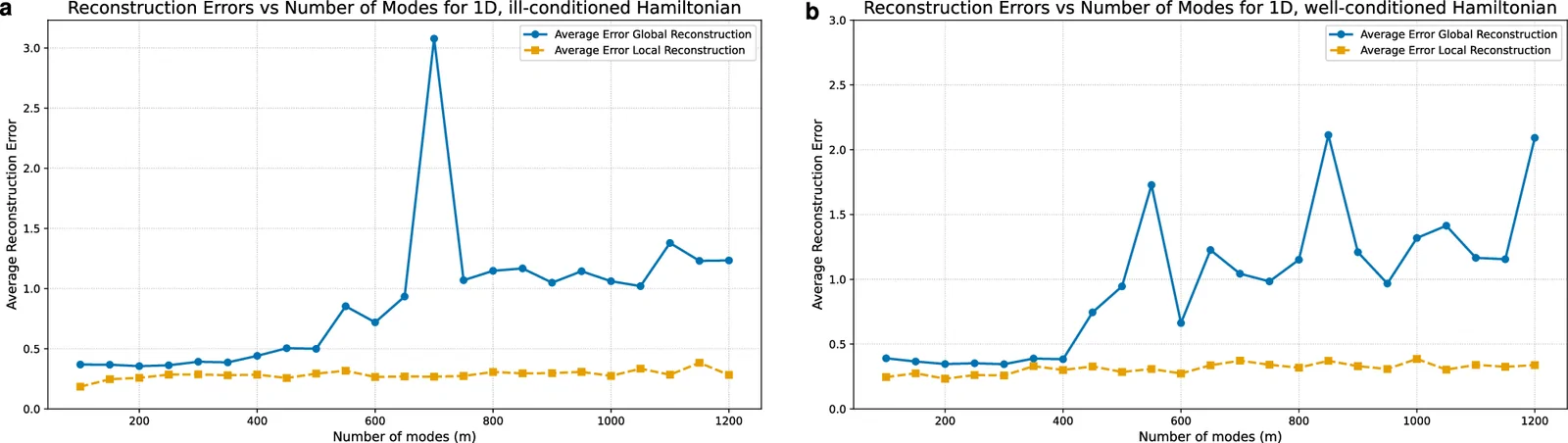
Reconstruction errors vs. number of modes for ill- and well-conditioned Hamiltonians. For this figure, we considered two variations of 1D-Hamiltonians $H=(2+c)I-| 0\left.\right\rangle \left\langle \right.0| -{\sum }_{i}(| i\left.\right\rangle \left\langle \right.i+1|+| i+1\left.\right\rangle \left\langle \right.i| )$ and compared the reconstruction using the global plug-in method (estimate $\hat{H}=\frac{1}{2}\log (I+2{(2\hat{V}-i\Omega )}^{-1}i\Omega )i\Omega$) versus the local method ($\hat{H}=\frac{1}{2}\log (I+2{{\mbox{LI}}}_{\hat{{{\mathcal{N}}}}}(2\hat{V}-i\Omega )i\Omega )i\Omega$) at inverse temperature *β* = 0.5. The well-conditioned Hamiltonian corresponds to *c* = 10^−1^ (**b**) and the ill-conditioned *c* = 0 (**a**), see Supplementary Note 11 for a discussion on the condition number of the Hamiltonian for *c* = 0 as a function of *m*. We performed numerical experiments generating 10^4^ samples for all number of modes and reconstructed the corresponding Hamiltonian for 5 different sets of samples for each number of modes. The *y*-axis displays the maximum difference ${{\max }_{(a,b):{H}_{ab}\ne 0}}| {H}_{ab}-{\hat{H}}_{ab}|$ between the estimated Hamiltonian and the ideal one averaged over 5 realizations of the samples. As they are both 1D models, we have Δ = 3 for both cases. Importantly, we see that the quality of the recovery in both cases is essentially independent of the number of modes for both the well and ill-conditioned case when using our local inversion method and that it outperforms the error of the global inversion by roughly one order of magnitude for larger numbers of modes.

**Fig. 4:**
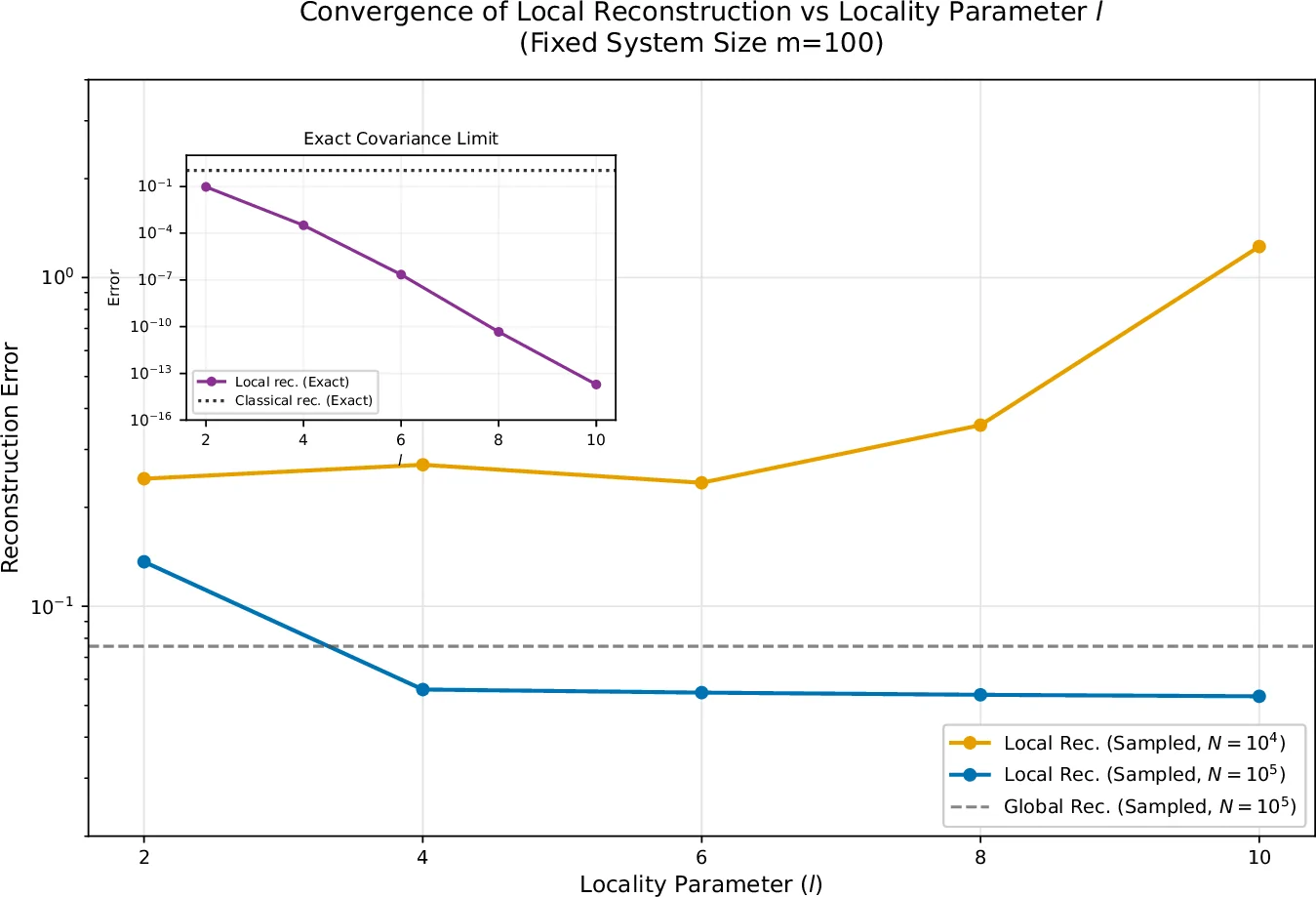
Reconstruction error vs. locality parameter. Entry-wise reconstruction errors vs. *l* for the 1D well-conditioned Hamiltonian defined in Fig. 3 for *N* = 10^5^ and *N* = 10^4^ samples, against the error of the global reconstruction. We see that the local strategy outperforms the global one at *l* = 4 and *N* = 10^5^. In the inset, we plot the error given the exact covariance matrix, showing the exponential improvement in precision as we increase *l*, together with the error obtained by estimating the Hamiltonian as (2*V*−*iΩ*)^−1^, i.e., the classical formula.

We emphasize the fact that the sample complexity is only logarithmic in the number of modes. Furthermore, we show that for graphs with polynomially growing neighborhoods, the sample complexity of our procedure in terms of precision is *ϵ*^−2^ up to polylog terms. In Fig. 3, we showcase numerical simulations of our method for a 1D Hamiltonian with up to 1200 modes, showing how it outperforms plug-in estimation methods. In addition, in Fig. 4, we show how truncating to nearest neighbors is not sufficient to obtain a good recovery even when we are not limited by finite sample-size.

Finally, we also show how to learn the graph of the Hamiltonian. In this problem, we are given access to copies of the Gaussian states and the promise that its Hamiltonian is supported on a graph $G=([m],{\mathsf{E}})$ of maximal degree Δ−1. Furthermore, we are given the promise that for each edge, at least one entry of the corresponding sub-matrix of the Hamiltonian is above a certain threshold *κ* > 0. The goal is then to estimate *G* with probability of success at least 1−*δ*. The dependence of the sample complexity of the graph learning algorithm on the parameters Δ, ∥*S*∥_*∞*_, ${d}_{\max }$, ${d}_{\min }$ is quite complex (even super-exponential), so we suppress it for simplicity in the next statement, which we show and state fully in Supplementary Note 8:

#### Theorem 3

(Learning the graph of Gaussian states, informal). Let *ρ* be a Gaussian state with Hamiltonian *H*, with graph *G* of degree Δ−1 and edge set ${\mathsf{E}}$, such that the bounded parameters condition holds and such that $0 < \kappa \le {{\min }_{(i,j)\in {\mathsf{E}}}}{{\max }_{{\delta }_{1},{\delta }_{2}\in \{0,1\}}}| {H}_{2i-{\delta }_{1},2j-{\delta }_{2}}|$. Then, it suffices to take

14 $$N={{\mathcal{O}}}\left(\frac{1}{{\kappa }^{2+\gamma }}\ln \left(\frac{m}{\delta }\right)\right)\quad \forall \gamma > 0$$

copies of *ρ* to learn the graph *G* with probability of success at least 1−*δ*, using heterodyne measurements to estimate the covariance matrix and LearnGraphGaussian (Box 2) to reconstruct the graph of the Hamiltonian.

#### Remark 2

The computational complexity of the above procedure is *O*(poly(*m*)) when the other parameters are fixed, dominated by the classical post-processing of LearnGraphGaussian in Box 2. While as in LearnHamiltonianGaussian in Box 1 all the operations are only functions of matrices of size at most 2 *m* × 2 *m*, the number of such operations is a polynomial in *m* whose depends on the other parameters. Most importantly, the loop between line 4 and 10 in Box 2 can require a number of local inversions as many as the number of pairs of distinct candidate neighborhoods of a vertex *i* of size Δ^*l*^. In the end, this gives an exponent of *m* which is super-exponential in Δ, exponential in ${d}_{\max }$ and subpolynomial in $\frac{1}{{d}_{\min }}$ and $\frac{1}{\kappa }$. See the proof of Supplementary Theorem 8.1 for more details.

To the best of our knowledge, there are no results in this direction for quantum spin systems. We once again emphasize the logarithmic scaling with the number of modes. Furthermore, even though the scaling with respect to other parameters is not efficient, this is a general feature of Hamiltonian learning algorithms in the quantum case.

#### Remark 3

Theorem 2 and Theorem 3 both assume the worst-case growth for the neighborhoods at distance *l* in a graph of degree Δ−1, i.e., Δ^*l*^. However, *r*-dimensional lattices have a growth *gl*^*r*^. By restricting to graphs with such polynomial growth of neighborhoods, one can obtain improved results, and in particular an ${{\mathcal{O}}}({\epsilon }^{-2}{\log }^{4r}({\epsilon }^{-1})\log (m))$ upper bounds on the sample complexity of Hamiltonian learning, see Supplementary Theorem 7.1. Also note that for gapped harmonic lattices, exponential correlation decay has been established in important subclasses^69,70^, but we do not need to use this fact (see Supplementary Remark 7.1 for a discussion). The same arguments may be used to obtain an $\tilde{{{\mathcal{O}}}}\left(\frac{1}{{\kappa }^{2}}\log m\right)$ sample complexity bound for graph learning with polynomially growing neighborhoods.

Box 1: Hamiltonian learning algorithm from local inversions for Gaussian states. The explicit choice of *l*, together with the required entrywise precision of $\hat{V}$, is given in Supplementary Note 7 **Algorithm**: LearnHamiltonianGaussian

1: Input: estimate $\hat{V}$ of the covariance matrix *V* of an *m*-mode Gaussian state *ρ*, graph *G*, distance parameter *l*

2: Set ${{\mathcal{N}}}={\{{{{\mathcal{N}}}}_{i}(l)\}}_{i\in [m]}$, where ${{{\mathcal{N}}}}_{i}(l)$ is the set of nodes at distance at most *l* from *i* in the graph *G*.

3: Compute the local inversion ${{\mbox{LI}}}_{{{\mathcal{N}}}}(2\hat{V}-i\Omega )$ of ${(2\hat{V}-i\Omega )}$ as in Definition 1

4: Compute the estimator $\hat{H}=f(2{{\mbox{LI}}}_{{{\mathcal{N}}}}(2\hat{V}-i\Omega ))$ of *H*, as in Eq. (6)

5: Output $$\hat{H}$$.

Box 2: Algorithm for learning the graph of a Gaussian state. The explicit choices of *l* and *η*, and the proof that the neighborhood test correctly reconstructs the graph, are given in Supplementary Theorem 8.1 **Algorithm**: LearnGraphGaussian

1: Input: estimate $\hat{V}$ of the covariance matrix *V* of *ρ*, distance parameter *l*, (upper bound on) cardinality of neighborhood *ξ*(*l*), threshold parameters *η*, *κ* > 0.

2: **for** *i* ∈ [*m*] **do**

3: **for** ${{{\mathcal{N}}}}_{i}$ candidate neighborhoods of *i* of cardinality *ξ*(*l*) **do**

4: set $${\hat{{{\mathcal{N}}}}}_{i}={{{\mathcal{N}}}}_{i}$$

5: **for** ${\overline{{{\mathcal{N}}}}}_{i}\in$ sets of vertices in $[m]/{{{\mathcal{N}}}}_{i}$ of cardinality *ξ*(*l*) **do**

6: Compute $$\{{{\mbox{LI}}}_{{{{\mathcal{N}}}}_{i}\cup {\overline{{{\mathcal{N}}}}}_{i}}{(2\hat{V}-i\Omega )}_{2i-{\delta }_{1},2j-{\delta }_{2}},j\in {\overline{{{\mathcal{N}}}}}_{i},{\delta }_{1},{\delta }_{2}\in \{0,1\}\}$$

7: **end for**

8: **if** $${\mathop{\max }\limits_{{\overline{{{\mathcal{N}}}}}_{i}}}{\mathop{\max }\limits_{j\in {\overline{{{\mathcal{N}}}}}_{i},{\delta }_{1},{\delta }_{2}\in \{0,1\}}}| {{\mbox{LI}}}_{{{{\mathcal{N}}}}_{i}\cup {\overline{{{\mathcal{N}}}}}_{i}}{(2\hat{V}-i\Omega )}_{2i-{\delta }_{1},2j-{\delta }_{2}}| \le 3\eta$$ **then**

9: **break**

10: **end if**

11: **end for**

12: **end for**

13: For each *i* ∈ [*m*], $j\in {\hat{{{\mathcal{N}}}}}_{i}$, eliminate element *j* from ${\hat{{{\mathcal{N}}}}}_{i}$ if $i\notin {\hat{{{\mathcal{N}}}}}_{j}$.

14: Compute the estimator $\hat{H}=f(2{{\mbox{LI}}}_{\hat{{{\mathcal{N}}}}}(2\hat{V}-i\Omega ))$ of *H*, as in Eq. (6).

15: Output the edges (*i*, *j*) such that ${{\max }_{{\delta }_{1}\in \{0,1\},{\delta }_{2}\in \{0,1\}}}| {\hat{H}}_{2i-{\delta }_{1},2j-{\delta }_{2}}| \ge \kappa /2$.

## Discussion

The present work establishes a foundation for studying the problem of learning quantum Gaussian graphical models. Several key challenges remain, which may require refinements of the techniques proposed or new approaches.

First of all, we make a remark on the (implicit) dependence of our result on the condition number of *H*. In the sample complexity for Hamiltonian and graph learning, we see a dependence on ${d}_{\min }$, which can be interpreted as a “maximum temperature” parameter of the Gaussian state. Since ${d}_{\min }$ gives a smaller contribution to the Hamiltonian matrix, it is natural to ask if this dependence can be removed. Although other quantum Hamiltonian learning results for discrete variable systems^1–3,12^ display polynomial dependence on the system size, they do not exhibit a dependence on a condition number. In fact, the dependence of our learning guarantees on the condition number can also be expressed in terms of potentially much smaller quantities, but we preferred to state our results as we did to avoid unnatural assumptions. Supplementary Remarks 4.1, 6.2, 7.2 clarify this point. In the analogous classical problem of learning the precision matrix (the term used in this context for the Hamiltonian matrix) or the graph, the dependence on the condition number was removed only by ref. ^31^, crucially using multiplicative error bounds on covariance estimation. In the graph learning problem, non-zero interactions respect a relative strength condition: two vertices (*i*, *j*) are connected if a Hamiltonian matrix element ${H}_{2i-\delta,2j-{\delta }^{{\prime} }}$ is sufficiently large compared with the diagonal elements ${H}_{2i-\delta,2i-{\delta }^{{\prime} }}$ and ${H}_{2j-\delta,2j-{\delta }^{{\prime} }}$ (here $\delta,\,{\delta }^{{\prime} }$ can be 0 or 1 and index the position and momentum quadratures). The sample complexity depends on the promise on the relative strength but not on the condition number. We leave the exploration of multiplicative error bounds in the quantum case for future work. See Supplementary Note 11 for an argument suggesting that with additive precision guarantees, one cannot avoid the condition number dependence.

Several natural developments of our work can be envisioned. The dependence of the sample complexity on *ϵ* and *κ* for Hamiltonian and graph learning, respectively, is not exactly the expected inverse quadratic one. While this can be remedied for graphs whose neighborhoods increase polynomially with distance, in the general case, this could be a limitation of our approach based on a Taylor expansion. It would be very interesting to understand if this can be overcome by other techniques. We also leave open the problem of finding lower bounds on the sample complexity of both Hamiltonian estimation and graph learning. It would be especially interesting to understand how these bounds differ from the ones that can be obtained in the classical case^32^. It would also be valuable to investigate whether our results can be extended to Gaussian fermionic states. Finally, we address the Hamiltonian learning problem by plugging-in estimates of the covariance matrix via a simple single-copy measurement procedure. It would be interesting to investigate if entangled measurements could be beneficial to the estimation of the covariance matrix and of the Hamiltonian matrix.

## Methods

### Parametrization

In this section, we discuss interpretations of our choice of parametrization appearing in our proofs, and connections with other possible choices. Operationally, the symplectic diagonalization (or Williamson’s normal form) corresponds to a preparation procedure for a Gaussian state: (1) prepare *m* thermal states of harmonic oscillators with inverse temperatures 2*d*_*i*_. (2) Act on them via the phase space transformation corresponding to the symplectic matrix *S*, which can be realized via a passive linear transformation, followed by single-mode squeezing, and by another passive linear transformation^40^. The operator norm ∥*S*∥_*∞*_ encodes the maximum amount of single-mode squeezing of this decomposition of *S*. Moreover, Hamiltonians that commute with harmonic oscillator energy operator ${E}_{m}=\frac{1}{2}{\sum }_{i=1}^{2m}{R}_{i}{R}_{i}$ (and thus with the particle number) have the form ${\sum }_{i,j}{a}_{i}^{{\dagger} }{K}_{ij}{a}_{j}$, where we expressed position and momentum operators in terms of creation and annihilation operators. Here, *K* must be a complex positive-semidefinite matrix, and it is diagonalized by a unitary matrix. As a symplectic transformation of the phase space, this is an orthogonal symplectic transformation, and it has ∥*S*∥_*∞*_ = 1. Let us also denote as ${d}_{\min }$ and ${d}_{\max }$ the smallest and the largest symplectic eigenvalues of *H*, respectively, and let ${\lambda }_{\min }$ and ${\lambda }_{\max }$ be the smallest and the largest eigenvalues of *H*, respectively. By Theorem 11 in ref. ^71^, ${\lambda }_{\min }\le {d}_{\min }\le {d}_{\max }\le {\lambda }_{\max }$, and since ${\lambda }_{\max }=\parallel H{\parallel }_{\infty }\ge \parallel S{\parallel }_{\infty }^{2}{d}_{\min }\ge \parallel S{\parallel }_{\infty }^{2}{\lambda }_{\min }$, we have $1\le \parallel S{\parallel }_{\infty }^{2}\le \frac{{\lambda }_{\max }}{{\lambda }_{\min }}$. We can also bound ∥*S*∥_*∞*_, ${d}_{\min }$ and ∥*t*∥_2_ as a function of the energy of the state, i.e., the expectation value of operator *E*_*m*_, see Supplementary Note 10 for a proof:

15 $$\parallel t{\parallel }_{2}^{2}\le 2E,$$

16 $$\parallel S{\parallel }_{\infty }^{2}\le 4E,$$

17 $${(1-{e}^{-2{d}_{\min }})}^{-1}\le 8{E}^{2}.$$

However, ${d}_{\max }$ is essentially independent of the energy and expresses how close the state is to a pure state. In fact, for a sequence of states converging to the vacuum, with ${d}_{\max }={d}_{\min }=d$, *S* = *I*, *d* going to infinity, the energy goes to a constant.

### Trace distance learning

The main idea is to employ the symmetrized form of Pinsker’s inequality to upper-bound the trace distance between two Gaussian states in terms of their (symmetrized) relative entropies:

18 $$\parallel \rho -\hat{\rho }{\parallel }_{1}\le \sqrt{D(\rho \parallel \hat{\rho })+D(\hat{\rho }\parallel \rho )}\,.$$

We are then able to obtain a closed formula for the relative entropy, yielding the expression:

19 $$\parallel \rho -\hat{\rho }{\parallel }_{1}\le \sqrt{\mathop{\sum}_{i,j}\left[({\hat{H}}_{i,j}-{H}_{i,j})({V}_{i,j}-{\hat{V}}_{i,j})+({t}_{i}-{\hat{t}}_{i})({t}_{j}-{\hat{t}}_{j})({H}_{i,j}+{\hat{H}}_{i,j})\right]}$$

Then, using our estimators for the covariance matrix and the mean vector, combined with the continuity estimate in Eq. (10) for the Hamiltonian entries in terms of the covariance matrix, we are able to upper-bound the trace distance and get the advertised sample complexity.

### Hamiltonian learning

Recall that, given the covariance matrix *V*, the Hamiltonian of the Gaussian is given by $H=\frac{1}{2}\ln \left(I+\frac{2}{2i\Omega V-I}\right)i\Omega$. Thus, it is possible to infer the Hamiltonian if we have a sufficiently accurate approximation of the inverse of 2*V*−*i*Ω. These observations and the continuity estimates from Eqs. (10) and (11) already suffice to obtain a Hamiltonian learning protocol with polynomial sample complexity in the number of vertices. The main reason for the polynomial scaling is that this plug-in strategy requires us to estimate each entry of the covariance matrix to polynomial precision to ensure that the error in operator norm is of order *ϵ* once we perform the inversion.

However, based on classical results^28,30^, we expect to be able to solve the Hamiltonian learning problem with a number of samples that scales logarithmically with the number of modes. To achieve such a scaling, we pioneer what we call the local inversion technique. The intuition behind the technique is that, for local Gibbs states, the correlations with neighboring sites should determine the interactions. For classical Gaussian graphical models, this is true by conditional independence, so it is possible to completely determine the Hamiltonian matrix by just inverting blocks of the covariance matrix of constant-sized neighborhoods around each vertex. While this fails in the quantum case, we nevertheless show that for Gaussian quantum states it suffices to post-process an estimate of (2*V*−*i*Ω)^−1^, constructed such that matrix entries corresponding to a pair of vertices are obtained from inverting submatrices of the covariance matrix corresponding to vertices that are at most $\sim \frac{\log ({\epsilon }^{-1})}{\log \log ({\epsilon }^{-1})}$ away from the target pair of vertices, to estimate the interactions with error up to *ϵ*. To show this, we resort to various bounds on the error in approximating inverses of approximately sparse matrices with local inversions, and the Taylor series representation of (2*V*−*i*Ω)^−1^ in terms of the Hamiltonian, namely

20 $${(2V-i\Omega )}^{-1}=-\frac{I-{e}^{+2Hi\Omega }}{2}(i\Omega )=\frac{1}{2}{\sum }_{n=1}^{\infty }\frac{{\left(2Hi\Omega \right)}^{n}}{n!}(i\Omega ) \\=\frac{1}{2}{\sum }_{n=1}^{l}\frac{{\left(2Hi\Omega \right)}^{n}}{n!}(i\Omega )+E\,,$$

with $\parallel E{\parallel }_{\infty }\le \frac{\parallel S{\parallel }_{\infty }^{2}}{2}\left(\frac{{(2{d}_{\max })}^{l+1}{e}^{2{d}_{\max }}}{(l+1)!}\right)$, such that the parameter *l* controls both the sparsity of the approximation of (2*V*−*i*Ω)^−1^ and the approximation error. The required *l* to make ∥*E*∥_*∞*_ small enough follows from standard bounds on the Lambert *W* function^72^.

To define the local inversion, we use the notion of *neighborhood structure*: A family ${{\mathcal{N}}}={\{{{{\mathcal{N}}}}_{i}\}}_{i\in [m]}$ of subsets of [*m*] is called a neighborhood structure if for all *i* ∈ [*m*], $i\in {{{\mathcal{N}}}}_{i}$ and for all *i*, *j* ∈ [*m*], $i\in {{{\mathcal{N}}}}_{j}\Rightarrow j\in {{{\mathcal{N}}}}_{i}$. We use the term neighborhood to follow the convention used in classical papers on learning graphical models. But we emphasize that the ${{{\mathcal{N}}}}_{i}$ will *not* be the neighborhood of a vertex *i* of the interaction graph. Rather, the ${{{\mathcal{N}}}}_{i}$ will often correspond to candidates for the set of all vertices at most a certain distance away from *i*. Given a neighborhood structure, we will construct approximate inverses of matrices by “stitching together” the inverses of various submatrices with only entries in the neighborhood structure. More formally, we have:

#### Definition 1

(Local inversion). Let *N* be a Hermitian 2 *m*× 2 *m* matrix and let ${{\mathcal{N}}}={\{{{{\mathcal{N}}}}_{i}\}}_{i\in [m]}$ be a neighborhood structure of [*m*]. We denote by ${{\mbox{LI}}}_{{{\mathcal{N}}}}(N)$ the matrix constructed in the following way: for each *i* ∈ [*m*], let *N*_*i*_ be the $2| {{{\mathcal{N}}}}_{i}| \times 2| {{{\mathcal{N}}}}_{i}|$ matrix (assumed to be invertible) with entries ${N}_{2{j}_{1}-\delta,2{j}_{2}-{\delta' }_{2}}$ corresponding to indices ${j}_{1},{j}_{2}\in {{{\mathcal{N}}}}_{i}$, $\delta,{\delta }^{{\prime} }\in \{0,1\}$ (i.e., we select modes in ${{{\mathcal{N}}}}_{i}$ and regard the resulting principal submatrix as retaining the corresponding global row and column labels). Then, for each *i* ∈ [*m*], $j\in {{{\mathcal{N}}}}_{i}$ and $\delta,{\delta }^{{\prime} }\in \{0,1\}$, we define ${({{\mbox{LI}}}_{{{{\mathcal{N}}}}_{i}}(N))}_{2i-\delta,2j-{\delta }^{{\prime} }}$ to be ${({N}_{i}^{-1})}_{2i-\delta,2j-{\delta }^{{\prime} }}$ and ${\left({{\mbox{LI}}}_{{{\mathcal{N}}}}(N)\right)}_{2i-\delta,2j-{\delta }^{{\prime} }}$ to be equal to $\frac{1}{2}\left[{\left({N}_{i}^{-1}\right)}_{2i-\delta,2j-{\delta }^{{\prime} }}+{\left({N}_{j}^{-1}\right)}_{2j-{\delta }^{{\prime} },2i-\delta }^{*}\right]$, and set the other entries of ${{\mbox{LI}}}_{{{\mathcal{N}}}}(N)$ to 0. Note that, by construction, ${{\mbox{LI}}}_{{{\mathcal{N}}}}(N)$ is self-adjoint, as both $({N}_{i}^{-1})$ and $({N}_{j}^{-1})$ are.

The analysis of the algorithm is in Supplementary Notes 6 and 7. Crucially, the precision with which we need to estimate each of these submatrices to successfully invert them locally does not scale with the number of modes, and together with the continuity bound from Eq. (11), this implies our Hamiltonian learning result.

### Graph learning

The arguments to obtain our graph learning result also mostly rely on our local inversion techniques. The graph learning algorithm takes as input an estimate of the covariance matrix $\hat{V}$, a promised lower bound on the strength of interaction *κ* > 0, a promised upper bound on the size of neighborhoods of distance $l\, \sim \frac{\log ({\kappa }^{-1})}{\log \log ({\kappa }^{-1})}$ of vertices, denoted by *ξ*(*l*) ≤ Δ^*l*^, and a threshold parameter *η*, which depends on ∥*S*∥_*∞*_ and the symplectic eigenvalues. It then iterates over all vertices *i* and all possible neighborhoods of size *ξ*(*l*), denoted by ${{{\mathcal{N}}}}_{i}$. For each of the ${{{\mathcal{N}}}}_{i}$, we then consider all possible neighborhoods enlarged by *ξ*(*l*) additional vertices, defining a set ${\overline{{{\mathcal{N}}}}}_{i}$, and perform our local inversion with the enlarged neighborhood. If we see that in the resulting matrix there are no large entries (as measured by the threshold *η*) on row *i* and vertices $j\in {\overline{{{\mathcal{N}}}}}_{i}$, we then set our neighborhood as ${{{\mathcal{N}}}}_{i}$. The intuition behind the procedure is as follows: recall that we assume that we have a lower bound on the size of all interactions and that matrix elements of (2*V*−*i*Ω)^−1^ corresponding to vertices that are far apart decay rapidly. Furthermore, performing local inversions with entries at a distance $l \sim \frac{\log ({\eta }^{-1})}{\log \log ({\eta }^{-1})}$ from a vertex suffices to obtain a good recovery of the entries up to error ~*η*, which scales roughly as *κ*. If the *l*-neighborhood of *i* is contained in ${{{\mathcal{N}}}}_{i}$, then all the estimates with the *ξ*(*l*)-enlarged neighborhood will be correct, and all the entries added will be small because of the decay. Thus, we will correctly accept in this case. On the other hand, if we have the incorrect or incomplete *l*-neighborhood, then there are *ξ*(*l*) vertices we can add that will make it complete. For that joint neighborhood, we see two possibilities: either this will “turn on” a large entry, which will show us that we were not operating with the right neighborhood. In contrast, if we see that for all possibilities we add this never occurs, then we can also conclude that the vertices we are missing will only add a small error and can be neglected. By iterating over all such enlarged neighborhoods, we are able to successfully find submatrices to locally invert the covariance matrix for each vertex. After that, we inspect the resulting Hamiltonian and discard the interactions we find to be below the threshold given to us. Here, too, we are able to obtain a logarithmic scaling in the number of modes because we only need to invert locally.

## Supplementary information

Supplementary Information

Transparent Peer Review file

## Data Availability

This study did not generate or analyze any datasets.

## Code availability

The code used to generate the figures in this paper is available on GitHub^73^.
