# Supplementary material for: Efficient Hamiltonian, structure and trace distance learning of Gaussian states
Source: Nat Commun. 2026 Sep 16;17:10898. doi: 10.1038/s41467-026-77405-3 (PMC13677717; doi:10.1038/s41467-026-77405-3)
Supplement: Supplementary file 1 [file 41467_2026_77405_MOESM1_ESM.pdf]

Efficient Hamiltonian, structure and trace distance learning of Gaussian states
Supplementary Information

Marco Fanizza[*†‡] Cambyse Rouzé[§] Daniel Stilck França [¶‖]

August 18, 2026

In this Supplementary Information we provide formal statements and proofs of the results presented in the main text. See Table 1 for a summary of the notation, the interpretations of the various parameters we use, and the relations among them.

Contents

1 Supplementary note 1: Notations **2**

2 Supplementary note 2: Physical motivation and related work **4**
2.1 Bosonic graphical models in many-body physics 4
2.2 Related work on Gaussian graphical models 7

3 Supplementary note 3: Estimating the entries of the covariance matrix **8**

4 Supplementary note 4: Learning in trace distance **11**

5 Supplementary note 5: Continuity bounds for Hamiltonians and covariances **14**

6 Supplementary note 6: Sparse approximations of $(2V - i\Omega)^{-1}$ **17**

7 Supplementary note 7: Gaussian Hamiltonian learning **24**

8 Supplementary note 8: Learning the graph **27**

9 Supplementary note 9: Symplectic diagonalization and bounds on singular values **35**

[*]Inria, Télécom Paris - LTCI, Institut Polytechnique de Paris, 91120 Palaiseau, France. marco.fanizza@inria.fr

[†]Department of Mathematical Sciences, University of Copenhagen, Universitetsparken 5, 2100 Denmark

[‡]Física Teòrica: Informació i Fenòmens Quàntics, Departament de Física, Universitat Autònoma de Barcelona, ES-08193 Bellaterra (Barcelona), Spain

[§]Inria, Télécom Paris - LTCI, Institut Polytechnique de Paris, 91120 Palaiseau, France. rouzecambyse@gmail.com

[¶]Department of Mathematical Sciences, University of Copenhagen, Universitetsparken 5, 2100 Denmark. dsfranca@math.ku.dk

[‖]Univ Lyon, ENS Lyon, UCBL, CNRS, Inria, LIP, F-69342, Lyon Cedex 07, France

10 Supplementary note 10: Energy bounds **37**

11 Supplementary note 11: Family of Hamiltonians with condition number scaling with the size of the graph **38**

1 Supplementary note 1: Notations

We use the notation $[m]$ when referring to the set $\{1, \ldots, m\}$. We say that a square matrix $A \in M_{m,m}(\mathbb{C})$ is positive semidefinite (psd) if it is Hermitian with nonnegative eigenvalues, and we write $A \geq B$ to say that $A - B$ is psd. A psd matrix is positive definite if the eigenvalues are strictly positive. We denote by $O^\dagger$ the adjoint of a matrix $O \in M_{m_1,m_2}(\mathbb{C})$, and by $A^\intercal$ the transpose. We also denote by $\|A\|_p = \mathrm{tr}\left[(A^\dagger A)^{p/2}\right]^{\frac{1}{p}}$ the Schatten p-norm of a matrix A, and by $M_{2m}^{>0}(\mathbb{R})$ the set of positive-definite symmetric matrices of size $2m \times 2m$. The identity map is denoted by I. The set of real symplectic matrices of size $2m \times 2m$ is denoted by $\mathrm{Sp}(2m, \mathbb{R})$. We will work with separable Hilbert spaces, and we denote the trace of a trace-class operator by $\mathrm{tr}\left[\cdot\right]$.

Let us now review basic concepts of continuous variable systems and explain the connection between Gaussian quantum states and classical Gaussian graphical models. We refer to [1] for a more complete treatment and proofs of the statements here. A continuous variable (CV) system with m modes is defined on the Hilbert space $\mathcal{H}_m := L^2(\mathbb{R}^m)$, and we denote the canonical operators on each mode as X_j, P_j $(j = 1, \ldots, m)$. Define the formal vector

$$R := \left(X_1, P_1, \ldots, X_m, P_m\right)^\intercal$$

and the symplectic form

$$\Omega := \bigoplus_{i=1}^{m} \begin{pmatrix} 0 & 1 \\ -1 & 0 \end{pmatrix}$$

(this is a block diagonal matrix, where all diagonal blocks are 2×2 matrices), in terms of which the canonical commutation relations read (at least when evaluated on Schwartz functions)

$$[R_j, R_k] = i\Omega_{jk}\,. \tag{1}$$

With a slight abuse of notation, we will often denote by the same symbol Ω the symplectic form for different sets of modes. The quantum covariance matrix $V[\rho]$ and mean vector $t[\rho]$ associated with a generic state ρ are defined by

$$t[\rho]_j := \mathrm{tr}\left[\rho R_j\right]\,, \quad V[\rho]_{jk} := \frac{1}{2}\,\mathrm{tr}\left[\rho\left\{R_j - t_j,\, R_k - t_k\right\}\right]\,, \tag{2}$$

provided that these expressions are well defined. By the canonical commutation relations, we have that

$$V[\rho] + \frac{i}{2}\Omega = \mathrm{tr}\left[(R - t[\rho])\rho(R - t[\rho])\right]\,.$$

This relation as well as its transpose imply the following uncertainty relation:

$$V[\rho] \geq \pm\frac{i}{2}\Omega\,.$$

The condition above is in fact not only necessary but also sufficient for a matrix V to be the covariance matrix of a quantum state. For an arbitrary $x \in \mathbb{R}^{2m}$, we define the associated Weyl operator by

$$\mathcal{W}(x) := e^{ix^\intercal R} . \tag{3}$$

Note that $\mathcal{W}(x)^\dagger = \mathcal{W}(x)^{-1} = \mathcal{W}(-x)$. Using displacement operators, we can rewrite (1) in Weyl form as

$$\mathcal{W}(x+y) = e^{\frac{i}{2}x^\intercal \Omega y}\, \mathcal{W}(x)\mathcal{W}(y) . \tag{4}$$

This implies that

$$\mathcal{W}(-\Omega^{-1}x)^\dagger R W(-\Omega^{-1}x) = R + xI .$$

For an arbitrary trace-class operator Z, we construct its characteristic function $\chi_Z : \mathbb{R}^{2m} \to \mathbb{C}$ by

$$\chi_Z(x) := \operatorname{tr}\left[Z\, \mathcal{W}(x)\right] . \tag{5}$$

Characteristic functions are always bounded and furthermore continuous, because of the strong operator continuity of the mapping $x \mapsto \mathcal{W}(x)$. A Gaussian state ρ is a state whose characteristic function has the Gaussian form

$$\chi_\rho(x) = \exp\left(it[\rho]^\intercal x - \frac{1}{2}x^\intercal V[\rho]x\right) .$$

It is easy to verify in that case that $t[\rho]$ and $V[\rho]$ correspond to the mean vector and covariance matrix of ρ.

A Gaussian state is nondegenerate if and only if (see [1, Lemma 12.22])

$$\det\left(V[\rho] + \frac{i}{2}\Omega\right) \neq 0 ,$$

that is, if the matrix $V[\rho] + \frac{i}{2}\Omega$ is nondegenerate. In that case, the state ρ takes the form [1, Theorem 12.23]

$$\rho(t,H) = \mathcal{W}\big(-\Omega^{-1}t[\rho]\big)\, \frac{e^{-R^\intercal H[\rho] R}}{\sqrt{\det\left(V[\rho] + \frac{i}{2}\Omega\right)}}\, \mathcal{W}\big(-\Omega^{-1}t[\rho]\big)^\dagger , \tag{6}$$

where the matrix $H[\rho]$ is referred to as the (Gaussian) Hamiltonian of the system and satisfies

$$2i\Omega V[\rho] = \coth(iH[\rho]\Omega) . \tag{7}$$

Inverting it, we have

$$H[\rho] = \frac{1}{2}\ln\left(\frac{2i\Omega V[\rho] + I}{2i\Omega V[\rho] - I}\right) i\Omega = \frac{1}{2}\ln\left(I + \frac{2}{2i\Omega V[\rho] - I}\right) i\Omega. \tag{8}$$

A fundamental property of H that we will repeatedly use is the following. For any (symmetric) $2m \times 2m$ Hamiltonian $H > 0$, let a symplectic diagonalization be

$$H = S^{-\intercal} D S^{-1} , \tag{9}$$

with $D = \oplus_{i=1}^m d_i I_{M_2}$, with $d_i > 0$, and $S \in \mathrm{Sp}(2m, \mathbb{R})$. Operationally, the symplectic diagonalization (or Williamson's normal form) corresponds to a preparation procedure for a Gaussian state: 1) prepare m thermal states of harmonic oscillators with inverse temperatures $2d_i$. 2) Act on them via the phase space transformation corresponding to the symplectic matrix S, which can be realized via a passive linear transformation, followed by single-mode squeezing, and by another passive linear transformation [2]. The operator norm $\|S\|_\infty$ encodes the maximum amount of single-mode squeezing of this decomposition of S. Moreover, Hamiltonians that commute with the total energy $E_m = \frac{1}{2}\sum_{i=1}^{2m} R_i R_i$ have the form $\sum_{i,j} a_i^\dagger K_{ij} a_j$, where we expressed position and momentum operators in terms of creation and annihilation operators. Here K must be a complex psd matrix and it is diagonalized by a unitary matrix. As a symplectic transformation of the phase space, this is an orthogonal symplectic transformation and it has $\|S\|_\infty = 1$. Let us also denote as $d_{\min}$ and $d_{\max}$ the smallest and the largest symplectic eigenvalues of H, respectively, and let $\lambda_{\min}$ and $\lambda_{\max}$ be the smallest and the largest eigenvalues of H, respectively. By Theorem 11 in [3], $\lambda_{\min} \le d_{\min} \le d_{\max} \le \lambda_{\max}$, and since $\lambda_{\max} = \|H\|_\infty \ge \|S\|_\infty^2 d_{\min} \ge \|S\|_\infty^2 \lambda_{\min}$, we have $1 \le \|S\|_\infty^2 \le \frac{\lambda_{\max}}{\lambda_{\min}}$. In the following, $\|S\|, d_{\min}, d_{\max}$ will be used as parameters, as they have a natural physical interpretation, but these inequalities clarify how they relate to more usual parameterization of Hamiltonian matrices, in terms of their norm and condition number. We will say that a Hamiltonian matrix H has graph $G = ([m], \mathsf{E})$ made of m vertices, where two vertices $i, j \in [m]$ are connected by an edge whenever the 2×2 sub-matrix $\left\{H_{2i-\delta, 2j-\delta'}\right\}_{\delta,\delta' \in \{0,1\}}$ is non-zero.

We can also bound $\|S\|_\infty$, $d_{\min}$ and $\|t\|_2$ as a function of the energy of the state, i.e. the expectation value of operator E_m, see Section 10.

However, $d_{\max}$ is essentially independent of the energy, and expresses how close the state is to a pure state. In fact, for a sequence of states converging to the vacuum, with $d_{\max} = d_{\min} = d$, $S = I$, d going to infinity, the energy goes to a constant.

2 Supplementary note 2: Physical motivation and related work

2.1 Bosonic graphical models in many-body physics

Bosonic Hamiltonians with a finite number of modes are used to describe a multitude of interacting many-body quantum systems, such as cold atoms in optical lattices, magnetization fields, optomechanical systems, trapped ions, and electromagnetic fields in cavities, interacting with matter [2]. Among those, sparse Hamiltonians have been extensively considered for bosonic systems, as most materials are naturally described by Hamiltonians with a lattice structure, where the interaction strength decays with the distance. The sparse Hamiltonians considered in this work are models where the interactions are assumed to be zero if vertices are not connected, for example because the corresponding sites are further than some fixed distance on a lattice. This includes the approximation of considering non-zero interactions only between nearest neighbors. A key example of this is a grid of harmonic oscillators, an idealization which is a cornerstone of theoretical physics, since any potential can be approximated around its minimum by a quadratic one. Moreover, in systems with higher order-interactions, the Bogoliubov-De Gennes approach is used to study excitations above the ground state, via an effective quadratic Hamiltonian. This method captures the physics of weakly-interacting dilute Bose gases [4]. Moreover, a typical quadratic Hamiltonian is also the hopping (or tight-binding) Hamiltonian on a graph, which is the basis of all lattice models. The sharp cutoff on local interactions is also quite convenient for learning questions because it reduces

Symbol	Description	Useful Relations
m	Number of modes	-
H	Hamiltonian matrix	-
Δ	Degree of the interaction graph $+1$	$\|H\|_\infty \leq 2\Delta \max_{i,j} \|H_{i,j}\|$
E	Edges of the interaction graph	$0 < \max_{\delta_1,\delta_2\in\{0,1\}} \|H_{2i-\delta_1,2j-\delta_2}\|$ iff $(i,j) \in \mathsf{E}$
κ	Lower bound on absolute values of Hamiltonian interaction matrix elements	$\kappa < \max_{\delta_1,\delta_2\in\{0,1\}} \|H_{2i-\delta_1,2j-\delta_2}\|$ for any $(i,j) \in \mathsf{E}$.
D, S	Williamson's normal form of H	$H = S^{-\intercal} D S^{-1}$
V	Covariance matrix	$V = \frac{1}{2} S \coth(D) S^\intercal$
t	Vector of first moments	-
E	Expectation value of the total energy	$\mathrm{tr}[V] + \|t\|_2^2 = 2E$
$d_{\max}$	Maximum symplectic eigenvalue of H, maximum inverse temperature of a normal mode, closeness to non-full-rank states	$\|D\|_\infty = d_{\max} \leq \|H\|_\infty$
$d_{\min}$	Minimum symplectic eigenvalue of H, minimum inverse temperature of a normal mode, spectral gap of $R^\intercal H R$	$\|D^{-1}\|_\infty = d_{\min}^{-1} \leq \|H^{-1}\|_\infty$ $(1 - e^{-2d_{\min}})^{-1} \leq 8E^2$
$\|S\|_\infty$	Operator norm of S, maximum amount of squeezing	$1 \leq \|S\|_\infty^2 \leq \frac{\|H\|_\infty}{d_{\min}}$ $\|S\|_\infty = \|S^{-1}\|_\infty$ $\|S\|_\infty^2 \leq 4E$

Table 1: Notation and relations between the several parameters used in the analysis. Replication of Table 1 in the main text.

the number of parameters.

In fact, experimentally realizing local (and thus sparse) Hamiltonians, including bosonic ones, is the goal of analog quantum simulation [4], both for reproducing models of condensed matter physics and for creating and studying meta-materials that cannot be found in nature. Starting with proposals for simulating the Bose-Hubbard model in cold atoms [5], the field has also explored Hamiltonians with non-Euclidean interaction graphs, which exhibiting non-trivial band structures arising from their topology [6, 7].

With current technology, it is in fact possible to realize simulators that implement Hamiltonians with a desired graph structure, in a variety of platforms and in a programmable way. Perhaps the closest work to our idealized setting is [8], which reports the engineering of various interaction graphs, including rings, chains, ladders, non-Archimedean geometries, in an array of atomic ensembles with 18 sites and 10^4 Rubidium atoms per site, interacting via spin-exchange. The collective magnetizations at each site can be treated as bosonic variables in the relevant regime, with a quadratic

Hamiltonian. The paper also shows procedures to verify the interactions from two-point correlation functions of the magnetization, using a combination of symmetries of their model, an ansatz on the covariance matrix, and a high inverse temperature approximation, estimating the interaction matrix as the inverse of the covariance matrix – i.e. the classical method, expected to be valid at high temperature. Our methods give a way to estimate the interactions and the graph in these types of systems that is applicable at lower temperatures and without model-specific assumptions. We also mention [9], where quadratic bosonic Hamiltonians on programmable graphs have also been simulated via photonic synthetic lattices with up to 10^5 sites, where frequency modes are mapped to lattice sites, and [10], where lattice bosonic Hamiltonians, including honeycomb lattice structure, have been engineered on 10 sites of a superconducting optomechanical system, and the quadratic Hamiltonian is reconstructed with modeshape measurements. Finally, [11] is a theoretical proposal for bosonic Hamiltonian simulation using phonons in trapped-ion crystals, interacting via the excitations of the trapped ion spins. In all the above, quadratic hopping Hamiltonians already explore interesting physics and testing their correct implementation is essential for progressing toward the simulation of interacting models.

The problem of Gaussian Hamiltonian learning can also be seen as a non-asymptotic instance of Gaussian multiparameter metrology, for which studies on the asymptotic achievable precision exist (via the Fisher information matrix, e.g. [12]). Indeed, our algorithms also suggest many-body metrology applications [13]: without trying to achieve the optimal information-theoretic limits which are highly sensitive to precise values of the parameters themselves, assumed to be roughly known, our methods ensure that the estimate of any parameter encoded linearly in the Hamiltonian matrix is robust with respect to uncertainties in the other parameters, since the full Hamiltonian can be estimated at once, with non-asymptotic guarantees. As an example, bosonic Gaussian systems are experimentally used as thermometric probes [14]. Existing works on this (see e.g. [15]) optimize over feasible measurements (including those we use in our protocol) to infer the temperature of a Gaussian state departing from a *known* value.

It is also worth discussing our assumption that the Gibbs states of quadratic Hamiltonians are a natural resource to consider for a learning problem. In the theory of open quantum systems, the fact that Gibbs states are thermal equilibrium states can be justified through the microscopic derivation of the Lindblad master equation from an interaction with a large bath, using the Born-Markov and secular approximations [16]. The result of this procedure is a time evolution that has the Gibbs state of the system Hamiltonian as a fixed point and, if a certain ergodicity condition is satisfied, converges to the Gibbs state. While this approximation has had tremendous success in quantum optics, it is not well justified in other settings, especially when the system of interest is large, see e.g. [17] where other approximation methods are proposed. Nevertheless, a recent paper showed a model that gives relaxation to Gibbs states for generic quadratic fermionic or bosonic models coupled to generic Gaussian baths satisfying detailed balance, where uniform interactions avoid the need of the secular approximation [18]. We would also stress that for reconstructing the Hamiltonian, it would be enough that first and second moments thermalize to those of the Gibbs state, and that the covariance estimator stays sub-exponential. The former assumption is at the core of less strict definitions of thermalization [19], while the latter is a very reasonable assumption for imperfect implementations of heterodyne measurements.

2.2 Related work on Gaussian graphical models

The problem of estimating Gaussian graphical models with sparsity is a central theme of high-dimensional statistics, and we refer to books such as [20, 21, 22] for a more in-depth history of the problem. When the graph is known, a simple procedure using local inversions for each neighborhood suffices to find the coefficients very efficiently, as explained in Chapter 17 of [20]. In the zero-mean case, $O(\frac{d}{\epsilon^2})$ samples are sufficient to learn a $d \times d$ covariance matrix in relative error, i.e. $\Sigma(1-\epsilon) \leq \hat{\Sigma} \leq \Sigma(1+\epsilon)$ [22]. Applying local inversion to each neighborhood, symmetrizing, and via union bound, this means that $O(\frac{\Delta\|\Theta\|_\infty^2}{\epsilon^2} \log m)$ samples guarantee an entrywise error for the entries of $\Theta = \Sigma^{-1}$ of order $O(\epsilon)$, thus with no condition number dependence. It seems to us that a slight dependence on the diagonal elements of V is instead needed in the non-zero mean case, which is not always treated in the literature, but to learn the precision matrix alone, one can simply subtract copies of samples to get a zero-mean Gaussian with twice the covariance matrix. An essentially matching lower bound (in the dependence on ϵ and $\log m$) to this simple algorithm is given by Theorem 2 of [23], which states that $\Omega(\frac{\log(m\Delta)}{\epsilon^2})$ samples are necessary. Let us thus stress that local inversions on neighborhoods at distance 1 are already sufficient in the classical case because conditional independence ensures that the conditional distribution of a random variable X_i, when the other variables at distance one in the graph are known, contains the full information about the interactions of i with its first neighbors. Our local inversion method, instead, goes further in the graph to account for the lack of conditional independence.

In fact, most of the literature has directly dealt with the harder problem of estimating the precision matrix when the graph is not known, as well as estimating the graph itself. To do that, the main strategy is to build an estimator of the precision matrix as the solution of a regularized and/or constrained optimization problem, to enforce sparsity of the solution. A popular approach is the graphical LASSO, first studied in [24, 25], that consists in maximizing the l_1-regularized log-likelihood. The analysis of the sample complexity of the graphical LASSO [26] gives an upper bound $O(\frac{\Delta^2}{\alpha^2\epsilon^2} \log m)$ sample complexity, where α is a parameter encoding a certain incoherence condition of the precision matrix. Other approaches are based on estimating the precision matrix one row at a time, via LASSO [27], Dantzig [28], or l_1-constrained optimization (CLIME) [29]. Simpler loss functions [30] and non-convex methods have also been considered [31]. These algorithms are computationally efficient, with runtimes polynomial with the number of variables. The sample complexity is $O(\log m)$, but only if appropriate assumptions, that can be broadly linked to the condition number of Θ being bounded, hold [32]. For the problem of graph selection, the paper [32] showed a sample complexity $O(\frac{\Delta \log m}{\kappa^2})$, with κ being a lower bound on the relative strengths of the interactions, and no condition number dependence, and matching the κ and m dependence in the lower bounds in [23]. The tradeoff is a worse scaling in computational complexity, still polynomial in m but with a degree depending on the degree of the graph, since for each node one needs to test all possible neighborhoods (improvements are possible for certain models in [33]). Our graph learning algorithm is inspired by the approach in [32] (especially their DICE algorithm). However, we were not able to remove the condition number dependence, and leave it to future work.

3 Supplementary note 3: Estimating the entries of the covariance matrix

In this section we will discuss the sample and computational complexity of estimating the covariance matrix of a Gaussian state. The estimates on the covariance matrix will be central to the other problems we consider in this paper, namely Hamiltonian and graph learning and trace distance learning. Our approach to estimate the entries of the covariance matrix is based on performing heterodyne measurements on the Gaussian state [2], which are experimentally feasible on the majority of platforms. The heterodyne measurement is a POVM constructed from the overcomplete set of coherent states, defined as:

$$|x\rangle := \mathcal{W}(x)\,|0\rangle\ , \tag{10}$$

where $|0\rangle$ is the vacuum state, with characteristic function

$$\chi_{|0\rangle\langle 0|}(u) = e^{-\frac{1}{4}\|u\|^2}\,.$$

It follows that the characteristic function of the coherent state $|x\rangle\langle x|$ takes the form

$$\chi_{|x\rangle\langle x|}(u) = e^{-\frac{1}{4}\|u\|^2+iu^\intercal x}\,.$$

From now on, for simplicity, we denote $V \equiv V[\rho]$ and $t \equiv t[\rho]$. Let us start by recalling the probability density of observing outcome $x \in \mathbb{R}^{2m}$ when measuring the Gaussian state ρ, which we denote by $\rho(x)$, and which evaluates to $\rho(x) = \frac{1}{(2\pi)^m}\operatorname{tr}[|x\rangle\langle x|\rho]$.

By Plancherel's theorem,

$$\begin{aligned}
\rho(x) &= \frac{1}{(2\pi)^{2m}}\int \chi_{|x\rangle\langle x|}(u)^*\chi_\rho(u)\,d^{2m}u\\
&= \frac{1}{(2\pi)^{2m}}\int e^{-\frac{1}{4}\|u\|^2-iu^\intercal x}e^{it^\intercal u-\frac{1}{2}u^\intercal Vu}\,d^{2m}u\\
&= \frac{1}{(2\pi)^{2m}}\int e^{-\frac{1}{2}u^\intercal(V+I/2)u+i(t-x)^\intercal u}d^{2m}u\\
&= \frac{1}{(2\pi)^m\det(V+I/2)^{\frac{1}{2}}}\,e^{-\frac{1}{2}(t-x)^\intercal(V+I/2)^{-1}(t-x)}\,,
\end{aligned}$$

where the last equation follows by direct Gaussian integration. In words, the heterodyne measurement of the state ρ generates a $2m$ dimensional Gaussian random vector $\mathbf{Z} := (Z_1,\ldots,Z_{2m})$ with mean t and covariance $\Sigma = V + I/2$:

$$t_i := \mathbb{E}[Z_i], \qquad (V+I/2)_{i,j} = \mathbb{E}[Z_iZ_j] - t_it_j\,.$$

We therefore have $2m+(2m)^2 = 2m(2m+1)$ coefficients which we can estimate by simple empirical averages: assuming access to N copies of the state ρ, after heterodyne measurement leading to the i.i.d. vectors $\mathbf{Z}^{(1)},\ldots,\mathbf{Z}^{(N)}$, we construct the estimators

$$\hat{t}_i := \frac{1}{N}\sum_{k=1}^N Z_i^{(k)} \qquad \hat{V}_{i,j}^{(0)} := \frac{1}{N}\sum_{k=1}^N Z_i^{(k)}Z_j^{(k)}\,.$$

By Gaussian concentration, we have that for any $i \in [2m]$

$$\mathbb{P}\big(|\hat{t}_i - t_i| \geq \epsilon\big) \leq 2e^{-\frac{N\epsilon^2}{2V_{i,i}+1}}\,.$$

Moreover, for any $i, j \in [2m]$, and $q \geq 1$,

$$\begin{aligned}\mathbb{E}\left[(Z_iZ_j)^{2q}\right] &\equiv \|Z_iZ_j\|_{2q}^{2q} \leq \|Z_i\|_{4q}^{2q}\ \|Z_j\|_{4q}^{2q} \\ &\leq (\|Z_i - t_i\|_{4q} + |t_i|)^{2q}\,(\|Z_j - t_j\|_{4q} + |t_j|)^{2q} \\ &\leq (2q)!\ \left[\left(\sqrt{2V_{i,i}+1} + |t_i|\right)\left(\sqrt{2V_{j,j}+1} + |t_j|\right)\right]^{2q},\end{aligned}$$

where we used that the centered $4q$-th moment of a Gaussian random variable with variance σ^2 is $\sigma^{4q}(4q-1)!!$. Denoting by $C[V,t] := \max_i 2\left(\sqrt{2V_{i,i}+1} + |t_i|\right)^2$, we have thus that, by triangle inequality and Jensen's inequality,

$$\mathbb{E}\left[\left(Z_iZ_j - \mathbb{E}\Big[Z_iZ_j\Big]\right)^{2q}\right] \leq 2^{2q-1}(\mathbb{E}\left[(Z_iZ_j)^{2q}\right] + (\mathbb{E}[Z_iZ_j])^{2q}) \leq C[V,t]^{2q}(2q)!\,.$$

and therefore the centered random variables $Z_iZ_j - \mathbb{E}\Big[Z_iZ_j\Big]$ are sub-Gamma with parameters $(4C[V,t]^2, 2C[V,t])$ by [34, Theorem 2.3]. This implies by Cramér-Chernoff method that, for $s < 1$,

$$\begin{aligned}\mathbb{P}\big(|\hat{V}^{(0)}_{i,j} - \mathbb{E}[Z_iZ_j]| \geq 2\sqrt{8C[V,t]^2 s}\big) &\leq \mathbb{P}\big(|\hat{V}^{(0)}_{i,j} - \mathbb{E}[Z_iZ_j]| \geq \sqrt{8C[V,t]^2 s} + 2C[V,t]s\big) \\ &\leq 2e^{-Ns}\,.\end{aligned}$$

Thus, for $\epsilon = 2\sqrt{8C[V,t]^2 s}$,

$$\mathbb{P}\big(|\hat{V}^{(0)}_{i,j} - \mathbb{E}[Z_iZ_j]| \geq \epsilon\big) \leq 2e^{-\frac{N\epsilon^2}{32C[V,t]^2}}\,.$$

Hence, by a union bound and using the fact that $C[V,t]^2 \geq \max_i 2V_{i,i} + 1$, for $\delta \in (0,1)$, and choosing $N \geq \frac{32C[V,t]^2}{\epsilon^2}\log\left(\frac{4m(2m+1)}{\delta}\right)$, we have that, with probability $1-\delta$,

$$|t_i - \hat{t}_i| \leq \epsilon, \qquad |\mathbb{E}[Z_iZ_j] - \hat{V}^{(0)}_{i,j}| \leq \epsilon\,, \qquad \forall i,j \in [2m]\,.$$

This further implies that, denoting

$$\hat{V}_{i,j} := \hat{V}^{(0)}_{i,j} - \hat{t}_i\hat{t}_j - \frac{\delta_{i,j}}{2}$$

we get with the same probability,

$$\left|\hat{V}_{i,j} - V_{i,j}\right| \leq \epsilon + \epsilon^2 + 2\epsilon\max_i |t_i|\,.$$

By rescaling the error probability, we directly get the following

Lemma 3.1. *In the notations of the previous paragraph, for any $\delta \in (0,1)$, $\epsilon \in (0,1/2)$ and $N \geq \frac{2^8 C[V,t]^2(1+\max_i|t_i|)^2}{\epsilon^2}\log\left(\frac{4m+1}{\delta}\right)$ with $C[V,t] := \max_i 2\left(\sqrt{2V_{i,i}+1} + |t_i|\right)^2$,*

$$|t_i - \hat{t}_i| \leq \epsilon, \qquad \textit{and} \qquad |\hat{V}_{i,j} - V_{i,j}| \leq \epsilon\,, \qquad \forall i,j \in [2m]\,. \tag{11}$$

We will later impose constraints on the set of Gaussian states we consider which will allow us to derive upper bounds on the value of $C[V,t]$.

In Lemma 3.1 we established the sample complexity of obtaining an empirical estimate of the covariance matrix such that each entry is close to the true covariance matrix with high probability. Note, however, that the matrix $\hat{V}$ will not necessarily be a valid covariance matrix, as this would require that $2\hat{V} \geq \pm i\Omega$. But, given an estimate $\hat{V}$ and a precision parameter ϵ, this issue can be readily fixed by solving the SDP:

$$\begin{aligned} &\max_{\tilde{V}\in\mathbb{R}^{2m\times 2m}} 1 \\ \forall i,j \in [2m] : -\epsilon \leq\ &\tilde{V}_{i,j} - \hat{V}_{i,j} \leq +\epsilon \\ &2\widetilde{V} \geq \pm i\Omega \\ &\widetilde{V} \geq 0 \end{aligned} \tag{12}$$

Note that conditioned on the estimates on $V_{i,j}$ being ϵ correct in the sense of Equation (11), the SDP in Equation (12) is feasible, as the true V is feasible. Furthermore, letting $\widetilde{V}$ be any feasible point of Equation (12), it readily follows from a triangle inequality that $|V_{i,j} - \widetilde{V}_{i,j}| \leq 2\epsilon$ for all $i,j \in [2m]$, and by the other constraints $\widetilde{V}$ is a valid covariance matrix. As SDPs can be solved in time polynomial in the dimension of the underlying matrices and the number of constraints, we conclude that:

Lemma 3.2. *In the setting of Lemma 3.1, for any $\delta \in (0,1)$, $\epsilon \in (0,1/2)$ and*

$$N = \mathcal{O}\left(\frac{C[V,t]^2(1+\max_i |t_i|)^2}{\epsilon^2}\log\left(\frac{4m+1}{\delta}\right)\right),$$

we can obtain a bona fide covariance matrix $\widetilde{V}$ satisfying

$$|\widetilde{V}_{i,j} - V_{i,j}| \leq 2\epsilon\,, \qquad \forall i,j \in [2m]\,. \tag{13}$$

in polynomial time in m by solving the SDP in Equation (12).

We will later (for Theorem 4.1) need similar results to convert empirical estimates of a Hamiltonian into a bona fide Hamiltonian. Recall that Hamiltonians H correspond to strictly positive symmetric matrices. However, $H > 0$ is not a semidefinite constraint and we will impose the stronger constraint $H \geq \tau I$ on H to make sure that finding a valid Hamiltonian from empirical estimates can be done efficiently. As we will discuss later in Sec. 9, given a lower bound $d_{\min}$ on the symplectic eigenvalues of H and the symplectic matrix S that diagonalizes H, we can take $0 \leq \tau \leq \|S\|^{-2} d_{\min}$ and H satisfies $H \geq \tau I$. Thus, given a (not necessarily positive) $\hat{H}$ satisfying:

$$\forall i,j \in [2m] : |\hat{H}_{i,j} - H_{i,j}| \leq \epsilon, \tag{14}$$

and a threshold $\tau > 0$, we can obtain a symmetric and positive $\widetilde{H}$ (and thus valid Hamiltonian) satisfying $|\tilde{H}_{i,j} - H_{i,j}| \leq 2\epsilon$ by solving the SDP:

$$\begin{aligned} &\max_{\widetilde{H}\in\mathbb{R}^{2m\times 2m}} 1 \\ \forall i,j \in [2m] : -\epsilon \leq\ &\widetilde{H}_{i,j} - \hat{H}_{i,j} \leq \epsilon \\ &\widetilde{H} \geq \tau I\,. \end{aligned} \tag{15}$$

Arguing as before, we conclude that this SDP can also be solved in time that is polynomial in the number of modes and logarithmic in the desired precision ϵ and threshold τ. Again, this is feasible as H itself is a solution. In addition, it is possible to only optimize over Hamiltonians that have the same underlying graph structure as H by imposing the additional linear constraints $\widetilde{H}_{2i-\delta,2j-\delta'} = 0$ for $(i,j) \notin \mathsf{E}$ and $\delta, \delta' \in \{0,1\}$.

Remark 3.1 (Robustness to measurement imperfections)**.** *Our analysis assumes ideal heterodyne measurements where the samples follow a distribution with covariance $V + I/2$. In realistic experiments, the dominant imperfections are finite detection efficiency $\eta \in (0,1]$ and electronic noise $v_{\mathrm{el}} \geq 0$ [35]. These are modeled by Gaussian channels, resulting in a measured covariance $\Sigma_{\mathrm{meas}} = \eta V + (1-\eta/2 + v_{\mathrm{el}})I$ [2]. Since the measurement noise is Gaussian, the samples remain multivariate normal, preserving the validity of the estimators derived above. These imperfections can be addressed in two ways. First, if η and v_{el} are known, one can construct an unbiased estimator for V by rescaling the empirical covariance, which increases the sample complexity by a constant factor η^{-2} but does not alter the scaling with respect to the system size m. Second, for small, uncharacterized noise, our protocols are robust with respect to Gaussian noise that changes the covariance matrix by an entry-wise error that is comparable with the admissible statistical error, which is independent of the system-size.*

Note also that our concentration bounds only used the sub-Gaussian and sub-Gamma properties of the estimators. If the implementation of the heterodyne measurement is not Gaussian (in fact, the standard implementation requires an infinite-energy local oscillator) but the estimators have expectation values sufficiently close to t and V, and the above sub-Gaussian and sub-Gamma properties are still satisfied, the conclusions of the analysis do not change.

4 Supplementary note 4: Learning in trace distance

In this section, we show how to learn an unknown Gaussian state in trace distance under certain constraints.

Definition 4.1 (Problem 1: Gaussian trace distance learning)**.** *Let $H \in M_{2m}^{>0}(\mathbb{R})$ be a symmetric, positive definite matrix and $t \in \mathbb{R}^{2m}$ (later referred to as Gaussian Hamiltonian). For any such data (H,t), one can construct a Gaussian state on m modes:*

$$\rho(H,t) = \frac{e^{-(R-t)^\intercal H(R-t)}}{\mathrm{tr}[e^{-(R-t)^\intercal H(R-t)}]}\,.$$

The Gaussian trace distance learning problem consists in the following task: given a fixed precision $\epsilon > 0$, provide estimates $\hat{H}$ of H and $\hat{t}$ of t such that $\|\rho(\hat{H},\hat{t}) - \rho(H,t)\|_1 \leq 2\epsilon$ with probability at least $1-\delta$, given n copies of $\rho(H,t)$, for any (H,t) in some specified subset $\mathcal{S}$. We denote by $\mathcal{N}_{\mathrm{TDL}}(\mathcal{S}, m, \epsilon, \delta)$ the sample complexity for Gaussian trace distance learning. This corresponds to the minimum number n of copies of the state $\rho(H,t)$ required for the task to succeed.

In this section we assume we have an estimate of the true covariance matrix and do not impose any graph structure for the underlying Hamiltonians (they could, e.g., be all-to-all connected). Importantly, we will obtain sample complexities that scale polynomially with the number of modes and quadratically with ϵ^{-1}, combined with polynomial postprocessing. This marks the first result on learning Gaussian states with such scaling [36], although unlike previous results, we need to impose

some constraints on the Gaussian states we consider in addition to a bounded energy constraint, which is in fact implied by our constraints.

We start with the standard observation that it is possible to bound the trace distance between thermal states in terms of the entries of V, t and H.

Proposition 4.1. *Given two Gaussian states ρ and $\hat{\rho}$, $\rho \equiv \rho(t, H)$, $W \equiv \mathcal{W}(-\Omega^{-1}t)$, $\hat{\rho} \equiv \rho(\hat{t}, \hat{H})$ and $\hat{W} \equiv \mathcal{W}(-\Omega^{-1}\hat{t})$, and denoting $T_{i,j} := t_i t_j + \hat{t}_i \hat{t}_j - \hat{t}_i t_j - t_i \hat{t}_j = (t_i - \hat{t}_i)(t_j - \hat{t}_j)$ we have*

$$\|\rho - \hat{\rho}\|_1 \leq \sqrt{\sum_{i,j} \left[(\hat{H}_{i,j} - H_{i,j})(V_{i,j} - \hat{V}_{i,j}) + T_{i,j}(H_{i,j} + \hat{H}_{i,j})\right]} \tag{16}$$

Proof. Using the symmetric relative entropy, we get from Pinsker's inequality that:

$$\begin{aligned}
\|\rho - \hat{\rho}\|_1 \leq \sqrt{D(\rho\|\hat{\rho}) + D(\hat{\rho}\|\rho)} &= \sqrt{\operatorname{tr}\left[(\rho - \hat{\rho})(\hat{W} R^{\intercal} \hat{H} R \hat{W}^{\dagger} - W R^{\intercal} H R W^{\dagger})\right]} \\
&= \sqrt{\sum_{i,j} \operatorname{tr}\left[(\rho - \hat{\rho})(\hat{H}_{i,j}(R_i - \hat{t}_i)(R_j - \hat{t}_j) - H_{i,j}(R_i - t_i)(R_j - t_j))\right]} \\
&= \sqrt{\sum_{i,j} \hat{H}_{i,j}\left(\frac{1}{2}\operatorname{tr}\left[\rho\{R_i - \hat{t}_i, R_j - \hat{t}_j\}\right] - \hat{V}_{i,j}\right) - \sum_{i,j} H_{i,j}\left(V_{i,j} - \operatorname{tr}\left[\hat{\rho}\frac{1}{2}\{R_i - t_i, R_j - t_j\}\right]\right)} \\
&= \sqrt{\sum_{i,j} \hat{H}_{i,j}\Big(V_{i,j} - \hat{V}_{i,j} + T_{i,j}\Big) - \sum_{i,j} H_{i,j}\Big(V_{i,j} - \hat{V}_{i,j} - T_{i,j}\Big)} \\
&= \sqrt{\sum_{i,j} \left[(\hat{H}_{i,j} - H_{i,j})(V_{i,j} - \hat{V}_{i,j}) + T_{i,j}(H_{i,j} + \hat{H}_{i,j})\right]}
\end{aligned}$$

where recall that $W R_i W^{\dagger} = R_i - t_i I$.

□

Clearly, if we can obtain estimates of the covariance matrix that are entrywise close, we can also bound the trace distance. Furthermore, it is to be expected that, as covariance matrices get closer, the corresponding Hamiltonians also converge. Such a continuity estimate is crucial to obtain an ϵ^{-2} in the sample complexity. One possible path to showing such a statement is discussed for spin systems in [37], where the authors obtain such statements in terms of the strong convexity properties of the partition function of states. We follow a different route and show several continuity bounds directly in Sec. 5. From these we obtain:

Theorem 4.1. *Let $H = \frac{1}{2}\log\left(\frac{2i\Omega V + I}{2i\Omega V - I}\right) i\Omega$ and $\hat{H} = \frac{1}{2}\log\left(\frac{2i\Omega\hat{V} + I}{2i\Omega\hat{V} - I}\right) i\Omega$ for two bona fide covariance matrices V, $\hat{V}$. We also denote by $d_{\max}$, resp. S, the maximal symplectic eigenvalue of H, resp. the symplectic matrix associated to H through Equation (58). Let ρ and $\hat{\rho}$ be Gaussian states with $\rho \equiv \rho(t, H)$, $W \equiv \mathcal{W}(-\Omega^{-1}t)$, $\hat{\rho} \equiv \rho(\hat{t}, \hat{H})$ and $\hat{W} \equiv \mathcal{W}(-\Omega^{-1}\hat{t})$. Given an approximation $\hat{V}$ of the covariance matrix V as well as an approximation $\hat{t}$ of the mean vector t to precision ϵ: $\max_{i,j}|V_{i,j} - \hat{V}_{i,j}| \leq \epsilon$, and $\max_i |t_i - \hat{t}_i| \leq \epsilon$, such that $2\|V - \hat{V}\|_\infty < \frac{e^{-2d_{\max}}}{1 - e^{-2d_{\max}}}\|S\|_\infty^{-2}$ we have*

$$\|\rho - \hat{\rho}\|_1 \leq m\epsilon\sqrt{16m(e^{2d_{\max}} - 1)^2\|S\|_\infty^4 + 8\|S\|_\infty^2 d_{\max}}\,. \tag{17}$$

Proof. Via Theorem 5.1, we have

$$\|H-\hat{H}\|_\infty \le 2\left(2\frac{e^{-2d_{\max}}}{1-e^{-2d_{\max}}}\|S\|_\infty^{-2}\right)^{-1}\left(2\frac{e^{-2d_{\max}}}{1-e^{-2d_{\max}}}\|S\|_\infty^{-2}-2\|V-\hat{V}\|_\infty\right)^{-1}\|V-\hat{V}\|_\infty\,,$$

which implies

$$\|H-\hat{H}\|_\infty \le 8m\left(2\frac{e^{-2d_{\max}}}{1-e^{-2d_{\max}}}\|S\|_\infty^{-2}\right)^{-2}\epsilon = 2m(e^{2d_{\max}}-1)^2\|S\|_\infty^4\epsilon\,.$$

Therefore, since $|H_{k,k'}-\hat{H}_{k,k'}|\le\|H-\hat{H}\|_\infty$, we have via Proposition 4.1 and its notation

$$\begin{aligned}\|\rho-\hat{\rho}\|_1 &\le \sqrt{8m^3(e^{2d_{\max}}-1)^2\|S\|_\infty^4\epsilon^2+8m^2\epsilon^2(\|S\|_\infty^2 d_{\max}+m(e^{2d_{\max}}-1)^2\|S\|_\infty^4\epsilon)}\\ &\le m\epsilon\sqrt{16m(e^{2d_{\max}}-1)^2\|S\|_\infty^4+8\|S\|_\infty^2 d_{\max}}\,.\end{aligned}$$

□

Combined with our results on estimating the covariance matrix in Sec. 3, this immediately yields:

Corollary 4.1. *Let $\rho(t,H)$ be a Gaussian state on m modes s.t. the largest symplectic eigenvalue $d_{\max}$ satisfies $d_{\max}\le\beta_{\max}$, the smallest symplectic eigenvalue $d_{\min}$ satisfies $d_{\min}\ge\beta_{\min}$, and $\|S\|_\infty\le s$ for known $s,\beta_{\max},\beta_{\min}>0$ and a known bound $\max_i|t_i|\le t_{\max}$. Then we have*

$$C[V,t]^2(1+\max_i|t_i|)^2 \le \max_i 16\left(2V_{i,i}+1+|t_i|^2\right)^2(1+\max_i|t_i|)^2 \tag{18}$$

$$= \mathcal{O}(s^4(1-e^{-2\beta_{\min}})^{-2}(1+\max_i|t_i|^6))\,, \tag{19}$$

and for $1>\epsilon,\delta>0$,

$$N = O(\epsilon^{-2}m^3s^8[\beta_{\max}+(e^{2\beta_{\max}}-1)^2](1-e^{-2\beta_{\min}})^{-2}(1+\max_i|t_i|^6)\log(m\delta^{-1})), \tag{20}$$

copies of ρ suffice to output an estimate of a bona fide covariance matrix $\hat{V}$, mean vector $\hat{t}$ and Hamiltonian $\hat{H}$ such that the corresponding Gaussian state is ϵ-close in trace distance to ρ with probability at least $1-\delta$. Furthermore, the postprocessing is polynomial in m. This implies $\mathcal{N}_{\mathrm{TDL}}(\mathcal{S},m,\epsilon,\delta)\le N$ for the class of states $\mathcal{S}$ satisfying the above conditions.

Proof. Equation (18) is a consequence of the bound on $\|V\|_\infty$ from Equation (60). Equation (20) follows from noting that Equation (17) implies that if the covariance matrix $\hat{V}$ satisfies $|V_{i,j}-\hat{V}_{i,j}|\le\epsilon'$ with $\epsilon'=\mathcal{O}(\epsilon m^{-1.5}(s^2\beta_{\max}+(e^{2\beta_{\max}}-1)^2s^4)^{-\frac{1}{2}})$ (which also implies that the condition $2\|V-\hat{V}\|_\infty<\frac{e^{-2d_{\max}}}{1-e^{-2d_{\max}}}\|S\|_\infty^{-2}$ is satisfied if $\epsilon<1$), then the two states are ϵ-close in trace distance. It then follows from Lemma 3.2 that we can obtain such a covariance matrix with the claimed sample complexity. The fact that the postprocessing is efficient follows from the fact that we just need to take the empirical averages and then solve an SDP to obtain the covariance matrix. If we wish to output the Hamiltonian, instead, we only need to perform an additional matrix inversion step, which is again polynomial in m. □

Remark 4.1. *Bounding V_{ii} by $\|V\|_\infty$ may be overshooting in the regime of large m. For example, for a covariance matrix with many entries of $\mathcal{O}(1)$, the norm can still be of $\Omega(m)$. The sample complexity can be as well expressed in terms of a bound on the diagonal elements of V.*

Remark 4.2. *The result above already illustrates the power of our methods, showing that learning Gaussian states in trace distance is possible with sample complexity scaling quadratically in precision. Thanks to Eq. (69), we can also write $C[V,t]^2(1+\max_i|t_i|)^2 = O(E^3)$ and using also Eq. (67) and Eq. (68) and following the steps in the proof above, we obtain that $N = O\left(\frac{m^3E^5(e^{2\beta_{\max}}-1)^2}{\epsilon^2}\log\frac{m}{\delta}\right)$ copies are sufficient for the task. Note that $\beta_{\max}$ and E are not independent, because $\beta_{\max} \geq \beta_{\min}$ and we used Eq. (68) to get rid of $\beta_{\min}$. Thus, $\beta_{\max} \to 0$ does not make the sample complexity going to zero.*

5 Supplementary note 5: Continuity bounds for Hamiltonians and covariances

In this section, we derive useful perturbation bounds for Gaussian Hamiltonians in terms of the norm difference between their corresponding covariance matrices, and vice versa. As we saw in the previous section, such bounds are essential when learning Gaussian states. We will use the following simple bound for the matrix inverse at multiple occasions:

$$\|A^{-1}-B^{-1}\|_\infty \leq \|A^{-1}\|_\infty\|B^{-1}\|_\infty\|A-B\|_\infty, \tag{21}$$

which readily follows from the identity $A^{-1}-B^{-1} = A^{-1}(B-A)B^{-1}$ and the submultiplicativity of the operator norm. We will also make use of the following integral representation of the matrix logarithm, which holds for A such that the spectrum $\mathrm{spec}(A)$ does not intersect with $(-\infty, 0]$:

$$\log(A) = (A-I)\int_0^\infty \frac{1}{tA+I}\frac{dt}{t+1}\,. \tag{22}$$

Lemma 5.1. *For $i\in\{1,2\}$, let $H_i = \frac{1}{2}\log\left(\frac{2i\Omega V_i+I}{2i\Omega V_i-I}\right)i\Omega$. We also denote by $d_{\max}$, resp. S, the maximal symplectic eigenvalue of H_1, resp. the symplectic matrix associated to H through Equation (58). Then, assuming that $\|V_1-V_2\|_\infty < \frac{e^{-2d_{\max}}}{1-e^{-2d_{\max}}}\|S\|_\infty^{-2}$,*

$$\|H_1-H_2\|_\infty \leq 2\left(2\frac{e^{-2d_{\max}}}{1-e^{-2d_{\max}}}\|S\|_\infty^{-2}\right)^{-1}\left(2\frac{e^{-2d_{\max}}}{1-e^{-2d_{\max}}}\|S\|_\infty^{-2} - 2\|V_1-V_2\|_\infty\right)^{-1}\|V_1-V_2\|_\infty,$$

Proof. Using (22), for $H\in\{H_1,H_2\}$, we have

$$\begin{aligned}
H &\equiv \frac{1}{2}\log\left(I+\frac{2}{2i\Omega V-I}\right)i\Omega \\
&= \left(\frac{1}{2i\Omega V-I}\int_0^\infty \frac{1}{t\frac{2i\Omega V+I}{2i\Omega V-I}+I}\frac{dt}{t+1}\right)i\Omega \\
&= \int_0^\infty \frac{1}{t(2i\Omega V+I)+(2i\Omega V-I)}\frac{dt}{t+1}i\Omega \\
&= \int_0^\infty \frac{1}{2i\Omega V+\frac{t-1}{t+1}I}\frac{dt}{(t+1)^2}i\Omega.
\end{aligned}$$

Then

$$\begin{aligned}
\|H_1 - H_2\|_\infty &= \left\| \int_0^\infty \frac{1}{2i\Omega V_1 + \frac{t-1}{t+1} I} \frac{dt}{(t+1)^2} - \int_0^\infty \frac{1}{2i\Omega V_2 + \frac{t-1}{t+1} I} \frac{dt}{(t+1)^2} \right\|_\infty \\
&\leq 2\|V_1 - V_2\|_\infty \int_0^\infty \left\| \left(2i\Omega V_2 + \frac{t-1}{t+1} I\right)^{-1} \right\|_\infty \left\| \left(2i\Omega V_1 + \frac{t-1}{t+1} I\right)^{-1} \right\|_\infty \frac{dt}{(t+1)^2} \\
&\leq \left(2 \frac{e^{-2d_{\max}}}{1-e^{-2d_{\max}}} \|S\|_\infty^{-2}\right)^{-1} \left(2 \frac{e^{-2d_{\max}}}{1-e^{-2d_{\max}}} \|S\|_\infty^{-2} - 2\|V_1 - V_2\|_\infty\right)^{-1} 2\|V_1 - V_2\|_\infty \,,
\end{aligned}$$

where the first inequality follows from (21), whereas the last one follows from (61) and the integral $\int \frac{dt}{(t+1)^2} = 1$. □

We also have the following bound.

Lemma 5.2. *For $i \in \{1, 2\}$, we let $K_i = \frac{2}{2i\Omega V_i - I}$. In the notations of the previous theorem, if $\|K_1 - K_2\|_\infty < e^{-2d_{\max}} \|S\|_\infty^{-2}$, then*

$$\|H_1 - H_2\|_\infty \leq \frac{1}{2} e^{2d_{\max}} \|S\|_\infty^2 \|K_1 - K_2\|_\infty \left(1 + \frac{e^{2d_{\max}} \|S\|_\infty^2 (1 - e^{-2d_{\max}}) + \|K_1 - K_2\|_\infty}{e^{-2d_{\max}} \|S\|_\infty^{-2} - \|K_1 - K_2\|_\infty}\right).$$

Proof. By writing $K_i = \frac{2}{2i\Omega V_i - I}$, we also have

$$H_i = \frac{1}{2} \log(I + K_i) i\Omega = \frac{1}{2} K_i \int_0^\infty \frac{1}{I + \frac{t}{t+1} K_i} \frac{dt}{(t+1)^2} i\Omega \,. \tag{23}$$

Let $\sigma_{\min}(A)$ be the smallest singular value of A. Since

$$\|K_1 - K_2\|_\infty < e^{-2d_{\max}} \|S\|_\infty^{-2} \leq \sigma_{\min}\left(\frac{t}{t+1} \frac{2i\Omega V + I}{2i\Omega V - I} + \frac{1}{t+1}\right)$$

from (62),

$$\begin{aligned}
H_2 &= \frac{1}{2} K_2 \int_0^\infty \frac{1}{I + \frac{t}{t+1} K_2} \frac{dt}{(t+1)^2} i\Omega \\
&= \frac{1}{2} K_2 \int_0^\infty \frac{1}{\frac{t}{t+1} \frac{2i\Omega V_1 + I}{2i\Omega V_1 - I} + \frac{1}{t+1} + \frac{t}{t+1}(K_2 - K_1)} \frac{dt}{(t+1)^2} i\Omega
\end{aligned}$$

is well defined and, by another use of (21),

$$\begin{aligned}
&\left\| \int_0^\infty \frac{1}{I + \frac{t}{t+1} K_1} \frac{dt}{(t+1)^2} - \int_0^\infty \frac{1}{I + \frac{t}{t+1} K_2} \frac{dt}{(t+1)^2} \right\|_\infty \\
&\leq \|K_1 - K_2\|_\infty \max_{t \geq 0} \left(\sigma_{\min}\left(\frac{t}{t+1} \frac{2i\Omega V_1 + I}{2i\Omega V_1 - I} + \frac{1}{t+1}\right) - \|K_1 - K_2\|_\infty\right)^{-1} \sigma_{\min}^{-1}\left(\frac{t}{t+1} \frac{2i\Omega V_1 + I}{2i\Omega V_1 - I} + \frac{1}{t+1}\right) \\
&\leq \|K_1 - K_2\|_\infty \left(e^{-2d_{\max}} \|S\|_\infty^{-2} - \|K_1 - K_2\|_\infty\right)^{-1} \left(e^{2d_{\max}} \|S\|_\infty^2\right),
\end{aligned}$$

where the last bound follows from (62). Finally, by a triangle inequality,

$$
\begin{aligned}
&\|H_1 - H_2\|_\infty = \frac{1}{2}\left\|K_1 \int_0^\infty \frac{1}{I + \frac{t}{t+1}K_1}\frac{dt}{(t+1)^2} - K_2\int_0^\infty \frac{1}{I+\frac{t}{t+1}K_2}\frac{dt}{(t+1)^2}\right\|_\infty \\
&\leq \frac{1}{2}\|K_1 - K_2\|_\infty e^{2d_{\max}}\|S\|_\infty^2\left(1 + \|K_2\|_\infty\left(e^{-2d_{\max}}\|S\|_\infty^{-2} - \|K_1-K_2\|_\infty\right)^{-1}\right) \\
&\leq \frac{1}{2}\|K_1 - K_2\|_\infty e^{2d_{\max}}\|S\|_\infty^2\left(1 + \left(\|K_2 - K_1\|_\infty + \|K_1\|_\infty\right)\left(e^{-2d_{\max}}\|S\|_\infty^{-2} - \|K_1-K_2\|_\infty\right)^{-1}\right) \\
&\leq \frac{1}{2}e^{2d_{\max}}\|S\|_\infty^2\|K_1-K_2\|_\infty\left(1 + \frac{e^{2d_{\max}}\|S\|_\infty^2(1-e^{-2d_{\max}}) + \|K_1-K_2\|_\infty}{e^{-2d_{\max}}\|S\|_\infty^{-2} - \|K_1-K_2\|_\infty}\right).
\end{aligned}
$$

where we used Equation (61) in the last line in order to bound $\|K_1\|_\infty$. □

We now prove a continuity bound for covariance matrices in terms of their associated Gaussian Hamiltonians.

Lemma 5.3. *Let H_1 and H_2 be two Hamiltonian matrices, H_1 with normal form $H_1 = S^{-\intercal}DS^{-1}$, and $\|H_1 - H_2\|_\infty \leq \frac{1}{4\|S\|_\infty^6}\left(e^{-2d_{\max}-1}(1-e^{-2d_{\min}})\right)$. Then*

$$
\|V_1 - V_2\|_\infty \leq 4\,\frac{\|S\|_\infty^8 e^{2d_{\max}+1}\|H_1 - H_2\|_\infty}{(1-e^{-2d_{\min}})^2}\,. \tag{24}
$$

Proof. First of all, notice that $\|(2i\Omega V_1 - I)^{-1} - (2i\Omega V_2 - I)^{-1}\|_\infty = \frac{1}{2}\|e^{2H_1 i\Omega} - e^{2H_2 i\Omega}\|_\infty$ by (7). Moreover, for $s,t\in[0,1]$,

$$
\begin{aligned}
\left\|e^{2s(tH_1+(1-t)H_2)i\Omega}\right\|_\infty &= \left\|e^{2s(H_1 i\Omega + (1-t)(H_2-H_1)i\Omega)}\right\|_\infty \\
&= \left\|S^{-\intercal}e^{2s(D_1 i\Omega + (1-t)S^\intercal(H_2-H_1)Si\Omega)}S^\intercal\right\|_\infty \\
&\leq \|S\|_\infty^2 e^{2s\|D_1 i\Omega + (1-t)S^\intercal(H_2-H_1)Si\Omega\|_\infty} \\
&\leq \|S\|_\infty^2 e^{2sd_{\max}+2s\|S\|_\infty^2\|H_2-H_1\|_\infty}\,.
\end{aligned} \tag{25}
$$

Hence, by Duhamel formula,

$$
e^{2H_1 i\Omega} - e^{2H_2 i\Omega} = \int_0^1\int_0^1 e^{2s(tH_1+(1-t)H_2)i\Omega}2(H_1-H_2)i\Omega e^{2(1-s)(tH_1+(1-t)H_2)i\Omega}dt\,ds\,, \tag{26}
$$

we can estimate

$$
\begin{aligned}
\left\|e^{2H_1 i\Omega} - e^{2H_2 i\Omega}\right\|_\infty &\leq 2\int_0^1\int_0^1\left\|e^{2s(tH_1+(1-t)H_2)i\Omega}\right\|_\infty\|H_1-H_2\|_\infty\left\|e^{2(1-s)(tH_1+(1-t)H_2)i\Omega}\right\|_\infty dt\,ds \\
&\leq 2\|S\|_\infty^4 e^{2d_{\max}+2\|S\|_\infty^2\|H_2-H_1\|_\infty}\|H_1-H_2\|_\infty\,.
\end{aligned} \tag{27}
$$

We then have, by perturbation bounds on the inverse (21) and the condition in the theorem statement,

$$\begin{aligned}
\|V_1 - V_2\|_\infty &= \frac{1}{2}\|2i\Omega V_1 - I - (2i\Omega V_2 - I)\|_\infty \\
&\leq \frac{1}{2}\|2i\Omega V_1 - I\|_\infty \|2i\Omega V_2 - I\|_\infty \|(2i\Omega V_1 - I)^{-1} - (2i\Omega V_2 - I)^{-1}\|_\infty \\
&\leq \frac{1}{4}\frac{2\|S\|_\infty^2}{1-e^{-2d_{\min}}}\left[\left(\|S\|_\infty^2 \frac{2}{1-e^{-2d_{\min}}}\right)^{-1} - \frac{1}{2}\|e^{2H_1 i\Omega} - e^{2H_2 i\Omega}\|_\infty\right]^{-1} \|e^{2H_1 i\Omega} - e^{2H_2 i\Omega}\|_\infty \\
&\leq \frac{\|S\|_\infty^4}{(1-e^{-2d_{\min}})^2}\left[1 - \frac{\|S\|_\infty^2}{1-e^{-2d_{\min}}}\|e^{2H_1 i\Omega} - e^{2H_2 i\Omega}\|_\infty\right]^{-1} \|e^{2H_1 i\Omega} - e^{2H_2 i\Omega}\|_\infty \\
&\leq \frac{2\|S\|_\infty^8 e^{2d_{\max}+2\|S\|_\infty^2\|H_2-H_1\|_\infty}\|H_1 - H_2\|_\infty}{(1-e^{-2d_{\min}})^2\left[1 - \frac{2\|S\|_\infty^6 e^{2d_{\max}+2\|S\|_\infty^2\|H_2-H_1\|_\infty}}{1-e^{-2d_{\min}}}\|H_1 - H_2\|_\infty\right]} \\
&\leq \frac{2\|S\|_\infty^8 e^{2d_{\max}+2\|S\|_\infty^2\|H_2-H_1\|_\infty}\|H_1 - H_2\|_\infty}{(1-e^{-2d_{\min}})^2\left[1 - \frac{2\|S\|_\infty^6 e^{2d_{\max}+1}}{1-e^{-2d_{\min}}}\|H_1 - H_2\|_\infty\right]} \\
&\leq 4\frac{\|S\|_\infty^8 e^{2d_{\max}+1}\|H_1 - H_2\|_\infty}{(1-e^{-2d_{\min}})^2},
\end{aligned}$$

where we also used (60) in the second inequality above, and where in the last two inequalities we also used that $\|H_1 - H_2\|_\infty \leq (2\|S\|_\infty^2)^{-1}$. □

6 Supplementary note 6: Sparse approximations of $(2V - i\Omega)^{-1}$

The main goal of this section is to derive a sparse approximation of $(2V - i\Omega)^{-1}$ from an estimated covariance matrix $\hat{V}$. We start by stating a simple Taylor expansion bound for the matrix $(2V - i\Omega)^{-1}$ which will turn out to be crucial for our later local perturbation analysis. Indeed, one of the main ideas of our work is to invert the matrix $2V - i\Omega$ only on submatrices and show that this still yields good estimates of the Hamiltonian if we follow this procedure on appropriate submatrices. This way we can make sure that the precision we need to estimate the covariance matrix to estimate the terms of the Hamiltonian scales logarithmically with the number of modes.

Lemma 6.1. *Let V be a covariance matrix of a Gaussian state with Hamiltonian $H = S^{-\intercal}DS^{-1}$. We have*

$$(2V - i\Omega)^{-1} = -\frac{I - e^{+2Hi\Omega}}{2}(i\Omega) = \frac{1}{2}\sum_{n=1}^{\infty}\frac{(2Hi\Omega)^n}{n!}(i\Omega) = \frac{1}{2}\sum_{n=1}^{l}\frac{(2Hi\Omega)^n}{n!}(i\Omega) + E\,, \tag{28}$$

with $\|E\|_\infty \leq \frac{\|S\|_\infty^2}{2}\left(\frac{(2d_{\max})^{l+1}e^{2d_{\max}}}{(l+1)!}\right)$.

Proof. We recall that $V = \frac{1}{2}i\Omega\coth(Hi\Omega)$ by (7), so $2i\Omega V - I = \coth(Hi\Omega) - I$. Then, (28) comes from writing $\coth(x) - 1 = \frac{2e^{-x}}{e^x - e^{-x}} = -\frac{2}{1-e^{2x}}$ and using the series expansion of the exponential

function. The bound on the remainder term E in (28) arises from the identity

$$E(i\Omega) = S^{-\intercal}\left(\frac{1}{2}\sum_{n=l+1}^{\infty}\frac{(2Di\Omega)^n}{n!}\right)S^{\intercal},$$

and thus

$$\begin{aligned}
EE^{\dagger} &= S^{-\intercal}\left(\frac{1}{2}\sum_{n=l+1}^{\infty}\frac{(2Di\Omega)^n}{n!}\right)S^{\intercal}S\left(\frac{1}{2}\sum_{n=l+1}^{\infty}\frac{(2Di\Omega)^n}{n!}\right)S^{-1} \\
&\leq \sigma_{\max}(S^{\intercal}S)S^{-\intercal}\left(\frac{1}{2}\sum_{n=l+1}^{\infty}\frac{(2Di\Omega)^n}{n!}\right)^2 S^{-1} \\
&\leq \frac{\sigma_{\max}(S^{\intercal}S)}{4}\left(\frac{(2d_{\max})^{l+1}e^{2d_{\max}}}{(l+1)!}\right)^2 S^{-\intercal}S^{-1} \\
&\leq \frac{\sigma^2_{\max}(S^{\intercal}S)}{4}\left(\frac{(2d_{\max})^{l+1}e^{2d_{\max}}}{(l+1)!}\right)^2
\end{aligned}$$

where in the second inequality we estimated the Lagrange remainder. Thus we obtain $\|E\|_\infty \leq \frac{\sigma_{\max}(SS^{\intercal})}{2}\left(\frac{(2d_{\max})^{l+1}e^{2d_{\max}}}{(l+1)!}\right)$. □

Remark 6.1. *Assuming that H has an underlying graph structure, note that $((2V - i\Omega)^{-1} - E)_{2i-\delta_1,2j-\delta_2}$, with $\delta_1, \delta_2 \in 0, 1$, is zero if j is at distance more than l from i. This property will be crucial in the following.*

Lemma 6.2. *Let $M = \left(\begin{smallmatrix} A & B \\ C & D\end{smallmatrix}\right) \in \mathbb{C}^{(m_1+m_2)\times(m_1+m_2)}$ be a positive definite matrix with blocks $A \in \mathbb{C}^{m_1\times m_1}$, $B \in \mathbb{C}^{m_1\times m_2}$, $C \in \mathbb{C}^{m_2\times m_1}$, $D \in \mathbb{C}^{m_2\times m_2}$, and such that D and $A - BD^{-1}C$ are invertible. Let $N \in \mathbb{C}^{m_1\times m_1}$ be the upper-left block of M^{-1}. Denote by $\widetilde{B}$, resp. $\widetilde{C}$, the matrix obtained from B by substituting the first row with zeros, resp. from C by substituting the first column with zeros. Clearly $B = C^{\dagger}$, $\widetilde{B} = \widetilde{C}^{\dagger}$ and $\|B - \widetilde{B}\|_\infty = \|C - \widetilde{C}\|_\infty$. Moreover,*

$$\begin{aligned}
|(N^{-1})_{1,j} - A_{1,j}| &\leq \|B - \widetilde{B}\|_\infty \|M^{-1}\|_\infty \|M\|_\infty \ \forall j \in m_1, \\
|(N^{-1})_{j,1} - A_{j,1}| &\leq \|B - \widetilde{B}\|_\infty \|M^{-1}\|_\infty \|M\|_\infty \ \forall j \in m_1.
\end{aligned}$$

Proof. We have by block-inversion that

$$M^{-1} = \begin{pmatrix} N \equiv (A - BD^{-1}C)^{-1} & -(A - BD^{-1}C)^{-1}BD^{-1} \\ -D^{-1}C(A - BD^{-1}C)^{-1} & D^{-1} + D^{-1}C(A - BD^{-1}C)^{-1}BD^{-1}\end{pmatrix}.$$

By definition, $A - BD^{-1}C = N^{-1}$. Since by construction the first row of $\widetilde{B}$ is identically zero, $\widetilde{B}_{1,j} = 0\ \forall j \in [m_2]$. Similarly, $\widetilde{C}_{j,1} = 0\ \forall j \in [m_2]$. Then $(\widetilde{B}D^{-1}C)_{1,j} = 0\ \forall j \in [m_1]$ and $(BD^{-1}\widetilde{C})_{j,1} = 0\ \forall j \in [m_1]$. This means that $(A - \widetilde{B}D^{-1}C)_{1,j} = A_{1,j}\ \forall j \in [m_1]$ and $(A - BD^{-1}\widetilde{C})_{j,1} = A_{j,1}\ \forall j \in [m_1]$. Furthermore, $\|A - BD^{-1}C - (A - \widetilde{B}D^{-1}C)\|_\infty \leq \|B - \widetilde{B}\|_\infty\|D^{-1}\|_\infty\|C\|_\infty$. Moreover, $\|C\|_\infty \leq \|M\|_\infty$ and $\sigma_{\min}(D) \geq \sigma_{\min}(M)$ as the matrix M is positive definite, and hence $\|D^{-1}\|_\infty \leq \|M^{-1}\|_\infty$. Then $\|A - BD^{-1}C - (A - \widetilde{B}D^{-1}C)\|_\infty \leq \|B - \widetilde{B}\|_\infty\|M^{-1}\|_\infty\|M\|_\infty$. In the same way, $\|A - BD^{-1}C - (A - BD^{-1}\widetilde{C})\|_\infty \leq \|C - \widetilde{C}\|_\infty\|M^{-1}\|_\infty\|M\|_\infty$. The bounds in the statement follow. □

Remark 6.2. *$\|M\|_\infty \|M^{-1}\|_\infty$ is the condition number of M. Looking at the proof, it is clear that instead of $\|M\|_\infty \|M^{-1}\|_\infty$ we can leave $\|D^{-1}C\|_\infty$ in the bound statement. When C contains very few columns with respect to the size of D, and D has a large condition number, it would be natural to guess that $\|D^{-1}C\|_\infty$ does not scale as $\|D^{-1}\|_\infty$, as the subspace spanned by the column space of C may not be aligned with the eigenspaces with largest eigenvalues of D^{-1}. All the following bounds can be in principle improved using this fact. We prefer not to do it explicitly since a bound on $\|D^{-1}C\|_\infty$ is not a natural assumption in our context.*

We will now introduce some definitions and notations necessary to explain our results on the local inversion technique.

Definition 6.1 (Neighborhood structure)**.** *A collection $\mathcal{N} = \{\mathcal{N}_i\}_{i\in[m]}$ of subsets of $[m]$ is called a neighborhood structure if for all $i \in [m]$, $i \in \mathcal{N}_i$ and for all $i, j \in [m]$, $i \in \mathcal{N}_j \Rightarrow j \in \mathcal{N}_i$.*

Remark 6.3. *We use the term neighborhood to follow the convention used in classical papers on learning graphical models. But we emphasize that the $\mathcal{N}_i$ will* not *be the neighborhood of a vertex i of the interaction graph. Rather, the $\mathcal{N}_i$ will often correspond to candidates for the set of all vertices at most a certain distance away from i.*

Definition 6.2 (Localization)**.** *Given a $2m \times 2m$ self-adjoint matrix M and a neighborhood structure $\mathcal{N} = \{\mathcal{N}_i\}_{i\in[m]}$, let $\mathrm{LOC}_{\mathcal{N}}(M)$ be the matrix obtained from M by keeping the matrix elements given by the neighborhood structure $\mathcal{N}$, that is, we define $\mathrm{LOC}_{\mathcal{N}}(M)_{2i-\delta_1,2j-\delta_2} = M_{2i-\delta_1,2j-\delta_2}$ whenever $j \in \mathcal{N}_i$ and $\delta_1, \delta_2 \in \{0, 1\}$, and set all other entries to 0. Note that, by construction, $\mathrm{LOC}_{\mathcal{N}}(M)$ is self-adjoint and has at most $2\max_i |\mathcal{N}_i|$ non-zero element per row.*

Given a neighborhood structure, we will construct approximate inverses of matrices by "stitching together" the inverses of various submatrices with only entries in the neighborhood structure. More formally, we have:

Definition 6.3 (Local inversion)**.** *Let N be an Hermitian $2m \times 2m$ matrix and let $\mathcal{N} = \{\mathcal{N}_i\}_{i\in[m]}$ be a neighborhood structure of $[m]$. We denote by $\mathrm{LI}_{\mathcal{N}}(N)$ the matrix constructed in the following way: for each $i \in [m]$, let N_i be the $2|\mathcal{N}_i| \times 2|\mathcal{N}_i|$ matrix (assumed to be invertible) with entries $N_{2j_1-\delta_1,2j_2-\delta_2}$ corresponding to indices $j_1, j_2 \in \mathcal{N}_i$, $\delta, \delta' \in \{0, 1\}$ (i.e. we select modes in $\mathcal{N}_i$ and we retain the corresponding labels for the matrix entries). Then, for each $i \in [m]$, $j \in \mathcal{N}_i$ and $\delta, \delta' \in \{0, 1\}$, we define $(\mathrm{LI}_{\mathcal{N}_i}(N))_{2i-\delta,2j-\delta'}$ to be $\left(N_i^{-1}\right)_{2i-\delta,2j-\delta'}$ and $(\mathrm{LI}_{\mathcal{N}}(N))_{2i-\delta,2j-\delta'}$ to be equal to $\frac{1}{2}\left[\left(N_i^{-1}\right)_{2i-\delta,2j-\delta'} + \left(N_j^{-1}\right)^*_{2j-\delta',2i-\delta}\right]$, and set the other entries of $\mathrm{LI}_{\mathcal{N}}(N)$ to 0. Note that, by construction, $\mathrm{LI}_{\mathcal{N}}(N)$ is self-adjoint, as both $\left(N_i^{-1}\right)$ and $\left(N_j^{-1}\right)$ are.*

We then have the following first lemma bounding the difference between the local inverse and the true inverse:

Lemma 6.3. *Let M be a positive definite matrix and let $\mathcal{N} = \{\mathcal{N}_i\}_{i\in[m]}$ be a neighborhood structure of $[m]$. For each $i \in [m]$ and $\delta \in \{0, 1\}$, let $\begin{pmatrix} A_{i,\delta} & B_{i,\delta} \\ C_{i,\delta} & D_{i,\delta} \end{pmatrix} \in \mathbb{C}^{2m\times 2m}$, with blocks $A_{i,\delta} \in \mathbb{C}^{2|\mathcal{N}_i|\times 2|\mathcal{N}_i|}, B_{i,\delta} \in \mathbb{C}^{2|\mathcal{N}_i|\times 2m-2|\mathcal{N}_i|}, C_{i,\delta} \in \mathbb{C}^{2m-2|\mathcal{N}_i|\times 2|\mathcal{N}_i|}, D_{i,\delta} \in \mathbb{C}^{2m-2|\mathcal{N}_i|\times 2m-2|\mathcal{N}_i|}$ be the matrix $\pi_{i,\delta} M \pi_{i,\delta}^{-1}$ where $\pi_{i,\delta}$ is a permutation that brings the coefficients $2j - \delta'$ with $\delta' \in \{0, 1\}$ and $j \in \mathcal{N}_i$ to the upper-left block $A_{i,\delta}$, in such a way that index $2i - \delta$ is mapped to 1. Let $\widetilde{B}_{i,\delta}$ be the*

matrix constructed from $B_{i,\delta}$ by putting the first row to zero. Let E be a self-adjoint matrix such that $\mathrm{LOC}_{\mathcal{N}}(M-E)=M-E$. *Then,*

$$\|M-\mathrm{LI}_{\mathcal{N}}(M^{-1})\|_\infty \le 2\max_{i\in[m]}\max_{\delta\in\{0,1\}}|\mathcal{N}_i|\|B_{i,\delta}-\widetilde{B}_{i,\delta}\|_\infty\|M^{-1}\|_\infty\|M\|_\infty+(2\max_{i\in[m]}|\mathcal{N}_i|+1)\|E\|_\infty \quad (29)$$

Proof. By a triangle inequality, we have

$$\|M-\mathrm{LI}_{\mathcal{N}}(M^{-1})\|_\infty \le \|E\|_\infty + \|M-E-\mathrm{LI}_{\mathcal{N}}(M^{-1})\|_\infty\,.$$

It remains to bound the last operator norm above. Since $\mathrm{LOC}_{\mathcal{N}}(M-E)-\mathrm{LI}_{\mathcal{N}}(M^{-1})=M-E-\mathrm{LI}_{\mathcal{N}}(M^{-1})$ is self-adjoint, it suffices to find a uniform bound on its eigenvalues. By the Gershgorin circle theorem, denoting the matrix $L:=\mathrm{LOC}_{\mathcal{N}}(M-E)-\mathrm{LI}_{\mathcal{N}}(M^{-1})$, these eigenvalues are situated in the $2m$ intervals

$$\left[L_{k,k}-\sum_{k'\neq k}\left|L_{k,k'}\right|,\, L_{k,k}+\sum_{k'\neq k}\left|L_{k,k'}\right|\right],\ k\in[2m]\,.$$

Now for any $i\in[m]$, $j\in\mathcal{N}_i$ and $\delta'\in\{0,1\}$, we have by the construction of the local inverse matrix, denoting $N=M^{-1}$:

$$\begin{aligned}\left|M_{2i-\delta,2j-\delta'}-\left(\mathrm{LI}_{\mathcal{N}}(M^{-1})\right)_{2i-\delta,2j-\delta'}\right| &\le \frac{1}{2}\left|M_{2i-\delta,2j-\delta'}-\left(N_i^{-1}\right)_{2i-\delta,2j-\delta'}\right| \\ &+\frac{1}{2}\left|M_{2i-\delta,2j-\delta'}-\left(N_j^{-1}\right)^*_{2j-\delta',2i-\delta}\right|.\end{aligned}$$

Via Lemma 6.2 applied to the matrices $\pi_{i,\delta}M\pi_{i,\delta}^{-1}$ and $\pi_{j,\delta'}M\pi_{j,\delta'}^{-1}$,

$$\begin{aligned}&\left|M_{2i-\delta,2j-\delta'}-\left(N_i^{-1}\right)_{2i-\delta,2j-\delta'}\right| \le \|B_{i,\delta}-\widetilde{B}_{i,\delta}\|_\infty\|M^{-1}\|_\infty\|M\|_\infty\,,\\ &\left|M_{2i-\delta,2j-\delta'}-\left(N_j^{-1}\right)^*_{2j-\delta',2i-\delta}\right| = \left|M^*_{2j-\delta',2i-\delta}-\left(N_j^{-1}\right)^*_{2j-\delta',2i-\delta}\right| \le \|B_{j,\delta'}-\widetilde{B}_{j,\delta'}\|_\infty\|M^{-1}\|_\infty\|M\|_\infty\end{aligned}$$

and hence $|L_{2i-\delta,2j-\delta'}|\le|(M-\left(\mathrm{LI}_{\mathcal{N}}(M^{-1})\right)_{2i-\delta,2j-\delta'}|+|E_{2i-\delta,2j-\delta'}|\le\max_{i\in[m]}\max_{\delta\in\{0,1\}}\|B_{i,\delta}-\widetilde{B}_{i,\delta}\|_\infty\|M^{-1}\|_\infty\|M\|_\infty+\|E\|_\infty$. Using Gershgorin's theorem together with the fact that the matrix L has at most $2\max_i|\mathcal{N}_i|$ non-zero coefficients per row, we get

$$\|L\|_\infty \le 2\max_{i\in[m]}\max_{\delta\in\{0,1\}}|\mathcal{N}_i|\left(\|B_{i,\delta}-\widetilde{B}_{i,\delta}\|_\infty\|M^{-1}\|_\infty\|M\|_\infty+\|E\|_\infty\right)\,.$$

The result follows.

□

However, in our setting we will only have access to approximations of the true covariance matrix when performing the local inversions. Assuming the accuracy of the estimate is good enough, the N_i matrices are in fact positive-definite, and the local inversion is well-defined. In the next lemma we bound the effect caused by this, i.e. when we only have access to an entrywise approximation of the inverse of a matrix:

Lemma 6.4. *In the notations of Lemma 6.3, given a $2m \times 2m$ positive definite matrix M, $N = M^{-1}$ and $\hat{N}$ an entrywise approximation of N, where $|\hat{N}_{k,k'} - N_{k,k'}| \leq \zeta$ for all $k, k' \in [2m]$ with $\zeta \leq \frac{1}{4\max_{i\in[m]}|\mathcal{N}_i|}\|M\|_\infty^{-1}$,*

$$\begin{aligned}\|M - \mathrm{LI}_{\mathcal{N}}(\hat{N})\|_\infty \leq 2\max_{i\in[m]}\max_{\delta\in\{0,1\}}|\mathcal{N}_i|\|B_{i,\delta} - \widetilde{B}_{i,\delta}\|_\infty\|M^{-1}\|_\infty\|M\|_\infty + (2\max_{i\in[m]}|\mathcal{N}_i| + 1)\|E\|_\infty \\ + 8\|M\|_\infty^2\max_i|\mathcal{N}_i|^2\zeta\,.\end{aligned}$$

Proof. By the triangle inequality,

$$\|M - \mathrm{LI}_{\mathcal{N}}(\hat{N})\|_\infty \leq \|M - \mathrm{LI}_{\mathcal{N}}(N)\|_\infty + \|\mathrm{LI}_{\mathcal{N}}(N) - \mathrm{LI}_{\mathcal{N}}(\hat{N})\|_\infty.$$

The first term can be bounded as in Lemma 6.3. The second term can be bounded using the fact that both $\mathrm{LI}_{\mathcal{N}}(N)$ and $\mathrm{LI}_{\mathcal{N}}(\hat{N})$ have at most $2\max_{i\in[m]}|\mathcal{N}_i|$ non-zero elements on each column, and each non-zero entry can be bounded using the perturbation bound for the inverse (cf. Equation (21)): for each $i \in [m]$ $j \in \mathcal{N}_i$, $\delta_1, \delta_2 \in \{0,1\}$, we have by the equivalence between the max norm and operator norm of matrices:

$$\begin{aligned}\left|\left(\mathrm{LI}_{\mathcal{N}}(\hat{N})\right)_{2i-\delta_1,2j-\delta_2} - \left(\mathrm{LI}_{\mathcal{N}}(N)\right)_{2i-\delta_1,2j-\delta_2}\right| &\leq \frac{1}{2}\left(\|N_i^{-1} - (\hat{N}_i)^{-1}\|_\infty + \|N_j^{-1} - (\hat{N}_j)^{-1}\|_\infty\right) \\ &\leq \max_{i\in[m]}\|N_i^{-1}\|_\infty\|(\hat{N}_i)^{-1}\|_\infty\|N_i - \hat{N}_i\|_\infty \\ &\leq \max_{i\in[m]}\|N_i^{-1}\|_\infty\|(\hat{N}_i)^{-1}\|_\infty\|N_i - \hat{N}_i\|_\infty \\ &\leq \max_{i\in[m]}\sigma_{\min}(N_i)^{-1}\sigma_{\min}(\hat{N}_i)^{-1}2|\mathcal{N}_i|\zeta \\ &\leq \max_{i\in[m]}\sigma_{\min}(M^{-1})^{-1}(\sigma_{\min}(N_i) - \|N_i - \hat{N}_i\|_\infty)^{-1}2|\mathcal{N}_i|\zeta \\ &\leq \max_{i\in[m]}\|M\|_\infty(\|M\|_\infty^{-1} - 2|\mathcal{N}_i|\zeta)^{-1}2|\mathcal{N}_i|\zeta \\ &\leq \max_{i\in[m]}4\|M\|_\infty^2|\mathcal{N}_i|\zeta\,, \end{aligned} \tag{30}$$

where we used that $\|N_i - \hat{N}_i\|_\infty \leq \|N_i - \hat{N}_i\|_2 \leq 2|\mathcal{N}_i|\zeta$, $\sigma_{\min}(N_i) \geq \sigma_{\min}(M^{-1})$, and the condition on ζ in the theorem statement. The bound on $\|\mathrm{LI}_{\mathcal{N}}(N) - \mathrm{LI}_{\mathcal{N}}(\hat{N})\|_\infty$ follows again from the use of Gershgorin's theorem. □

We now apply these perturbation bounds to the estimation of $2i\Omega V - I = i\Omega(2V - i\Omega)$. First we recall the following definition

Definition 6.4 (Interaction graph)**.** *Given a Hamiltonian matrix H, its interaction graph is a graph $G = ([m], \mathsf{E})$ made of m vertices, where two vertices distinct vertices $i, j \in [m]$ are connected by an edge whenever the 2×2 sub-matrix $\left\{H_{2i-\delta,2j-\delta'}\right\}_{\delta,\delta'\in\{0,1\}}$ is non-zero.*

We also define a neighborhood structure that will play an important role in our proofs.

Definition 6.5 (l-neighborhoods)**.** *Given $l \in \mathbb{N}$ and a Hamiltonian matrix H, let G be the interaction graph of H. The l-neighborhood of a site $i \in [m]$ is the set of indices j in $[m]$ that are at distance at most l from i, according to the graph G. We denote the set of l-neighborhoods as $\mathcal{N}(l) = \{\mathcal{N}(l)_i\}_{i\in[m]}$ and $\xi(l) := \max_{i\in[m]}|\mathcal{N}(l)_i|$. Clearly, any set of l-neighborhood satisfies the definition 6.1 of a neighborhood structure.*

Lemma 6.5. *Let V be a bona fide m-mode covariance matrix with associated symplectic matrix S given by Equation (59). For $l \geq 1$, let $\zeta < \frac{1}{2(\xi(l))} \frac{e^{-2d_{\max}}}{\|S\|_\infty^2 (1-e^{-2d_{\max}})}$ and a matrix $\hat{V}$ such that $|2\hat{V}_{k,k'} - 2V_{k,k'}| \leq \zeta$ for and any $k, k' \in [2m]$. Then, $\mathrm{LI}_{\mathcal{N}(l)}(2\hat{V} - i\Omega)$ is well defined, and*

$$\begin{aligned}
&\|(2V - i\Omega)^{-1} - \mathrm{LI}_{\mathcal{N}(l)}(2\hat{V} - i\Omega)\|_\infty \\
&\quad \leq \xi(l)\|S\|_\infty^6 \frac{(2d_{\max})^{l+1} e^{4d_{\max}}}{(l+1)!} \frac{1 - e^{-2d_{\max}}}{1 - e^{-2d_{\min}}} + (2\xi(l)+1) \frac{\|S\|_\infty^2}{2} \frac{(2d_{\max})^{l+1} e^{2d_{\max}}}{(l+1)!} \\
&+ 2\|S\|_\infty^4 e^{4d_{\max}} (1 - e^{-2d_{\max}})^2 \xi(l)^2 \zeta \,.
\end{aligned} \tag{31}$$

Proof. First we note that by the condition on ζ we have that the restriction to each neighborhood $\mathcal{N}(l)_i$ of $2\hat{V} - i\Omega$ is strictly positive and, as such, all the local inverses exist.

More specifically, by Lemma 6.4 applied to the matrices $M = (2V - i\Omega)^{-1}$, $\hat{N} = 2\hat{V} - i\Omega$, the neighborhood $\mathcal{N}(l)$ of Definition 6.5 and E as defined in Lemma 6.1, we have that

$$\begin{aligned}
&\|(2V - i\Omega)^{-1} - \mathrm{LI}_{\mathcal{N}(l)}(2\hat{V} - i\Omega)\|_\infty \\
&\quad \leq 2 \max_{\delta \in \{0,1\}} \xi(l) \|B_{i,\delta} - \widetilde{B}_{i,\delta}\|_\infty \|M^{-1}\|_\infty \|M\|_\infty + (2\xi(l)+1)\|E\|_\infty + 8\|M\|_\infty^2 \xi(l)^2 \zeta
\end{aligned}$$

as long as $\zeta \leq \frac{1}{4\xi(l)} \|M\|_\infty^{-1}$ and $M - E = \mathrm{LOC}_{\mathcal{N}(l)}(M - E)$. The first condition is satisfied by Equation (61) in Lemma 9.1, which states that $\|M\|_\infty^{-1} \geq \frac{2e^{-2d_{\max}}}{\|S\|_\infty^2 (1-e^{-2d_{\max}})}$. The second condition follows from the series approximation of Lemma 6.1, since

$$M - E = (2V - i\Omega)^{-1} - E = \frac{1}{2} \sum_{n=1}^{l} \frac{(2Hi\Omega)^n}{n!} (i\Omega) \,,$$

so that, for any two vertices i, j of the graph G, $(M - E)_{2i-\delta, 2j-\delta'}$ is non-zero only if j is at distance at most l from i. Using once again Equation (60) together with (61) of Lemma 9.1, as well as by noticing that, for any $i \in [m]$ and $\delta \in \{0,1\}$, $B_{i,\delta} - \widetilde{B}_{i,\delta}$ is a sub-row of E so that $\|B_{i,\delta} - \widetilde{B}_{i,\delta}\|_\infty \leq \|E\|_\infty$, we find the following bound:

$$\begin{aligned}
\|M - \mathrm{LI}_{\mathcal{N}(l)}(\hat{N})\|_\infty &\leq 2\xi(l)\|E\|_\infty \|S\|_\infty^4 \frac{(1 - e^{-2d_{\max}})e^{2d_{\max}}}{1 - e^{-2d_{\min}}} + (2\xi(l)+1)\|E\|_\infty \\
&\quad + 2\|S\|_\infty^4 e^{4d_{\max}} (1 - e^{-2d_{\max}})^2 \xi(l)^2 \zeta \,.
\end{aligned}$$

Finally, the result follows after using the bound $\|E\|_\infty \leq \frac{\|S\|_\infty^2}{2} \left(\frac{(2d_{\max})^{l+1} e^{2d_{\max}}}{(l+1)!} \right)$ derived in Lemma 6.1. □

We now need the following lemma.

Lemma 6.6. *Let $C_1, C_2, \epsilon > 0$ and let $W(x)$ be the principal branch of the Lambert W function defined through $e^{W(x)} W(x) = x$ for $x > 0$. If we take $l = \lfloor (C_2 e) \exp\left(W\left(\frac{\log \frac{C_1}{\epsilon}}{C_2 e} \right) \right) \rfloor$, then $C_1 \frac{C_2{}^{l+1}}{(l+1)!} \leq \epsilon$ and $\lim_{\epsilon \to 0} \Delta^l \epsilon^\gamma = 0$ for every $\gamma > 0$.*

Proof. We have

$$C_1 \frac{(C_2)^{l+1}}{(l+1)!} \leq C_1 \left(\frac{C_2 e}{l+1} \right)^{l+1},$$

and taking logarithms, we have

$$\begin{aligned}
\log \frac{C_1 \left(\frac{C_2 e}{l+1}\right)^{l+1}}{\epsilon} &= -(l+1) \log \left(\frac{l+1}{C_2 e} \right) + \log \frac{C_1}{\epsilon} \\
&\leq -(C_2 e) e^{W\left(\frac{\log \frac{C_1}{\epsilon}}{C_2 e}\right)} W \left(\frac{\log \frac{C_1}{\epsilon}}{C_2 e} \right) + \log \frac{C_1}{\epsilon} \\
&= -C_2 e \left(\frac{\log \frac{C_1}{\epsilon}}{C_2 e} \right) + \log \frac{C_1}{\epsilon} = 0,
\end{aligned} \tag{32}$$

so that $C_1 \left(\frac{C_2 e}{l+1}\right)^{l+1} \leq \epsilon$. An upper bound on the Lambert W function is $W(x) \leq \log \left(\frac{2x+1}{1+\log(x+1)} \right)$, see [38]. So, with our choice, $l \leq \frac{2 \log \frac{C_1}{\epsilon} + C_2 e}{1+\log\left(\frac{\log \frac{C_1}{\epsilon}}{C_2 e}+1\right)}$, and $\lim_{\epsilon \to 0} \Delta^l \epsilon^\gamma = 0$ for every $\gamma > 0$.

□

The following remark is the basis for our sample complexity analysis.

Remark 6.4. *In the worst case, $\xi(l) \leq \min\{m, \Delta^l\}$, where $\Delta - 1$ is the degree of the graph. Hence, the bound found in Lemma 6.5 simplifies to*

$$\|(2V - i\Omega)^{-1} - \mathrm{LI}_{\mathcal{N}(l)}(2\hat{V} - i\Omega)\|_\infty \quad \leq C(S, d_{\min}, d_{\max}) \frac{(2\Delta d_{\max})^{l+1}}{(l+1)!} + C'(S, d_{\min}, d_{\max})\, \Delta^{2l} \zeta\,,$$

with $C(S, d_{\min}, d_{\max}) := 4\|S\|_\infty^6 e^{4d_{\max}} \frac{1-e^{-2d_{\max}}}{1-e^{-2d_{\min}}}$ and $C'(S, d_{\min}, d_{\max}) := 2\|S\|_\infty^4 e^{4d_{\max}} (1 - e^{-2d_{\max}})^2$. Let us also denote $C''(\Delta, d_{\max}) = 2\Delta d_{\max} e$. Therefore, by choosing

$$l = \lfloor C'' \exp \left(W \left(\frac{\log \frac{C}{\epsilon'}}{C''} \right) \right) \rfloor, \quad \zeta = \min \left\{ \frac{\epsilon'}{C' \Delta^{2l}}, \frac{e^{-2d_{\max}}}{2\Delta^l \|S\|_\infty^2 (1 - e^{-2d_{\max}})} \right\},$$

we obtain, via Lemma 6.6

$$\|(2V - i\Omega)^{-1} - \mathrm{LI}_{\mathcal{N}(l)}(2\hat{V} - i\Omega)\|_\infty \leq 2\epsilon'\,.$$

Using Equation (23) as well as the perturbation bound in Lemma 5.2, and denoting by $\hat{H}$ the matrix associated to $\hat{V}$ through the following integral representation:

$$\hat{H} = \mathrm{LI}_{\mathcal{N}}(2\hat{V} - i\Omega) i\Omega \int_0^\infty \frac{I}{I + \frac{t}{t+1} 2\, \mathrm{LI}_{\mathcal{N}}(2\hat{V} - i\Omega) i\Omega} \frac{dt}{(t+1)^2} i\Omega\,, \tag{33}$$

we get

$$\|H - \hat{H}\|_\infty \leq e^{2d_{\max}} \|S\|_\infty^2 2\epsilon' \left(1 + \frac{e^{2d_{\max}} \|S\|_\infty^2 (1 - e^{-2d_{\max}}) + 4\epsilon'}{e^{-2d_{\max}} \|S\|_\infty^{-2} - 4\epsilon'}\right) ,$$

provided $4\epsilon' < e^{-2d_{\max}} \|S\|_\infty^{-2}$. *Choosing* $\epsilon' = \epsilon \frac{e^{-2d_{\max}} \|S\|_\infty^{-2}}{8\left(1+e^{4d_{\max}} \|S\|_\infty^4 (1-e^{-2d_{\max}})\right)} < \frac{e^{-2d_{\max}} \|S\|_\infty^{-2}}{4}$ *we have*

$$\left(1 + \frac{e^{2d_{\max}} \|S\|_\infty^2 (1 - e^{-2d_{\max}}) + 4\epsilon'}{e^{-2d_{\max}} \|S\|_\infty^{-2} - 4\epsilon'}\right) \leq 2 \left(1 + e^{4d_{\max}} \|S\|_\infty^4 (1 - e^{-2d_{\max}})\right) ,$$

and thus

$$\|H - \hat{H}\|_\infty \leq \epsilon \,.$$

7 Supplementary note 7: Gaussian Hamiltonian learning

The Hamiltonian learning problem consists on learning an estimate of the Hamiltonian of a Gaussian state from copies of it. More formally:

Definition 7.1 (Problem 2: Gaussian Hamiltonian learning)**.** *Let* $H \in M_{2m}^{>0}(\mathbb{R})$ *be a symmetric, positive definite matrix (later referred to as Gaussian Hamiltonian) and* $t \in \mathbb{R}^{2m}$. *For any such data* (H, t), *one can construct a Gaussian state on* m *modes:*

$$\rho(H, t) = \frac{e^{-(R-t)^\intercal H (R-t)}}{\mathrm{tr}[e^{-(R-t)^\intercal H (R-t)}]} \,.$$

The Gaussian Hamiltonian learning problem consists in the following task: given a fixed precision $\epsilon > 0$, *provide estimates* $\hat{H}$ *of* H *and* $\hat{t}$ *of* t *such that* $\|\hat{H} - H\|_\infty \leq \epsilon$ *and* $\|\hat{t} - t\|_\infty \leq \epsilon$ *with probability at least* $1 - \delta$, *given* n *copies of* $\rho(H, t)$, *for any* (H, t) *in some specified subset* $\mathcal{S}$. *We denote by* $\mathcal{N}_{\mathrm{HL}}(\mathcal{S}, m, \epsilon, \delta)$ *the sample complexity for Gaussian Hamiltonian learning. This corresponds to the minimum number* n *of copies of the state* $\rho(H, t)$ *required for the task to succeed.*

We consider two regimes of interest. It follows from Equation (31) that the growth rate of $\xi(l)$, i.e. how many vertices are at a distance at most l, governs how good the approximation of the local inverse is. Thus, we will first consider the physically relevant regime in which $\xi(l)$ is polynomial in l, as it does for lattices, and then handle the general case where we just assume a bound on the maximal degree of the graph.

Theorem 7.1 (Hamiltonian learning, polynomially growing neighborhoods)**.** *Let* H *be a Gaussian Hamiltonian with known interaction graph* G *such that* $\xi(l) \leq g l^r$ *for some* $g, r \geq 1$, *and such that* $H = S^{-\intercal} D S^{-1}$, *with* S *symplectic,* D *positive definite, diagonal with entries* $(d_1, d_1, ..., d_m, d_m)$, *and* $d_{\max} = \max_{i \in [m]} d_i$, $d_{\min} = \min_{i \in [m]} d_i$. *Furthermore, to simplify the presentation, we let:*

$$C_1 := \frac{e^{4d_{\max}} \|S\|_\infty^6}{2} \frac{1 - e^{-2d_{\max}}}{1 - e^{-2d_{\min}}}, \quad C_2 := \frac{e^{2d_{\max}} \|S\|_\infty^2}{2}, \quad C_3 := 2\|S\|_\infty^4 e^{4d_{\max}}, \tag{34}$$

$$C_4 := 4C_1 g (2d_{\max} e)^r \qquad C_5 = \frac{e^{-2d_{\max}} \|S\|_\infty^{-2}}{8\left(1 + e^{4d_{\max}} \|S\|_\infty^4 (1 - e^{-2d_{\max}})\right)}.$$

Given $1 > \epsilon > 0$ and $\delta \in (0,1)$ and for any $t \in \mathbb{R}^{2m}$,

$$N = \frac{C_3^2}{C_5^2}\left(g^2\left[r + (2d_{\max}e)e^{W(\frac{1}{2d_{\max}e}\log\frac{C_4}{C_5\epsilon})}\right]^{2r}\right)^2 \tag{35}$$

$$\times \frac{2^{16}(\|S\|_\infty^2\frac{1}{1-e^{-2d_{\min}}} + \max_i|t_i|^2)^2(1+\max_i|t_i|)^2}{\epsilon^2}\log\left(\frac{4m+1}{\delta}\right) \tag{36}$$

copies of the Gaussian state with parameters (H,t) suffice to derive estimates $\hat{H}$ and $\hat{t}$ such that $\|\hat{H} - H\|_\infty$, $\|\hat{t} - t\|_\infty \leq \epsilon < 1$, with probability larger than $1-\delta$. In particular, for $r = \mathcal{O}(1)$, focusing on the scaling in terms of ϵ and m we get:

$$N = \mathcal{O}(\epsilon^{-2}\log^{4r}(\epsilon^{-1})\log(m)). \tag{37}$$

Proof. Let us start by controlling how to pick l to ensure that the r.h.s. of Equation (31) is at most ϵ' for some $\epsilon_1 > 0$ to be fixed later. Existence of local inverses requires $\zeta < \frac{1}{2(\xi(l))}\frac{e^{-2d_{\max}}}{\|S\|_\infty^2(1-e^{-2d_{\max}})}$, which will be automatically ensured by the later choice of ζ.

The bound then becomes:

$$\begin{aligned}\|(2V - i\Omega)^{-1} - \mathrm{LI}_{\mathcal{N}(l)}(2\hat{V} - i\Omega)\|_\infty &\leq C_1\frac{\xi(l)(2d_{\max})^{l+1}}{(l+1)!} \\ &\quad + C_2\frac{(2\xi(l)+1)(2d_{\max})^{l+1}}{(l+1)!} + (1-e^{-2d_{\max}})^2C_3\xi(l)^2\zeta \\ &\leq 4C_1g(l+1)^r\frac{(2d_{\max})^{l+1}}{(l+1)!} + C_3\xi(l)^2\zeta,\end{aligned} \tag{38, 39}$$

where we used $g > 1$ and $C_1 \geq C_2$. Taking $l = \lfloor r + (2d_{\max}e)e^{W(\frac{1}{2d_{\max}e}\log\frac{C_4}{\epsilon_1})}\rfloor$ and assuming $\epsilon_1 < C_4$, we have that:

$$\begin{aligned}4C_1g(l+1)^r\frac{(2d_{\max})^{l+1}}{(l+1)!} &\leq 4C_1g(l+1)^r\frac{(2d_{\max}e)^{l+1}}{(l+1)^{l+1}} \\ &\leq 4C_1g(2d_{\max}e)^r\frac{(2d_{\max}e)^{l+1-r}}{(l+1-r)^{l+1-r}} \leq \epsilon_1,\end{aligned} \tag{40}$$

similarly to Eq. (32). Thus, we see that if we pick

$$\zeta = \epsilon_1C_3^{-1}\left(g^2\left[r + (2d_{\max}e)e^{W(\frac{1}{2d_{\max}e}\log\frac{C_4}{\epsilon_1})}\right]^{2r}\right)^{-1}, \tag{41}$$

then we have that:

$$\|(2V - i\Omega)^{-1} - \mathrm{LI}_{\mathcal{N}(l)}(2\hat{V} - i\Omega)\|_\infty \leq 2\epsilon_1. \tag{42}$$

Following the same analysis as in Remark 6.4, we get that setting ϵ_1 as

$$\epsilon_1 = \min\{\epsilon\frac{e^{-2d_{\max}}\|S\|_\infty^{-2}}{8\,(1 + e^{4d_{\max}}\|S\|_\infty^4(1-e^{-2d_{\max}}))}, C_4\} \tag{43}$$

is sufficient to ensure that $\|H' - H\|_\infty \leq \epsilon$. Putting all the elements together, we see that we need to estimate the entries of $2V$ to precision

$$\zeta = \left(g^2 \left[r + (2d_{\max}e)e^{W(\frac{1}{2d_{\max}e}\log\frac{C_4}{C_5\epsilon})} \right]^{2r} \right)^{-1} \times \epsilon \frac{e^{-2d_{\max}}\|S\|_\infty^{-2}}{8C_3\left(1+e^{4d_{\max}}\|S\|_\infty^4(1-e^{-2d_{\max}})\right)}, \tag{44}$$

which yields the advertised bound. □

Remark 7.1. *For Hamiltonians associated to graphs with polynomial neighborhood growth, one can expect exponential decay of correlations if* $d_{\min}$ *is uniformly bounded. For example Refs. [39, 40] consider some subclasses of such Hamiltonians and show that the correlation length, in our notation, is roughly* $\xi \sim \frac{\Delta}{\left(\frac{d_{\min}}{d_{\max}}\right)^\alpha}$ *for some* $\alpha > 0$ *(if the inverse temperature is set to* $d_{\max}$*,* $(\frac{d_{\min}}{d_{\max}})$ *can also be interpreted as the energy gap of the Hamiltonian). If* ξ *does not scale with* m*, the correlation decay is strong enough so that one could estimate only a number of entries of* V *that does not scale with* m *to error* ϵ*, set the entries corresponding to far vertices to zero, and still obtain an estimate of* V *which is of error* $\mathcal{O}(\epsilon)$*. This would allow us to use the continuity bound of Lemma 5.1 to obtain an* $\mathcal{O}(\epsilon)$ *error on the estimate of* H*. However,* ξ *is* $\mathcal{O}(m^\alpha)$ *if the energy gap scales with* $1/m$*, so that the above strategy would not be enough to guarantee* $\mathcal{O}(\mathrm{poly}\log(m))$ *sample complexity. Instead, the estimation algorithm using local inversions has a sample complexity upper bounded by a polynomial function of* $\max_i |V_{ii}|$ *and* $\log\frac{1-e^{-2d_{\max}}}{1-e^{-2d_{\min}}} \leq 2d_{\min} + \log\frac{d_{\max}}{d_{\min}} \leq 2d_{\max} + \log\frac{d_{\max}}{d_{\min}}$*, making the sample complexity still* $\mathcal{O}(\mathrm{poly}\log(m))$ *if* $\max_i |V_{ii}|$ *does not scale with* m *and* $d_{\max}$ *is uniformly bounded.*

Thus, we see that assuming polynomially growing neighborhoods, it is possible to obtain a sample complexity that scales like $\epsilon^{-2}\log(m)$, up to polylogarithmic factors and, of course, neglecting the dependence on the various other parameters of the problem. In the setting where we just assume a bound on the maximal degree of the interaction graph we obtain a higher sample complexity:

Theorem 7.2 (Hamiltonian learning, bounded degree)**.** *Let* H *be a Gaussian Hamiltonian with known interaction graph* G*, with degree* $\Delta - 1$*, such that* $H = S^{-\intercal}DS^{-1}$*, with* S *symplectic,* D *positive definite, diagonal with entries* $(d_1, d_1, ..., d_m, d_m)$*, and* $d_{\max} = \max_{i\in[m]} d_i$*,* $d_{\min} = \min_{i\in[m]} d_i$*. Given* $1 > \epsilon > 0$ *and* $\delta \in (0,1)$*, choose*

$$\begin{aligned}
\epsilon' &\leq \frac{\epsilon e^{-2d_{\max}}\|S\|_\infty^{-2}}{8}\frac{1}{1+e^{4d_{\max}}\|S\|_\infty^4(1-e^{-2d_{\max}})} \\
l &= \lfloor (2\Delta d_{\max}e)e^{\left(W\left(\frac{\log\frac{C}{\epsilon'}}{2\Delta d_{\max}e}\right)\right)} \rfloor \\
\zeta &\leq \min\left\{ \frac{\epsilon'}{C'\Delta^{2l}}, \frac{e^{-2d_{\max}}}{2\Delta^l\|S\|_\infty^2(1-e^{-2d_{\max}})} \right\},
\end{aligned}$$

where $C(S, d_{\min}, d_{\max}) := 4\|S\|_\infty^6 e^{4d_{\max}}\frac{1-e^{-2d_{\max}}}{1-e^{-2d_{\min}}}$ *and* $C'(S, d_{\min}, d_{\max}) := 2\|S\|_\infty^4 e^{4d_{\max}}(1-e^{-2d_{\max}})^2$*.*

Then, for any $t \in \mathbb{R}^{2m}$*,*

$$N = \frac{2^{16}(\|S\|_\infty^2\frac{1}{1-e^{-2d_{\min}}} + \max_i |t_i|^2)^2(1+\max_i |t_i|)^2}{\zeta^2}\log\left(\frac{4m+1}{\delta}\right) \tag{45}$$

copies of the Gaussian state with parameters (H, t) *suffice to derive estimates* $\hat{t}$ *and* $\hat{H}$ *(via Algorithm 1 with parameter* l*) such that* $\|\hat{H} - H\|_\infty$*,* $\|\hat{t} - t\|_\infty \leq \epsilon$*, with probability larger than* $1 - \delta$*.*

Algorithm 1 LearnHamiltonianGaussian

1: Input: estimate $\hat{V}$ of the covariance matrix V of m-mode Gaussian state ρ, graph G, distance parameter l

2: Set $\mathcal{N} = \{\mathcal{N}_i(l)\}_{i\in[m]}$, where $\mathcal{N}_i(l)$ is the set of nodes at distance at most l from i in the graph G.

3: Compute the estimator $\mathrm{LI}_{\mathcal{N}}(2\hat{V} - i\Omega)$ of $(2V - i\Omega)^{-1}$ as in Definition 6.3

4: Estimate H as:

$$\hat{H} = \mathrm{LI}_{\mathcal{N}}(2\hat{V} - i\Omega)i\Omega \int_0^\infty \frac{I}{I + \frac{t}{t+1}2\,\mathrm{LI}_{\mathcal{N}}(2\hat{V} - i\Omega)i\Omega} \frac{dt}{(t+1)^2} i\Omega\,,$$

5: Output $\hat{H}$.

Box 1: Algorithm for Hamiltonian learning for a known graph. Replication of Box 1 in the main text.

Proof. It follows from Remark 6.4 and Lemma 3.1. □

The following corollary is an immediate consequence:

Corollary 7.1 (Gaussian Hamiltonian learning, general graphs)**.** *Let $\mathcal{S}$ be the class of Hamiltonians such that $\|S\|_\infty \le s$, $d_{\max} \le \beta_{\max}$, $d_{\min} \ge \beta_{\min}$, $\max_i |t_i| \le t_{\max}$, and degree $\Delta - 1$. Then, with $C = \frac{s(1-e^{-2\beta_{\max}})}{1-e^{-2\beta_{\min}}} e^{2\beta_{\max}}$, for every $\gamma > 0$,*

$$\mathcal{N}(\mathcal{S}, m, \epsilon, \delta) = \log\left(\frac{4m+1}{\delta}\right) \mathcal{O}\left(\frac{\mathrm{poly}\{\Delta^{\Delta\beta_{\max}} C \left(\frac{C}{\epsilon}\right)^{\gamma/\Delta\beta_{\max}} (1 + t_{\max})\}}{\epsilon^2}\right). \tag{46}$$

Remark 7.2. *As already highlighted in Remark 4.1, bounding V_{ii} by $\|V\|_\infty$ and thus in terms of $d_{\min}$ may be overshooting in the regime of large m. Here, since a dependence on $d_{\min}$ appears anyway from the rest of the proof, we expressed everything in terms of $d_{\min}$. However, the only other dependence on $d_{\min}$ comes from bounding $\|D^{-1}C\|_\infty$ in Lemma 6.2. As noted in Remark 6.2, one could expect that bounding $\|D^{-1}C\|_\infty$ via the condition number of $(2V - i\Omega)^{-1}$ is also a loose bound, and the proof can instead be expressed in terms of a uniform bound on all the instances of $\|D^{-1}C\|_\infty$ from each call to Remark 6.2. Putting together these observations suggests that in practice the number of copies needed could be much smaller than what the corollary above guarantees. The same observation applies to our later Theorem 8.1.*

8 Supplementary note 8: Learning the graph

We now consider the problem of learning the interaction graph, defined as follows.

Definition 8.1 (Problem 3: Gaussian graph learning)**.** *Let $H \in M_{2m}^{>0}(\mathbb{R})$ be a symmetric, positive definite matrix (later referred to as Gaussian Hamiltonian) and $t \in \mathbb{R}^{2m}$. For any such data (H, t),*

one can construct a Gaussian state on m modes:

$$\rho(H,t)=\frac{e^{-(R-t)^\intercal H(R-t)}}{\mathrm{tr}[e^{-(R-t)^\intercal H(R-t)}]}\,.$$

The Gaussian graph learning problem consists in the following task: provide an estimate $\hat{\mathsf{E}}$ of the edge set E of the graph of H, such that $\hat{\mathsf{E}}=\mathsf{E}$ with probability larger than $1-\delta$ given n copies of $\rho(H,t)$, for any (H,t) in some specified subset $\mathcal{S}$ which satisfies a lower bound κ on the maximum absolute value of the entries of non-zero sub-matrices of the Hamiltonian:

$$\kappa\le\min_{(i,j)\in\mathsf{E}}\max_{\delta_1,\delta_2\in\{0,1\}}|H_{2i-\delta_1,2j-\delta_2}|\,.$$

We denote by $\mathcal{N}_{\mathrm{GL}}(\mathcal{S},\kappa,m,\delta)$ the sample complexity for Gaussian graph learning. This corresponds to the minimum number n of copies of the state $\rho(H,t)$ required for the task to succeed.

Again, this is the natural quantum variation of learning the structure of a Gaussian graphical model [32]. We will need the following variant of Lemma 6.3 where the neighborhood structure is also being approximated.

Lemma 8.1. *Let M be a positive definite matrix and let $\mathcal{N}=\{\mathcal{N}_i\}_{i\in[m]}$, $\hat{\mathcal{N}}=\{\hat{\mathcal{N}}_i\}_{i\in[m]}$ be two neighborhood structures of $[m]$. For each $i\in[m]$ and $\delta\in\{0,1\}$, let $\left(\begin{smallmatrix}A_{i,\delta}&B_{i,\delta}\\C_{i,\delta}&D_{i,\delta}\end{smallmatrix}\right)\in\mathbb{C}^{2m\times 2m}$, with blocks $A_{i,\delta}\in\mathbb{C}^{2|\hat{\mathcal{N}}_i|\times 2|\hat{\mathcal{N}}_i|}$, $B_{i,\delta}\in\mathbb{C}^{2|\hat{\mathcal{N}}_i|\times(2m-2|\hat{\mathcal{N}}_i|)}$, $C_{i,\delta}\in\mathbb{C}^{(2m-2|\hat{\mathcal{N}}_i|)\times 2|\hat{\mathcal{N}}_i|}$, $D_{i,\delta}\in\mathbb{C}^{(2m-2|\hat{\mathcal{N}}_i|)\times(2m-2|\hat{\mathcal{N}}_i|)}$ be the matrix $\pi_{i,\delta}M\pi_{i,\delta}^{-1}$ where $\pi_{i,\delta}$ is a permutation that brings the coefficients $2j-\delta'$ with $\delta'\in\{0,1\}$ and $j\in\hat{\mathcal{N}}_i$ to the upper-left block $A_{i,\delta}$, in such a way that index $2i-\delta$ is mapped to 1 for $\delta=0,1$. Let $\widetilde{B}_{i,\delta}$ be the matrix constructed from $B_{i,\delta}$ by putting the first row to zero. Let E be a self-adjoint matrix, such that $\mathrm{LOC}_{\mathcal{N}}(M-E)=M-E$. Then,*

$$\begin{aligned}\|M-\mathrm{LI}_{\hat{\mathcal{N}}}(M^{-1})\|_\infty\le\;&2\max_{i\in[m]}\max_{\delta\in\{0,1\}}|\hat{\mathcal{N}}_i|\|B_{i,\delta}-\widetilde{B}_{i,\delta}\|_\infty\|M^{-1}\|_\infty\|M\|_\infty+(2\max_{i\in[m]}|\hat{\mathcal{N}}_i|+1)\|E\|_\infty\\&+\max_{i\in[m]}\max_{\delta_1\in\{0,1\}}\sum_{j\in\mathcal{N}_i,j\notin\hat{\mathcal{N}}_i,\delta_2\in\{0,1\}}|(M-E)_{2i-\delta_1,2j-\delta_2}|.\end{aligned}\tag{47}$$

Proof. The proof is essentially the same as that of Lemma 6.3, except that in the final application of Gershgorin's theorem the matrix $M-E-\mathrm{LI}_{\hat{\mathcal{N}}}(M^{-1})$ can have non-zero elements for entries corresponding to the neighborhood structure $\mathcal{N}$ but not $\hat{\mathcal{N}}$. We reproduce the aforementioned proof with the necessary changes. By the triangle inequality, we have

$$\|M-\mathrm{LI}_{\hat{\mathcal{N}}}(M^{-1})\|_\infty\le\|E\|_\infty+\|M-E-\mathrm{LI}_{\hat{\mathcal{N}}}(M^{-1})\|_\infty\,.$$

It remains to bound the last operator norm above. Since $\mathrm{LOC}_{\mathcal{N}}(M-E)-\mathrm{LI}_{\hat{\mathcal{N}}}(M^{-1})=M-E-\mathrm{LI}_{\hat{\mathcal{N}}}(M^{-1})$ is self-adjoint, it suffices to find a uniform bound on its eigenvalues. By Gershgorin's circle theorem, denoting the matrix $L:=\mathrm{LOC}_{\mathcal{N}}(M-E)-\mathrm{LI}_{\hat{\mathcal{N}}}(M^{-1})$, these eigenvalues are situated in the $2m$ intervals

$$\left[L_{k,k}-\sum_{k'\neq k}|L_{k,k'}|,\,L_{k,k}+\sum_{k'\neq k}|L_{k,k'}|\right],\;k\in[2m]\,.$$

Now for any $i \in [m]$, $j \in \hat{\mathcal{N}}_i$ and $\delta' \in \{0,1\}$, we have by the construction of the local inverse matrix, denoting $N = M^{-1}$, and N_i the block matrices defined similarly to Definition 6.3 but corresponding to the neighborhood structure $\hat{\mathcal{N}}$:

$$\left|M_{2i-\delta,2j-\delta'} - \left(\mathrm{LI}_{\hat{\mathcal{N}}}(M^{-1})\right)_{2i-\delta,2j-\delta'}\right| \leq \frac{1}{2}\left|M_{2i-\delta,2j-\delta'} - \left(N_i^{-1}\right)_{2i-\delta,2j-\delta'}\right| + \frac{1}{2}\left|M_{2i-\delta,2j-\delta'} - \left(N_j^{-1}\right)^*_{2j-\delta',2i-\delta}\right|.$$

Via Lemma 6.2 applied to the matrices $\pi_{i,\delta} M \pi_{i,\delta}^{-1}$ and $\pi_{j,\delta'} M \pi_{j,\delta'}^{-1}$,

$$\left|M_{2i-\delta,2j-\delta'} - \left(N_i^{-1}\right)_{2i-\delta,2j-\delta'}\right| \leq \|B_{i,\delta} - \widetilde{B}_{i,\delta}\|_\infty \|M^{-1}\|_\infty \|M\|_\infty\,,$$

$$\left|M_{2i-\delta,2j-\delta'} - \left(N_j^{-1}\right)^*_{2j-\delta',2i-\delta}\right| = \left|M^*_{2j-\delta',2i-\delta} - \left(N_j^{-1}\right)^*_{2j-\delta',2i-\delta}\right| \leq \|B_{j,\delta'} - \widetilde{B}_{j,\delta'}\|_\infty \|M^{-1}\|_\infty \|M\|_\infty$$

and hence $|L_{2i-\delta,2j-\delta'}| \leq |(M - \left(\mathrm{LI}_{\hat{\mathcal{N}}}(M^{-1})\right)_{2i-\delta,2j-\delta'}| + |E_{2i-\delta,2j-\delta'}| \leq \max_{i\in[m]} \max_{\delta\in\{0,1\}} \|B_{i,\delta} - \widetilde{B}_{i,\delta}\|_\infty \|M^{-1}\|_\infty \|M\|_\infty + \|E\|_\infty$. The other non-zero entries of L are $L_{2i-\delta_1,2j-\delta_2}$ such that $j \in \mathcal{N}_i$ but $j \notin \hat{\mathcal{N}}_i$, and $L_{2i-\delta_1,2j-\delta_2} = (M-E)_{2i-\delta_1,2j-\delta_2}$. Using Gershgorin's theorem together with the fact that the matrix L has at most $2\max_i |\hat{\mathcal{N}}_i|$ non-zero coefficients per column that corresponds to $j \in \hat{\mathcal{N}}_i$, we get

$$\|L\|_\infty \leq 2 \max_{i\in[m]} \max_{\delta\in\{0,1\}} |\hat{\mathcal{N}}_i| \left(\|B_{i,\delta} - \widetilde{B}_{i,\delta}\|_\infty \|M^{-1}\|_\infty \|M\|_\infty + \|E\|_\infty\right) + \max_{i\in[m]} \max_{\delta_1\in\{0,1\}} \sum_{j\in\mathcal{N}_i, j\notin\hat{\mathcal{N}}_i, \delta_2\in\{0,1\}} |(M-E)_{2i-\delta_1,2j-\delta_2}|\,.$$

The result follows. □

The error made during the local inversion can be bounded as in Lemma 6.4:

Lemma 8.2. *In the notations of Lemma 8.1, given a $2m\times 2m$ positive definite matrix M, $N = M^{-1}$ and $\hat{N}$ a componentwise approximation of N, where $|\hat{N}_{k,k'} - N_{k,k'}| \leq \zeta$ for all $k,k' \in [2m]$ with $\zeta \leq \frac{1}{4\max_{i\in[m]} |\hat{\mathcal{N}}_i|}\|M\|_\infty^{-1}$,*

$$\|M - \mathrm{LI}_{\hat{\mathcal{N}}}(\hat{N})\|_\infty \leq 2 \max_{i\in[m]} \max_{\delta\in\{0,1\}} |\hat{\mathcal{N}}_i| \|B_{i,\delta} - \widetilde{B}_{i,\delta}\|_\infty \|M^{-1}\|_\infty \|M\|_\infty + (2\max_{i\in[m]} |\hat{\mathcal{N}}_i| + 1)\|E\|_\infty + \max_{i\in[m]} \max_{\delta_1\in\{0,1\}} \sum_{j\in\mathcal{N}_i, j\notin\hat{\mathcal{N}}_i, \delta_2\in\{0,1\}} |(M-E)_{2i-\delta_1,2j-\delta_2}| + 8\|M\|_\infty^2 \max_i |\hat{\mathcal{N}}_i|^2 \zeta\,. \quad (48)$$

Proof. By the triangle inequality,

$$\|M - \mathrm{LI}_{\hat{\mathcal{N}}}(\hat{N})\|_\infty \leq \|M - \mathrm{LI}_{\hat{\mathcal{N}}}(N)\|_\infty + \|\mathrm{LI}_{\hat{\mathcal{N}}}(N) - \mathrm{LI}_{\hat{\mathcal{N}}}(\hat{N})\|_\infty.$$

The first term is bounded as in Lemma 8.1, the second as in Lemma 6.4.

□

With these estimates at hand we can show that Algorithm 2 stated in pseudocode below outputs a correct estimate of the graph with high probability. Before stating the theorem, let us give an overview of the algorithm. The algorithm takes as input an estimate of the covariance matrix $\hat{V}$ and a neighborhood-size bound $\xi(l) \leq \Delta^l$, plus thresholding parameters η, κ. It then iterates over all vertices and all neighborhoods of size $\xi(l)$, denoted by $\mathcal{N}_i$. For each of the $\mathcal{N}_i$, we then consider all possible neighborhoods enlarged by $\xi(l)$ additional vertices, defining a set $\overline{\mathcal{N}}_i$, and perform our local inversion with the enlarged neighborhood. If we see that in the resulting matrix there are no large entries (larger than 3η) on row i and vertices $j \in \overline{\mathcal{N}}_i$, we then set our neighborhood as $\mathcal{N}_i$. The intuition behind the procedure is as follows: recall that we assume that we have a lower bound on the size of all interactions and that matrix elements of $(2V - i\Omega)^{-1}$ corresponding to vertices that are far apart decay rapidly. If we have the right l-neighborhood already for some vertex i, then all the estimates with the enlarged neighborhood will be approximately correct and all the entries added will be smaller than 3η because of the decay. Thus, the algorithm will correctly accept in this case. On the other hand, if we have the incorrect or incomplete neighborhood, then there must be $\xi(l)$ vertices we can add that will make it complete. For that joint neighborhood, we see two possibilities: either this will "turn on" a large entry, which will show us that we were not operating with the right neighborhood. In contrast, if we see that for all possibilities we add this never occurs, then we can also conclude that the vertices we are missing will only add a small error and the missing edges can be neglected. At the end, we end up with an estimate of the Hamiltonian matrix which has error at most $\kappa/3$ in operator norm, so that we can identify non-edges thanks to the promise on the strength of the interactions.

More formally, we have:

Theorem 8.1. *Let H be a Hamiltonian with graph G of degree $\Delta - 1$ and edge set E satisfying the condition $0 < \kappa \leq \min_{(i,j)\in\mathsf{E}} \max_{\delta_1,\delta_2\in\{0,1\}} |H_{2i-\delta_1,2j-\delta_2}|$. Suppose $H = S^{-\intercal} D S^{-1}$, with S symplectic, D diagonal with entries $(d_1, d_1, ..., d_m, d_m)$, and $d_{\max} = \max_{i\in[m]} d_i$, $d_{\min} = \min_{i\in[m]} d_i$. Let us assume that $\|S\|_\infty \leq s$, $d_{\max} \leq \beta_{\max}$, $d_{\min} \geq \beta_{\min}$. We also denote by $\xi(l)$ the maximum number of vertices at distance at most l from any given vertex. For l, ζ, ϵ', η given by*

$$
\begin{aligned}
l &= \left\lfloor (2\Delta^2\beta_{\max}e)\exp\left(W\left(\frac{\log\frac{C(s,\beta_{\min},\beta_{\max})}{\epsilon'}}{2\Delta^2\beta_{\max}e}\right)\right)\right\rfloor \\
\zeta &= \frac{\epsilon' e^{-6\beta_{\max}}}{200\,\Delta^{3l}s^8}\frac{1-e^{-2\beta_{\min}}}{1-e^{-2\beta_{\max}}} \\
\epsilon' &= \min\left(\kappa\frac{e^{-2\beta_{\max}}s^{-2}}{24\left(1+e^{4\beta_{\max}}s^4(1-e^{-2\beta_{\max}})\right)}, \frac{e^{-2\beta_{\max}}s^{-2}}{8}\right) \\
\eta &= \frac{\epsilon'}{e^{2\beta_{\max}}50\,\Delta^{2l}s^4}\frac{(1-e^{-2\beta_{\min}})}{(1-e^{-2\beta_{\max}})},
\end{aligned}
$$

where we denoted $C(s,\beta_{\min},\beta_{\max}) := 25s^{10}e^{6\beta_{\max}}\frac{(1-e^{-2\beta_{\max}})^2}{(1-e^{-2\beta_{\min}})^2}$ and $C'(s,\beta_{\max}) := 2s^4e^{4\beta_{\max}}$, given

$$
N = \mathcal{O}\left(\frac{(s^2\frac{1}{1-e^{-2\beta_{\min}}} + \max_i|t_i|^2)^2(1+\max_i|t_i|)^2}{\zeta^2}\log\left(\frac{4m+1}{\delta}\right)\right) \tag{49}
$$

copies of the Gaussian state with parameters (H,t), $\mathsf{LearnGraphGaussian}$ *(Algorithm 2) with parameters $\xi(l), \eta, \kappa$ outputs an estimate $\hat{\mathsf{E}}$ of the edge set E such that $\hat{\mathsf{E}} = \mathsf{E}$ with probability larger than*

$1-\delta$. *Furthermore, we can run Algorithm 2 in time*

$$\mathcal{O}\left(m\binom{m-\xi(l)}{\xi(l)}\binom{m}{\xi(l)}\xi(l)^3\right). \tag{50}$$

Algorithm 2 LearnGraphGaussian

```
Input: estimate V̂ of the covariance matrix V of ρ, distance parameter l, (bound on)
cardinality of neighborhood ξ(l), threshold parameters η, κ > 0.
for i ∈ [m] do
    for N_i candidate neighborhoods of i of cardinality ξ(l) do
        set N̂_i = N_i
        for N̄_i ∈ sets of vertices in [m]/N_i of cardinality ξ(l) do
            Compute {LI_{N_i ∪ N̄_i}(2V̂ − iΩ)_{2i−δ1,2j−δ2}, j ∈ N̄_i, δ1, δ2 ∈ {0,1}}
        end for
        if max_{N̄_i} max_{j∈N̄_i,δ1,δ2∈{0,1}} |LI_{N_i ∪ N̄_i}(2V̂ − iΩ)_{2i−δ1,2j−δ2}| ≤ 3η then
            break
        end if
    end for
end for
For each i ∈ [m], j ∈ N̂_i, eliminate element j from N̂_i if i ∉ N̂_j.
Compute estimate Ĥ of H from LI_{N̂}(2V̂ − iΩ) as in Equation 33.
Output the edges (i,j) such that max_{δ1∈{0,1},δ2∈{0,1}} |Ĥ_{2i−δ1,2j−δ2}| ≥ κ/2.
```

Box 2: Algorithm for learning the graph of a Gaussian state. Replication of Box 2 in the main text.

Proof. To learn the graph, we use Algorithm 2. The goal of the algorithm is to find a neighborhood $\hat{\mathcal{N}}_i$ of each $i\in[m]$ such that the local inversion gives accurate estimates of the matrix elements corresponding to edges including i. We use the notation of Lemma 8.1.

At each test step of Algorithm 2, local inversions with neighborhoods $\widetilde{\mathcal{N}_i}:=\mathcal{N}_i\cup\overline{\mathcal{N}}_i$ are performed, where $|\widetilde{\mathcal{N}_i}|\leq 2\xi(l)$ and $\eta>0$ is a fixed threshold parameter. Let us make the following assumptions:

- For any $j\in\widetilde{\mathcal{N}_i}$, $\delta_1,\delta_2\in\{0,1\}$, we have $|\,\mathrm{LI}_{\widetilde{\mathcal{N}}}(2V-i\Omega)_{2i-\delta_1,2j-\delta_2}-\mathrm{LI}_{\widetilde{\mathcal{N}}}(2\hat{V}-i\Omega)_{2i-\delta_1,2j-\delta_2}|\leq\eta$. We can ensure this by increasing the number of copies of $\rho(H,t)$ so that each entry of $\hat{V}$ is close to the corresponding entry of V with high probability. By the argument of (30), with $N=2V-i\Omega$, $\hat{N}=2\hat{V}-i\Omega$ and $M=(2V-i\Omega)^{-1}$, if $|2\hat{V}_{k,k'}-2V_{k,k'}|\leq\zeta$, with $\zeta\leq\frac{1}{8\xi(l)}\|(2V-i\Omega)^{-1}\|_\infty^{-1}$ we get $|\,\mathrm{LI}_{\widetilde{\mathcal{N}}}(2V-i\Omega)_{2i-\delta_1,2j-\delta_2}-\mathrm{LI}_{\widetilde{\mathcal{N}}}(2\hat{V}-i\Omega)_{2i-\delta_1,2j-\delta_2}|\leq 8\xi(l)\|(2V-i\Omega)^{-1}\|_\infty^2\zeta$. Thus, we need to ensure also

$$\zeta\leq\min\left(\frac{\|(2V-i\Omega)^{-1}\|_\infty^{-1}}{8\xi(l)},\frac{\|(2V-i\Omega)^{-1}\|_\infty^{-2}\eta}{8\xi(l)}\right). \tag{51}$$

- When $\mathcal{N}_i$ coincides with the l-neighborhood $\mathcal{N}(l)_i$ (see Definition 6.5), we assume that $\max_{\delta\in\{0,1\}} \|B_{i,\delta} - \widetilde{B}_{i,\delta}\|_\infty \|M^{-1}\|_\infty \|M\|_\infty \le \eta$, and at the same time $\|E\|_\infty \le \eta$ for the error E defined in Lemma 6.1, which we can always ensure by taking l large enough. In fact, it suffices to impose that

$$\|E\|_\infty \|M^{-1}\|_\infty \|M\|_\infty \le \|E\|_\infty \|S\|_\infty^4 \frac{(1-e^{-2d_{\max}})e^{2d_{\max}}}{1-e^{-2d_{\min}}} \le \eta\,, \tag{52}$$

since $B_{i,\delta} - \widetilde{B}_{i,\delta}$ is a sub-matrix of E and $\|S\|_\infty^4 e^{2d_{\max}} \frac{1-e^{-2d_{\max}}}{1-e^{-2d_{\min}}} \ge 1$.
For any $\mathcal{N}_i$ such that $\mathcal{N}_i(l) \subseteq \mathcal{N}_i$ the same relation will hold.

At each search step of the algorithm, there are two cases.

- $\mathcal{N}_i$ already contains $\mathcal{N}(l)_i$, the l-neighborhood of i. Then, all $\mathrm{LI}_{\mathcal{N}_i\cup\overline{\mathcal{N}}_i}(2\hat{V} - i\Omega)_{2i-\delta_1,2j-\delta_2}$ for $j \in \overline{\mathcal{N}}_i$, $\delta_1, \delta_2 \in \{0,1\}$, satisfy

$$\begin{aligned}
&|\,\mathrm{LI}_{\mathcal{N}_i\cup\overline{\mathcal{N}}_i}(2\hat{V} - i\Omega)_{2i-\delta_1,2j-\delta_2} - (2V - i\Omega)^{-1}_{2i-\delta_1,2j-\delta_2}| \\
&\qquad = |\,\mathrm{LI}_{\mathcal{N}_i\cup\overline{\mathcal{N}}_i}(2\hat{V} - i\Omega)_{2i-\delta_1,2j-\delta_2} - E_{2i-\delta_1,2j-\delta_2}| \\
&\qquad \le |\,\mathrm{LI}_{\mathcal{N}_i\cup\overline{\mathcal{N}}_i}(2\hat{V} - i\Omega)_{2i-\delta_1,2j-\delta_2} - \mathrm{LI}_{\mathcal{N}_i\cup\overline{\mathcal{N}}_i}(2V - i\Omega)_{2i-\delta_1,2j-\delta_2}| \\
&\qquad\quad + |\,\mathrm{LI}_{\mathcal{N}_i\cup\overline{\mathcal{N}}_i}(2V - i\Omega)_{2i-\delta_1,2j-\delta_2} - E_{2i-\delta_1,2j-\delta_2}|\,.
\end{aligned}$$

  The first term is bounded by η by assumption. The second term is bounded via Lemma 6.2 by $\|B_{i,\delta_1} - \widetilde{B}_{i,\delta_1}\|_\infty \|M^{-1}\|_\infty \|M\|_\infty$, since the off-diagonal blocks $B_{i,\delta}$ defined with respect to the neighborhood structure $\mathcal{N}$ include those defined for the set $\mathcal{N}_i \cup \overline{\mathcal{N}}_i$, implying

$$|\,\mathrm{LI}_{\mathcal{N}_i\cup\overline{\mathcal{N}}_i}(2\hat{V} - i\Omega)_{2i-\delta_1,2j-\delta_2}| \le \|B_{i,\delta_1} - \widetilde{B}_{i,\delta_1}\|_\infty \|M^{-1}\|_\infty \|M\|_\infty + \|E\|_\infty + \eta \le 3\eta\,.$$

- If $\mathcal{N}_i$ does not contain $\mathcal{N}(l)_i$, there is an adversarial neighborhood $\overline{\mathcal{N}}_i$ such that $\mathcal{N}_i \cup \overline{\mathcal{N}}_i$ contains $\mathcal{N}(l)_i$. Suppose there is at least one matrix element $|(2V - i\Omega)^{-1}_{2i-\delta_1,2j-\delta_2}| \ge 6\eta$ with $j \in \overline{\mathcal{N}}_i$,$\delta_1, \delta_2 \in \{0,1\}$ for some $\overline{\mathcal{N}}_i$ with the aforementioned property. Then, we have

$$\begin{aligned}
&|\,\mathrm{LI}_{\mathcal{N}_i\cup\overline{\mathcal{N}}_i}(2\hat{V} - i\Omega)_{2i-\delta_1,2j-\delta_2} - (2V - i\Omega)^{-1}_{2i-\delta_1,2j-\delta_2}| \\
&\qquad \le |\,\mathrm{LI}_{\mathcal{N}_i\cup\overline{\mathcal{N}}_i}(2\hat{V} - i\Omega)_{2i-\delta_1,2j-\delta_2} - \mathrm{LI}_{\mathcal{N}_i\cup\overline{\mathcal{N}}_i}(2V - i\Omega)_{2i-\delta_1,2j-\delta_2}| \\
&\qquad + |\,\mathrm{LI}_{\mathcal{N}_i\cup\overline{\mathcal{N}}_i}(2V - i\Omega)_{2i-\delta_1,2j-\delta_2} - (2V - i\Omega)^{-1}_{2i-\delta_1,2j-\delta_2}|,
\end{aligned}$$

  and by the same reason as before, we obtain

$$|\,\mathrm{LI}_{\mathcal{N}_i\cup\overline{\mathcal{N}}_i}(2\hat{V} - i\Omega)_{2i-\delta_1,2j-\delta_2}| \ge |(2V - i\Omega)^{-1}_{2i-\delta_1,2j-\delta_2}| - 2\eta \ge 4\eta\,. \tag{53}$$

This implies that at the end of the process we obtain a set of neighborhoods $\hat{\mathcal{N}} := \{\mathcal{N}_i\}_{i\in[m]}$ such that the matrix elements corresponding to edges which are in the true neighborhoods $\mathcal{N}_i(l)$ but not in $\hat{\mathcal{N}}_i$ are less than 6η in absolute value. The elimination steps ensure that $\{\hat{\mathcal{N}}_i\}$ form a neighborhood structure, and we omit only matrix elements smaller than 6η.

The error made when estimating $(2V - i\Omega)^{-1}$ by $\mathrm{LI}_{\hat{\mathcal{N}}}(2\hat{V} - i\Omega)$ is given by Lemma 8.2, as follows: with $M = (2V - i\Omega)^{-1}$ and E as in Equation 28, if $|2\hat{V}_{k,k'} - 2V_{k,k'}| \leq \zeta \leq 2\eta$ and $\zeta \leq \frac{1}{4\xi(l)}\|(2V - i\Omega)^{-1}\|_\infty^{-1}$, then,

$$\|M - \mathrm{LI}_{\hat{\mathcal{N}}}(2\hat{V} - i\Omega)\|_\infty \leq 2 \max_{i\in[m]} \max_{\delta\in\{0,1\}} |\hat{\mathcal{N}}_i| \|B_{i,\delta} - \widetilde{B}_{i,\delta}\|_\infty \|M^{-1}\|_\infty \|M\|_\infty + (2 \max_{i\in[m]} |\hat{\mathcal{N}}_i| + 1)\|E\|_\infty$$
$$+ \max_{i\in[m]} \max_{\delta_1\in\{0,1\}} \sum_{j\in\mathcal{N}(l)_i, j\notin\hat{\mathcal{N}}_i, \delta_2\in\{0,1\}} |(M - E)_{2i-\delta_1,2j-\delta_2}| + 8\|M\|_\infty^2 (\max_i |\hat{\mathcal{N}}_i|)^2 \zeta. \tag{54}$$

We can divide the complement of $\hat{\mathcal{N}}_i$ in two parts: a) the vertices that are in $\mathcal{N}(l)_i$ b) The vertices that are not in $\mathcal{N}(l)_i$. Accordingly, we can split $B_{i,\delta} - \widetilde{B}_{i,\delta}$ in two parts: the first part are the rows corresponding to a), the second the rows corresponding to b) and $B_{i,\delta} - \widetilde{B}_{i,\delta}$ can be bounded by the sum of the norms of the two sub-columns. Since the sub-column corresponding to b) is a submatrix of E, we can bound its norm by $\|E\|_\infty \leq \eta$. The sub-column corresponding to a) is instead made of at most $2\xi(l)$ entries which are bounded in absolute value by 6η, so its norm is bounded by $12\xi(l)\eta$. Thus, we have $\|B_{i,\delta} - \widetilde{B}_{i,\delta}\|_\infty \leq \|E\|_\infty + 12\xi(l)\eta \leq 13\xi(l)\eta$. Moreover, for $j \in \mathcal{N}(l)_i, j \notin \hat{\mathcal{N}}_i, \delta_1, \delta_2 \in \{0,1\}$, $|(M - E)_{2i-\delta_1,2j-\delta_2}| \leq |M_{2i-\delta_1,2j-\delta_2}| + \|E\|_\infty \leq 7\eta$, and $\max_{i\in[m]} \max_{\delta_1\in\{0,1\}} \sum_{j\in\mathcal{N}(l)_i, j\notin\hat{\mathcal{N}}_i, \delta_1,\delta_2\in\{0,1\}} |(M - E)_{2i-\delta_1,2j-\delta_2}| \leq 14\xi(l)\eta$.

Putting all these bounds together, we obtain after simplification

$$\begin{aligned}
&\|M - \mathrm{LI}_{\hat{\mathcal{N}}}(2\hat{V} - i\Omega)\|_\infty \\
&\quad\leq 26\xi(l)^2\eta\|S\|_\infty^4 \frac{1 - e^{-2d_{\max}}}{1 - e^{-2d_{\min}}} e^{2d_{\max}} + (16\xi(l) + 1)\eta + 2\xi(l)^2\zeta\|S\|_\infty^4 e^{4d_{\max}}(1 - e^{-2d_{\max}})^2 \\
&\quad\leq 50\,\xi(l)^2\eta\|S\|_\infty^4 \frac{1 - e^{-2d_{\max}}}{1 - e^{-2d_{\min}}} e^{2d_{\max}} + 2\xi(l)^2\zeta\|S\|_\infty^4 e^{4d_{\max}},
\end{aligned}$$

where in the last line we used that $\xi(l), \|S\|_\infty \geq 1$. Therefore, we can make the above term as small as we want if we can choose l as a function of η such that $\lim_{\eta\to 0} \xi(l(\eta))\eta = 0$, and all the above assumptions also hold. ζ is then computed as a function of $\xi(l(\eta))$. Say that we want to ensure $50\,\xi(l)^2\eta\|S\|_\infty^4 \frac{1-e^{-2d_{\max}}}{1-e^{-2d_{\min}}} e^{2d_{\max}} \leq \epsilon'$. Recall that from Equation (52) we also need to ensure $\|E\|_\infty\|S\|_\infty^4 \frac{1-e^{-2d_{\max}}}{1-e^{-2d_{\min}}} e^{2d_{\max}} \leq \eta$.

The former condition can be satisfied if we take

$$\eta = \frac{\epsilon'}{e^{2d_{\max}} 50\,\Delta^{2l}\|S\|_\infty^4} \frac{1 - e^{-2d_{\min}}}{1 - e^{-2d_{\max}}} \leq \frac{\epsilon'}{e^{2d_{\max}} 50\,\xi(l)^2\|S\|_\infty^4} \frac{1 - e^{-2d_{\min}}}{1 - e^{-2d_{\max}}},$$

since $\xi(l) \leq \Delta^l$, while the latter condition is implied by

$$\|E\|_\infty \leq \frac{\|S\|_\infty^2}{2} \left(\frac{(2d_{\max})^{l+1} e^{2d_{\max}}}{(l+1)!} \right) \overset{!}{\leq} \frac{\epsilon'}{e^{4d_{\max}} 50\,\Delta^{2l}\|S\|_\infty^8} \left(\frac{1 - e^{-2d_{\min}}}{1 - e^{-2d_{\max}}} \right)^2 \tag{55}$$

$$\leq \frac{\epsilon'}{e^{4d_{\max}} 50\,\xi(l)^2\|S\|_\infty^8} \left(\frac{1 - e^{-2d_{\min}}}{1 - e^{-2d_{\max}}} \right)^2, \tag{56}$$

where we further used the bound on $\|E\|_\infty$ derived in Lemma 6.1. This is ensured by taking (Lemma 6.6)

$$l = \left\lfloor (2\Delta^2 d_{\max} e) \exp\left(W\left(\frac{\log \frac{C(S,d_{\min},d_{\max})}{\epsilon'}}{2\Delta^2 d_{\max} e} \right)\right) \right\rfloor,$$

where we denoted $C(S, d_{\min}, d_{\max}) := 25\|S\|_\infty^{10} e^{6d_{\max}} \left(\frac{1-e^{-2d_{\max}}}{1-e^{-2d_{\min}}}\right)^2$. Then, $2\xi(l)^2\zeta\|S\|_\infty^4 e^{4d_{\max}} \leq \epsilon'$ implies that $\|M - \mathrm{LI}_{\hat{\mathcal{N}}}(2\hat{V} - i\Omega)\|_\infty \leq 2\epsilon'$. Moreover, assuming $\eta \leq 1$, we have that $\zeta \leq 2\eta$, $\zeta \leq \frac{1}{8\xi(l)}\|(2V - i\Omega)^{-1}\|_\infty^{-1} \leq \frac{1}{4\xi(l)}\|(2V - i\Omega)^{-1}\|_\infty^{-1}$, and $\zeta \leq \frac{\|(2V-i\Omega)^{-1}\|_\infty^{-2}\eta}{8\xi(l)}$ are all implied by taking $\zeta \leq \frac{e^{-4d_{\max}}\eta}{4\|S\|_\infty^4 \xi(l)}$, as a consequence of (61) (this also makes the assumption $\eta \leq 1$ verified). All of these inequalities are satisfied by choosing

$$\zeta = \frac{\epsilon' e^{-6d_{\max}}}{200\,\Delta^{3l}\|S\|_\infty^8} \frac{1 - e^{-2d_{\min}}}{1 - e^{-2d_{\max}}},$$

with $C'(S, d_{\max}) := 2\|S\|_\infty^4 e^{4d_{\max}}$. Now, we want ϵ' to be small enough so that $\|\hat{H} - H\|_\infty \leq \kappa/3$, where we compute $\hat{H}$ as in Equation 33 from $\mathrm{LI}_{\hat{\mathcal{N}}}(2\hat{V} - i\Omega)$. As in Remark 6.4 we can take $\epsilon' = \min\left(\kappa \frac{e^{-2d_{\max}}\|S\|_\infty^{-2}}{24\left(1+e^{4d_{\max}}\|S\|_\infty^4(1-e^{-2d_{\max}})\right)}, \frac{e^{-2d_{\max}}\|S\|_\infty^{-2}}{8}\right)$. Having ensured that $\|\hat{H} - H\|_\infty \leq \kappa/3$, we can learn the correct edges. By Lemma 3.1, the number of copies needed to obtain $|\hat{V}_{k,k'} - V_{k,k'}| \leq \zeta/2$ is

$$N \geq \frac{2^{16}(\|S\|_\infty^2 \frac{1}{1-e^{-2d_{\min}}} + \max_i |t_i|^2)^2 (1 + \max_i |t_i|)^2}{\zeta^2} \log\left(\frac{4m+1}{\delta}\right). \tag{57}$$

The theorem statement is then obtained by noting that by substituting $\|S\|_\infty$, $d_{\max}$ and $d_{\min}$ with their bounds the assumptions we checked to prove the theorem are still satisfied.

This concludes the analysis of the sample complexity. Let us now analyse the computational complexity of the algorithm. First, we iterate over all possible vertices and over all candidate neighborhoods of size $\xi(l)$. There are $\binom{m}{\xi(l)}$ of these. For each of these, we then iterate over all remaining subsets of size $\xi(l)$, of which there are $\binom{m-\xi(l)}{\xi(l)}$, and for each of them we need to invert a matrix of size $2\xi(l)$, which takes time $\mathcal{O}(\xi(l)^3)$. Then checking if the threshold condition is met takes time $\xi(l)^2$. We conclude that iterating over all possible neighborhoods and remaining candidates takes the time claimed in Equation (50), $\mathcal{O}\left(m\binom{m-\xi(l)}{\xi(l)}\binom{m}{\xi(l)}\xi(l)^3\right)$. It is then easy to check that the remaining steps only have a subleading order contribution. □

Remark 8.1. *For the sample complexity, assuming all relevant parameters of constant order except the threshold κ, we see that the number of samples required will be of order $\mathcal{O}(\kappa^{-2-\gamma}\log(m\delta^{-1}))$ for any $\gamma > 0$ and, crucially, is only logarithmic in the number of modes. Regarding the computational complexity, as in the worst case we have $\xi(l) \leq \Delta^l$ and given our bound on l, the worst-case computational complexity of our algorithm is polynomial in m with the exponent depending on single parameters, when the others are fixed, as $e^{O(\Delta^2 \log \Delta)}$, $e^{O(d_{\max})}$, $o\left(\frac{1}{d_{\min}^\gamma}\right)$, $o\left(\frac{1}{\kappa^\gamma}\right)$, $\forall\gamma > 0$. Again, assuming these parameters to be of constant order, we see that the graph learning algorithm runs in polynomial time.*

Remark 8.2. *As was the case for Hamiltonian learning, we can reduce the sample complexity of the graph learning protocol to scale like $\tilde{O}(\kappa^{-2})$ if we are willing to assume that the neighborhoods only grow polynomially in distance. However, this might be an assumption that is more difficult to verify or justify when the graph is unknown.*

9 Supplementary note 9: Symplectic diagonalization and bounds on singular values

In the following, we denote as $\sigma_{\max}(A)$ the maximum singular value of A (which coincides with the operator norm $\|A\|_\infty$) and as $\sigma_{\min}(A)$ the minimum singular value of A. The following fact is useful.

Lemma 9.1. *For any (symmetric) $2m \times 2m$ Hamiltonian $H > 0$, let a symplectic diagonalization be*

$$H = S^{-\intercal} D S^{-1}\,, \tag{58}$$

with $D = \oplus_{i=1}^{m} d_i I_{M_2}$, with $d_i > 0$, and $S \in \mathrm{Sp}(2m, \mathbb{R})$. Let us also denote as $d_{\min}$ and $d_{\max}$ the smallest and the largest symplectic eigenvalues of H, respectively. Then, the corresponding covariance matrix of the Gaussian state with Hamiltonian H is

$$V = S f(D) S^\intercal, \tag{59}$$

with $f(x) = \frac{1}{2}\coth(x) = \frac{1}{2}\frac{1+e^{-2x}}{1-e^{-2x}}$, and the following bounds hold for $t \in [0, \infty)$:

$$\left\| 2i\Omega V + \frac{t-1}{t+1} I \right\|_\infty \leq \|S\|_\infty^2 \frac{2}{1 - e^{-2d_{\min}}}, \tag{60}$$

$$\sigma_{\min}\left(2i\Omega V + \frac{t-1}{t+1} I \right) \geq \frac{2}{\|S\|_\infty^2} \frac{e^{-2d_{\max}}}{1 - e^{-2d_{\max}}}, \tag{61}$$

$$\sigma_{\min}\left(\frac{t}{t+1} \frac{2i\Omega V + I}{2i\Omega V - I} + \frac{1}{t+1} \right) \geq \frac{e^{-2d_{\max}}}{\|S\|_\infty^2}. \tag{62}$$

It is worth stressing that $\|S\|_\infty$ does not depend on the temperature and it is equal to 1 if there is no squeezing.

Proof. Equation 59 is a simple consequence of Equation 7. More precisely,

$$\begin{aligned} V &= \frac{i}{2}\Omega \coth(iH\Omega) \\ &= \frac{i\Omega}{2} \coth(iS^{-\intercal} D S^{-1} \Omega) \\ &= \frac{i\Omega}{2} \coth(iS^{-\intercal} D \Omega S^\intercal) \\ &= \frac{i\Omega}{2} S^{-\intercal} \coth(iD\Omega) S^\intercal \\ &= S \frac{i\Omega}{2} \coth(iD\Omega) S^\intercal\,. \end{aligned}$$

Let U be the matrix diagonalizing $iD\Omega$, i.e. $U(iD\Omega)U^{-1} = \bigoplus_j \operatorname{diag}(d_j, -d_j)$ and since coth is odd, we get

$$\coth(iD\Omega) = U^{-1} \bigoplus_j \coth(\operatorname{diag}(d_j, -d_j))U = U^{-1} \oplus_j \coth(d_j) \operatorname{diag}(1, -1)U = \bigoplus_j \coth(d_j) i \begin{pmatrix} 0 & 1 \\ -1 & 0 \end{pmatrix},$$

and hence

$$V = \frac{1}{2} S \bigoplus_i \coth(d_j) i^2 \begin{pmatrix} 0 & 1 \\ -1 & 0 \end{pmatrix}^2 S^{\intercal} = \frac{1}{2} S \coth(D) S^{\intercal} \equiv S f(D) S^{\intercal}.$$

As a consequence, we obtain that for any $t \geq 0$

$$2i\Omega V + \frac{t-1}{t+1} I = i\Omega \left(2V + \frac{t-1}{t+1} i\Omega\right) = i\Omega S \left(2f(D) + \frac{t-1}{t+1} i\Omega\right) S^{\intercal}. \tag{63}$$

Now, notice that $2f(D) + \frac{t-1}{t+1} i\Omega$ is Hermitian, and

$$\left(2f(D) + i\frac{t-1}{t+1}\Omega\right)^2 = \bigoplus_{i=1}^{m} \left(2f(d_i) - \frac{t-1}{t+1}\right)^2 |0_y\rangle\langle 0_y| + \left(2f(d_i) + \frac{t-1}{t+1}\right)^2 |1_y\rangle\langle 1_y|. \tag{64}$$

where $|0_y\rangle$ and $|1_y\rangle$ are the eigenvectors of the Pauli Y matrix. As a consequence, since $2f(d_i) \geq 1$, $\left|\frac{t-1}{t+1}\right| \leq 1$, and f is monotone decreasing, the largest eigenvalue is smaller than $(2f(d_{\min}) + 1)^2 = \left(\frac{2}{1-e^{-2d_{\min}}}\right)^2$ and the smallest eigenvalue is larger than $(2f(d_{\max}) - 1)^2 = \left(2\frac{e^{-2d_{\max}}}{1-e^{-2d_{\max}}}\right)^2$, that is:

$$\left(2\frac{e^{-2d_{\max}}}{1 - e^{-2d_{\max}}}\right)^2 I \leq \left(2f(D) + \frac{t-1}{t+1} i\Omega\right)^2 \leq \left(\frac{2}{1 - e^{-2d_{\min}}}\right)^2 I. \tag{65}$$

Hence the following operator inequalities hold:

$$\begin{aligned}
\left(2i\Omega V + \frac{t-1}{t+1} I\right)^{\dagger} \left(2i\Omega V + \frac{t-1}{t+1} I\right) &= S \left(2f(D) + \frac{t-1}{t+1} i\Omega\right) S^{\intercal} S \left(2f(D) + \frac{t-1}{t+1} i\Omega\right) S^{\intercal} \\
&\leq \|S\|_{\infty}^2 S \left(2f(D) + \frac{t-1}{t+1} i\Omega\right)^2 S^{\intercal} \\
&\leq \|S\|_{\infty}^2 \left(\frac{2}{1 - e^{-2d_{\min}}}\right)^2 S S^{\intercal} \\
&\leq \|S\|_{\infty}^4 \left(\frac{2}{1 - e^{-2d_{\min}}}\right)^2 I.
\end{aligned}$$

This proves Equation (60). Moreover, since S is symplectic, $\sigma_{\min}(S) = \sigma_{\max}^{-1}(S) = \|S\|_{\infty}^{-1}$, and $S^{\intercal} S \geq \sigma_{\min}^2(S) I = \|S\|_{\infty}^{-2} I$, and similarly $S S^{\intercal} \geq \|S\|_{\infty}^{-2} I$. Using this fact together with the lower bound in (65), we directly get that

$$\left(2i\Omega V + \frac{t-1}{t+1} I\right)^{\dagger} \left(2i\Omega V + \frac{t-1}{t+1} I\right) \geq \|S\|_{\infty}^{-4} \left(\frac{2e^{-2d_{\max}}}{1 - e^{-2d_{\max}}}\right)^2 I,$$

proving Equation (61). Finally, we consider

$$
\begin{aligned}
g(D) := (2f(D)+i\Omega)(2f(D)-i\Omega)^{-1} &= \bigoplus_{i=1}^{m} \frac{2f(d_i){-}1}{2f(d_i){+}1}|0_y\rangle\langle 0_y| + \frac{2f(d_i){+}1}{2f(d_i){-}1}|1_y\rangle\langle 1_y| \\
&= \bigoplus_{i=1}^{m} e^{-2d_i}|0_y\rangle\langle 0_y| + e^{2d_i}|1_y\rangle\langle 1_y|,
\end{aligned}
$$

from which it follows that $g(D) \geq 0$. Thus we have

$$
\begin{aligned}
\left(\frac{2i\Omega V+I}{2i\Omega V-I}\right) &= i\Omega S(2f(D)+i\Omega)S^{\intercal}S^{\intercal-1}(2f(D)-i\Omega)^{-1}S^{-1}i\Omega \\
&= i\Omega S(2f(D)+i\Omega)(2f(D)-i\Omega)^{-1}S^{-1}i\Omega \\
&= i\Omega S g(D) S^{-1} i\Omega,
\end{aligned}
$$

and hence

$$
\frac{t}{t+1}\frac{2i\Omega V+I}{2i\Omega V-I} + \frac{1}{t+1} = i\Omega S\left(\frac{tg(D)+I}{t+1}\right)S^{-1}i\Omega\,.
$$

It follows that

$$
\begin{aligned}
&\left(\frac{t}{t+1}\frac{2i\Omega V+I}{2i\Omega V-I} + \frac{1}{t+1}\right)^{\dagger}\left(\frac{t}{t+1}\frac{2i\Omega V+I}{2i\Omega V-I} + \frac{1}{t+1}\right) \\
&\qquad = i\Omega S^{-\intercal}\left(\frac{tg(D)+I}{t+1}\right)S^{\intercal}S\left(\frac{tg(D)+I}{t+1}\right)S^{-1}i\Omega \\
&\qquad \geq \|S\|_{\infty}^{-2} i\Omega S^{-\intercal}\left(\frac{tg(D)+I}{t+1}\right)^{2}S^{-1}i\Omega \\
&\qquad \geq \|S\|_{\infty}^{-2} e^{-4d_{\max}}(i\Omega)S^{-\intercal}S^{-1}(i\Omega) \\
&\qquad \geq \|S\|_{\infty}^{-4} e^{-4d_{\max}} I,
\end{aligned}
$$

proving Equation 62.

□

10 Supplementary note 10: Energy bounds

In this section we bound $\|S\|_\infty$, $d_{\min}$ and $\|t\|_2$ as a function of the energy of the state, i.e. the expectation value of the operator E_m as follows:

$$
\|t\|_2^2 \leq 2E, \tag{66}
$$

$$
\|S\|_\infty^2 \leq 4E, \tag{67}
$$

$$
(1-e^{-2d_{\min}})^{-1} \leq 8E^2. \tag{68}
$$

Indeed, for a Gaussian state ρ, the energy is

$$
E := \mathbb{E}[E_m] = \frac{\mathrm{tr}[V[\rho]]}{2} + \frac{\|t[\rho]\|_2^2}{2}. \tag{69}
$$

Via the relation in Equation (59), i.e.

$$V = Sf(D)S^{\intercal}, \tag{70}$$

with $f(x) = \frac{1}{2}\coth(x) = \frac{1}{2}\frac{1+e^{-2x}}{1-e^{-2x}} \geq \frac{(1-e^{-2x})^{-1}}{2} \geq \frac{1}{2}$, we also have

$$\operatorname{tr}[V] \geq \operatorname{tr}[Sf(D)S^{\intercal}] \geq \operatorname{tr}[SS^{\intercal}]/2 \geq \|S\|_{\infty}^{2}/2, \tag{71}$$

$$\operatorname{tr}[V] \geq \operatorname{tr}[Sf(D)S^{\intercal}] = \operatorname{tr}[SS^{\intercal}f(D)] \geq \|S^{-1}\|_{\infty}^{-2}\operatorname{tr}[f(D)] \geq \|S\|_{\infty}^{-2}(1-e^{-2d_{\min}})^{-1}, \tag{72}$$

where in the first derivation we used $f(x) \geq 1/2$ and in the second that $\|S\|_{\infty} = \|S^{-1}\|_{\infty}$ for symplectic matrices. This also implies

$$(1-e^{-2d_{\min}})^{-1} \leq 8E^{2}. \tag{73}$$

11 Supplementary note 11: Family of Hamiltonians with condition number scaling with the size of the graph

The following example shows why one should expect a dependence on the condition number for results with sample complexity logarithmic in the number of modes, when additive error guarantees are used. The precision matrices of size $m \times m$

$$H_m = \begin{pmatrix} 1 & -1 & 0 & \cdots & 0 & 0 \\ -1 & 2 & -1 & \cdots & 0 & 0 \\ 0 & -1 & 2 & \cdots & 0 & 0 \\ \vdots & \vdots & \vdots & \ddots & \vdots & \vdots \\ 0 & 0 & 0 & \cdots & 2 & -1 \\ 0 & 0 & 0 & \cdots & -1 & 2 \end{pmatrix}$$

have inverses

$$H_m^{-1} = \begin{pmatrix} m & m-1 & m-2 & \cdots & 2 & 1 \\ m-1 & m-1 & m-2 & \cdots & 2 & 1 \\ m-2 & m-2 & m-2 & \cdots & 2 & 1 \\ \vdots & \vdots & \vdots & \ddots & \vdots & \vdots \\ 2 & 2 & 2 & \cdots & 2 & 1 \\ 1 & 1 & 1 & \cdots & 1 & 1 \end{pmatrix},$$

meaning that to learn the entries of the covariance matrix with additive error, one expects that poly(m) samples are needed, as the variance of the random variables is polynomial in m. Thus, already in the classical case, estimating the entries of the covariance matrix to constant precision requires polynomial samples, so that with logarithmic number of samples we do not expect to be able to estimate the entries of the Hamiltonian to constant precision, even if its entries are all of constant order. Furthermore, the condition number of H_m, and thus the norm of H_m^{-1}, is of order m^2.

References

[1] Holevo, A. S. *Quantum systems, channels, information: a mathematical introduction*, vol. 16 (Walter de Gruyter, 2012).

[2] Serafini, A. *Quantum continuous variables: a primer of theoretical methods* (CRC press, 2017).

[3] Bhatia, R. & Jain, T. On symplectic eigenvalues of positive definite matrices. *Journal of Mathematical Physics* **56**, 112201 (2015). URL https://doi.org/10.1063/1.4935852. https://pubs.aip.org/aip/jmp/article-pdf/doi/10.1063/1.4935852/15736053/112201_1_online.pdf.

[4] Lewenstein, M., Sanpera, A. & Ahufinger, V. *Ultracold Atoms in Optical Lattices: Simulating quantum many-body systems* (Oxford University Press, Oxford, New York, 2012).

[5] Jaksch, D., Bruder, C., Cirac, J. I., Gardiner, C. W. & Zoller, P. Cold Bosonic Atoms in Optical Lattices. *Physical Review Letters* **81**, 3108–3111 (1998). URL https://link.aps.org/doi/10.1103/PhysRevLett.81.3108. Publisher: American Physical Society.

[6] Peano, V., Brendel, C., Schmidt, M. & Marquardt, F. Topological Phases of Sound and Light. *Physical Review X* **5**, 031011 (2015). URL http://arxiv.org/abs/1409.5375. ArXiv:1409.5375 [cond-mat].

[7] Carusotto, I. *et al.* Photonic materials in circuit quantum electrodynamics. *Nature Physics* **16**, 268–279 (2020). URL https://www.nature.com/articles/s41567-020-0815-y. Publisher: Nature Publishing Group.

[8] Periwal, A. *et al.* Programmable interactions and emergent geometry in an array of atom clouds. *Nature* **600**, 630–635 (2021). URL https://www.nature.com/articles/s41586-021-04156-0. Publisher: Nature Publishing Group.

[9] Senanian, A., Wright, L. G., Wade, P. F., Doyle, H. K. & McMahon, P. L. Programmable large-scale simulation of bosonic transport in optical synthetic frequency lattices. *Nature Physics* **19**, 1333–1339 (2023). URL https://www.nature.com/articles/s41567-023-02075-7. Publisher: Nature Publishing Group.

[10] Youssefi, A. *et al.* Topological lattices realized in superconducting circuit optomechanics. *Nature* **612**, 666–672 (2022). URL https://www.nature.com/articles/s41586-022-05367-9. Publisher: Nature Publishing Group.

[11] Katz, O. & Monroe, C. Programmable Quantum Simulations of Bosonic Systems with Trapped Ions. *Physical Review Letters* **131**, 033604 (2023). URL https://link.aps.org/doi/10.1103/PhysRevLett.131.033604. Publisher: American Physical Society.

[12] Nichols, R., Liuzzo-Scorpo, P., Knott, P. A. & Adesso, G. Multiparameter Gaussian Quantum Metrology. *Physical Review A* **98**, 012114 (2018). URL http://arxiv.org/abs/1711.09132. ArXiv:1711.09132 [quant-ph].

[13] Montenegro, V. *et al.* Review: Quantum metrology and sensing with many-body systems. *Physics Reports* **1134**, 1–62 (2025). URL https://www.sciencedirect.com/science/article/pii/S0370157325001565.

[14] Purdy, T. P. *et al.* Optomechanical raman-ratio thermometry. *Physical Review A* **92**, 031802 (2015). URL http://dx.doi.org/10.1103/PhysRevA.92.031802.

[15] Cenni, M. F. B., Lami, L., Acín, A. & Mehboudi, M. Thermometry of gaussian quantum systems using gaussian measurements. *Quantum* **6**, 743 (2022). URL http://dx.doi.org/10.22331/q-2022-06-23-743.

[16] Davies, E. B. Markovian master equations. *Communications in Mathematical Physics* **39**, 91–110 (1974). URL https://doi.org/10.1007/BF01608389.

[17] Scandi, M. & Alhambra, A. M. Thermalization in open many-body systems and KMS detailed balance (2025). URL http://arxiv.org/abs/2505.20064. ArXiv:2505.20064 [quant-ph].

[18] Shiraishi, K., Nakagawa, M. & Mori, T. Davies equation without the secular approximation: Reconciling locality with quantum thermodynamics for open quadratic systems (2025). URL http://arxiv.org/abs/2507.10080. ArXiv:2507.10080 [quant-ph].

[19] Gluza, M., Eisert, J. & Farrelly, T. Equilibration towards generalized Gibbs ensembles in non-interacting theories. *SciPost Physics* **7**, 038 (2019). URL https://scipost.org/SciPostPhys.7.3.038.

[20] Hastie, T., Tibshirani, R. & Friedman, J. *The Elements of Statistical Learning*. Springer Series in Statistics (Springer, New York, NY, 2009). URL http://link.springer.com/10.1007/978-0-387-84858-7.

[21] Hastie, T., Tibshirani, R. & Wainwright, M. *Statistical Learning with Sparsity: The Lasso and Generalizations* (Chapman and Hall/CRC, New York, 2015).

[22] Wainwright, M. J. *High-Dimensional Statistics: A Non-Asymptotic Viewpoint*. Cambridge Series in Statistical and Probabilistic Mathematics (Cambridge University Press, Cambridge, 2019). URL https://www.cambridge.org/core/books/highdimensional-statistics/8A91ECEEC38F46DAB53E9FF8757C7A4E.

[23] Wang, W., Wainwright, M. J. & Ramchandran, K. Information-theoretic bounds on model selection for gaussian markov random fields. In *2010 IEEE International Symposium on Information Theory*, 1373–1377 (2010).

[24] Friedman, J., Hastie, T. & Tibshirani, R. Sparse inverse covariance estimation with the graphical lasso. *Biostatistics* **9**, 432–441 (2008).

[25] Yuan, M. & Lin, Y. Model Selection and Estimation in the Gaussian Graphical Model. *Biometrika* **94**, 19–35 (2007). URL https://www.jstor.org/stable/20441351. Publisher: [Oxford University Press, Biometrika Trust].

[26] Ravikumar, P., Wainwright, M. J., Raskutti, G. & Yu, B. High-dimensional covariance estimation by minimizing ℓ_1-penalized log-determinant divergence. *Electronic Journal of Statistics* **5**, 935–980 (2011). URL https://projecteuclid.org/journals/electronic-journal-of-statistics/volume-5/issue-none/High-dimensional-covariance-estimation-by-minimizing-%e2%84%931-penalized-log-determinant/10.1214/11-EJS631.full. Publisher: Institute of Mathematical Statistics and Bernoulli Society.

[27] Meinshausen, N. & Bühlmann, P. High-dimensional graphs and variable selection with the Lasso. *The Annals of Statistics* **34**, 1436–1462 (2006). URL https://projecteuclid.org/journals/annals-of-statistics/volume-34/issue-3/High-dimensional-graphs-and-variable-selection-with-the-Lasso/10.1214/009053606000000281.full. Publisher: Institute of Mathematical Statistics.

[28] Yuan, M. High Dimensional Inverse Covariance Matrix Estimation via Linear Programming. *Journal of Machine Learning Research* **11**, 2261–2286 (2010). URL http://jmlr.org/papers/v11/yuan10b.html.

[29] Cai, T., Liu, W. & Luo, X. A Constrained ℓ_1 Minimization Approach to Sparse Precision Matrix Estimation. *Journal of the American Statistical Association* **106**, 594–607 (2011). URL https://doi.org/10.1198/jasa.2011.tm10155. Publisher: ASA Website _eprint: https://doi.org/10.1198/jasa.2011.tm10155.

[30] Zhang, T. & Zou, H. Sparse precision matrix estimation via lasso penalized d-trace loss. *Biometrika* **101**, 103–120 (2014).

[31] Wang, L., Ren, X. & Gu, Q. Precision matrix estimation in high dimensional gaussian graphical models with faster rates. In *Artificial Intelligence and Statistics*, 177–185 (PMLR, 2016).

[32] Misra, S., Vuffray, M. & Lokhov, A. Y. Information theoretic optimal learning of gaussian graphical models. In Abernethy, J. & Agarwal, S. (eds.) *Proceedings of Thirty Third Conference on Learning Theory*, vol. 125 of *Proceedings of Machine Learning Research*, 2888–2909 (PMLR, 2020). URL https://proceedings.mlr.press/v125/misra20a.html.

[33] Kelner, J., Koehler, F., Meka, R. & Moitra, A. Learning some popular gaussian graphical models without condition number bounds. *Advances in Neural Information Processing Systems* **33**, 10986–10998 (2020).

[34] Boucheron, S., Lugosi, G. & Bousquet, O. *Concentration Inequalities*, 208–240 (Springer Berlin Heidelberg, 2004). URL http://dx.doi.org/10.1007/978-3-540-28650-9_9.

[35] Weedbrook, C. *et al.* Gaussian quantum information. *Reviews of Modern Physics* **84**, 621–669 (2012). URL http://dx.doi.org/10.1103/RevModPhys.84.621.

[36] Mele, F. A. *et al.* Learning quantum states of continuous-variable systems. *Nature Physics* **21**, 2002–2008 (2025). URL http://dx.doi.org/10.1038/s41567-025-03086-2.

[37] Anshu, A., Arunachalam, S., Kuwahara, T. & Soleimanifar, M. Sample-efficient learning of interacting quantum systems. *Nature Physics* **17**, 931–935 (2021). URL https://doi.org/10.1038/s41567-021-01232-0.

[38] Hoorfar, A. & Hassani, M. Inequalities on the lambert w function and hyperpower function. *J. Inequal. Pure and Appl. Math* **9**, Paper No. 51 (2008).

[39] Cramer, M. & Eisert, J. Correlations, spectral gap and entanglement in harmonic quantum systems on generic lattices. *New Journal of Physics* **8**, 71 (2006). URL https://dx.doi.org/10.1088/1367-2630/8/5/071.

[40] Schuch, N., Cirac, J. I. & Wolf, M. M. Quantum states on harmonic lattices. *Communications in Mathematical Physics* **267**, 65–92 (2006). URL https://doi.org/10.1007%2Fs00220-006-0049-6.
