# Supplementary material for: Efficient Hamiltonian, structure and trace distance learning of Gaussian states
Source: Nat Commun. 2026 Sep 16;17:10898. doi: 10.1038/s41467-026-77405-3 (PMC13677717; doi:10.1038/s41467-026-77405-3)
Supplement: Supplementary file 2 [file 41467_2026_77405_MOESM2_ESM.pdf]

nature portfolio

Peer Review File

Efficient Hamiltonian, structure and trace distance learning of Gaussian states

Corresponding Author: Dr Marco Fanizza

This file contains all reviewer reports in order by version, followed by all author rebuttals in order by version.

Version 0:

Reviewer comments:

Reviewer #1

(Remarks to the Author)
This manuscript investigates Gaussian state learning with a special focus on learning its corresponding Hamiltonian. Fruitful results are obtained: (1) Sample complexity upper bound on learning the covariance matrix and the mean vector of Gaussian states; (2) Sample complexity upper bound on learning Gaussian states in trace distance; (3) Sample complexity upper bound on learning state Hamiltonians in trace distance; (4) Sample complexity upper bound on learning the graph of Hamiltonians. Result (1) is the foundation of Results (2,3,4). Two remarkable achievements include the epsilon^(-2) scaling in Gaussian state learning, the logarithmic sample complexity of the number of modes in learning sparse Hamiltonian. Although the results are interesting, I have several concerns on this manuscript. One of my major concerns—as detailed below—is this paper is presented in a TCS (theoretical computer science) form and is not tailored to the audience of Nature Communications.

Major comments:
(1) The motivation behind Hamiltonian learning in Gaussian state is unclear. From the manuscript, it is even unclear why the Hamiltonian defined for bosonic systems here should be called “Hamiltonian” as an analogy of the one defined for spin systems. First, if a quantum system is subject to some bosonic Hamiltonian, will it (in regular circumstances) thermalize into a Gaussian state defined by this Hamiltonian, similar to spin systems where quantum states will thermalize to Gibbs states defined by a given Hamiltonian? If not, then learning Hamiltonian from Gaussian states does not seem to have much practical relevance. Second, does sparse Hamiltonian often appear in nature or in experiments? For example, for the purpose of sensing, what kind of useful physical quantities can we learn by learning the Hamiltonian? For the purpose of quantum computing, is the goal here to benchmark Hamiltonians used for continuous variable quantum computing? How to justify sparse Hamiltonian is a cornerstone of continuous variable quantum computing? The current framework does not extend to non-Gaussian states (as a comparison, local Hamiltonians for qubit systems are universal.) Overall, since sparse bosonic Hamiltonians were not extensively studied before like for discrete-variable systems, it is important for the authors to lay out the motivations behind this research. Providing motivations solely from a perspective of TCS is not sufficient for publication in Nature Communications.
(2) Section 2.1 does not review any previous classical results in detail. It was only vaguely mentioned “From that, one is also usually interested in estimating the parameters of Θ, while having a scaling of the sample complexity that only scales logarithmically with the dimension of the random variables.” What was the exact sample complexity achieved? What was the algorithm and was it efficient? Since the argument here is a classically useful technique—conditional independence—is not useful any more in the quantum case, will the local inversion technique introduced here for quantum also be useful for the classical case? Does it lead to the same result (derived using conditional independence)? If not, which parameter is not as good?
(3) The authors should clarify the differences between spin Hamiltonian and Gaussian bosonic Hamiltonian. Here, within the Gaussian framework, every operator can be efficiently represented using a 2m*2m matrix that scale only linearly in m, but for discrete variables, the size of the Gibbs state scales exponentially with respect to the number of qubits. Therefore, it seems less challenging to achieve the logarithmic sample complexity and the polynomial computation complexity in the Gaussian framework than in spin systems (as reviewed in the introduction part). Also, it seems that a fair comparison can only be made between Gaussian state learning and stabilizer state learning, instead of general local Hamiltonian learning.
(4) There are numerous parameters/notations introduced in this manuscript. It is difficult for readers to immediately remember the meanings of each parameter when they appear and it takes a lot of time to find out each definition given that there are so many parameters. A table that shows the notations, the definitions, and maybe where they appear can hugely improve the

clarity. Furthermore, a comparison between the dependence of sample complexity on these parameters to previous results is lacking.
(5) There is no discussion on lower bounds. For example, 1/epsilon^2 scaling is usually optimal. Is this also the case here? Does the benefit of 1/epsilon^2 introduce some side effects, like making dependence on other parameters significantly worse? Also, are the logarithmic sample complexity and the polynomial computation complexity optimal?

Other comments:

(1) The result section lacks a discussion of computational complexity—although it was mentioned multiple times in introduction. Even in the Methods section, the details of computational efficiency are buried in the proofs of sample complexity. Clear algorithms should be given.
(2) The presentation of Fig 1 is quite confusing. There are four colors. What do they represent respectively? For example, what are the differences between the yellow and the red squares in the second step? (A figure has to be self-explanatory, but here readers cannot understand its meaning unless after reading Section 4.) Maybe a drawing of an interaction graph can improve the clarity.
(3) The definition of Omega before Eq.(1) is confusing. First, the notation diag() was not explained. Second, It was said "where all blocks are $m \times m$ matrices", shouldn't it be 2×2?
(4) In Definition 4.8, the first sentence right below the equation should be "The Gaussian graph learning problem …"

Reviewer #2

(Remarks to the Author)
I co-reviewed this manuscript with one of the reviewers who provided the listed reports. This is part of the Nature Communications initiative to facilitate training in peer review and to provide appropriate recognition for Early Career Researchers who co-review manuscripts.

Reviewer #3

(Remarks to the Author)
This paper addresses three core learning problems for Gaussian states: inferring Hamiltonian parameters, learning the interaction graph structure, and learning the full quantum state in trace distance, all using experimentally friendly heterodyne measurements. The central achievement is the "local inversion technique," which allows for estimating the Hamiltonian from local data, leading to a breakthrough sample complexity that scales only logarithmically with the system size for Hamiltonian and graph learning. Furthermore, the work provides the first trace distance learning results for these systems with an efficient quadratic scaling in precision, under some state restrictions. The authors also develop new, independently valuable continuity bounds relating Hamiltonians and covariance matrices, underpinning their learning protocols.
While the paper introduces promising learning protocols with favorable scaling in system size, the practical efficiency of these methods can be significantly impacted by a strong dependence on other system parameters, potentially leading to very high sample or computational costs for general cases or when high precision is required. The conditions under which the algorithms are truly "efficient" are quite specific and may not hold broadly without further refinements. As it stands, this contribution appears to be a technical, theoretical advance of interest primarily to the Hamiltonian-learning theory community. In the absence of additional evidence or empirical benchmarks demonstrating its wider applicability, I cannot recommend acceptance at this time.
Here are some of our questions:
1. The sample complexities for Hamiltonian and graph learning also exhibit, in some cases, a very steep dependence on other physical parameters like the bounds on symplectic eigenvalues ($d_{\min}$, $d_{\max}$), squeezing ($\|S\|_\infty$), and graph degree (Δ). For instance, Corollary 6.1 shows a dependency involving $\text{poly}\{\Delta^{\Delta\beta_{\max}}(\dots)^{\ln \Delta}\}$, and the number of samples N in Theorem 7.1 for graph learning scales with ζ^{-2}, where ζ itself has a complex and potentially very small value depending exponentially on l (which in turn depends on Δ, $\beta_{\max}$) and other parameters. The authors acknowledge the dependence on $d_{\min}$ (related to a condition number) and discuss its classical precedents. However, the practical utility of the "efficient" label hinges on these other parameters not leading to prohibitively large prefactors. If Δ or $d_{\max}$ are large, the actual number of samples required could become immense, limiting the applicability in some physical regimes despite the favorable $\ln(m)$ scaling. The paper assumes "constant-size bounds on the relevant parameters" for its main claims, but the impact of these "constants" being large warrants careful consideration for practical implementation.
2. The Learn Graph Gaussian algorithm (Algorithm 1) has a stated computational complexity of $\mathcal{O}\left(m\binom{m}{\xi(l)}\binom{m-\xi(l)}{\xi(l)}\xi(l)^3\right)$. The parameter l (local neighborhood extent) is chosen based on Δ, $d_{\max}$, and the target precision κ, potentially scaling as $l = \mathcal{O}(\Delta^2 d_{\max} + \ln(1/\kappa))$. The neighborhood size $\xi(l)$ can grow as Δ^l. The authors state this is polynomial "assuming these parameters [$\Delta, d_{\max}$] to be of constant order". However, if Δ is not a very small constant, or if $d_{\max}$ is large, l can become significant. If $\xi(l)$ is not a small constant (independent of m), the binomial term $\binom{m}{\xi(l)}$ can render the algorithm super-polynomial in m. For the algorithm to be truly polynomial-time in m for general graphs, $\xi(l)$ must be $\mathcal{O}(\log m / \log\log m)$ at best, or more strictly, a constant. The conditions for $\xi(l)$ to be this small are quite restrictive (requiring very small Δ and l). This is a notable limitation for the practical efficiency of graph learning on more general or densely connected local neighborhoods.
3. The sample complexity for Hamiltonian learning scales as $\epsilon^{-(2+4\ln \Delta)}$ and for graph learning as $\kappa^{-(2+6\ln \Delta)}$ for general graphs. The authors acknowledge this is "not the expected ϵ^{-2} (or κ^{-2})" and identify it as a potential limitation of their Taylor expansion-based approach for the general case. While acknowledged, this is a significant point because for graphs with even moderate degree Δ, the exponent on the

precision can become substantially larger than 2 (e.g., if $\ln \Delta \approx 1$, the exponent is ≈ 8). This would make achieving high precision very costly in terms of samples, more so than typical learning algorithms. This is an important trade-off for achieving the $\ln m$ scaling.
4. To underscore the practical relevance and broader appeal of this work to the quantum information and physics communities, we recommend that the authors include performance benchmarks—such as explicit sample-complexity improvements—for experimentally relevant bosonic Gaussian states. Moreover, it remains unclear whether the proposed protocol maintains its advantages in the presence of realistic heterodyne-measurement imperfections and finite-sample statistical fluctuations; an analysis or empirical evaluation of its robustness under these conditions would greatly strengthen the manuscript.

Reviewer #4

(Remarks to the Author)
I co-reviewed this manuscript with one of the reviewers who provided the listed reports. This is part of the Nature Communications initiative to facilitate training in peer review and to provide appropriate recognition for Early Career Researchers who co-review manuscripts.

Reviewer #5

(Remarks to the Author)
Overall this is an interesting paper addressing an interesting problem in quantum learning. The results are technically sound and the main ideas of the paper are well explained.
The relative importance of this result seem to be overstated in some parts of the paper and relation to recent literature is not very well explained. We have the following questions/comments about the paper that I hope the authors can answer:

1. In the abstract the statement: "Taken together, our results put the status of the quantum
Hamiltonian learning problem for continuous variable systems in a more advanced state when
compared to spins, where state-of-the-art results are either unavailable or quantitatively inferior
to ours." should be removed. The quadratic continuous variable case considered here is restricted compared to the more general local interactions considered in Baksi et.al. for the discrete case. To make this statement fair, the learning task here must be extended beyond quadratic Hamiltonians.

2. The authors briefly mention the difference between this work and other recent results [MMB+24, Hol24] [BMM+24]. This has to be made more explicit. For instance, what extra assumptions are made in this work compared to [MMB+24]? A table showing the results and assumptions of this paper against the same from other works would be ideal for this.

3.What are the computational complexities of the learning tasks solved in Them 3.1 - 3.3 in terms of the problem parameters?

4. \hat{H} seems to be arbitrarily introduced before Eq 20. Readers will find it easier if the computation of \hat{H} is briefly explained at this point. Including the local inversion + patching algorithm as a box somewhere in the beginning of the paper will also improve readability of the paper.

5. Is the epsilon in (18) the same as (20)?

6. In Theorem 4.1, what is the significance of the assumed upper bound on the operator norm between V and \hat{V}? Is this assumption restrictive? Does it impose additional constraints on the precision \epsilon in the same theorem? Our main concern here is the following scenario: if this upper bound is too close to zero, this forces the precision to be artificially small, effectively increasing the sample complexity of some problems. This concern can be alleviated if it can be shown that the upper bound is system size independent.

7. How does the operator norm of S scale for physically relevant systems (local interactions, bounded interaction strength)?

8. In the proof of lemma 4.6. What do the authors mean when they write $E E^\dagger \leq (...)$ Is it supposed to mean that RHS -LHS is a PSD matrix. If so, please clarify this in the text. May be different notation (curly inequalities) would be helpful for denoting matrix inequalities.

9. In section 4.3, can the sparse approximation be improved by using another polynomial basis. For instance, using a Chebyshev expansion instead of a Taylor expansion in Lemma 4.6?

Version 1:

Reviewer comments:

Reviewer #1

(Remarks to the Author)

In my previous round of report, I raised concerns about the clarity of presentation of the manuscript, which was quite technical and not tailored to Nature Communications. I also asked questions about the physical motivation for this problem, comparison with related classical and quantum literature, and matching lower bounds for their results.
In the resubmission, the authors provide a detailed and comprehensive answer to most of my questions. In particular, they discussed the relevance of sparse quadratic bosonic Hamiltonian in a lot of physics platforms, both in theory and in experiments. They also provide a more detailed and explicit review of the sparse Gaussian learning problem in the classical literature, and discussion on the counter part of their problem in spin systems. The added figures and tables the also improve the readability. The structure of the revised manuscript also looks more friendly to the physics audience at Nature Communications.
Since most of my concerns from the previous round have been addressed, I am now happy to recommend publication of this manuscript.

Reviewer #3

(Remarks to the Author)
The authors have thoroughly addressed the concerns from the first round. The new sections on physical motivation (Section 2.1) and classical Gaussian graphical models (Section 2.2) substantially improve the paper's accessibility and contextualization. The numerical simulations (Figures 4-5) are a useful addition, demonstrating mode-independent reconstruction error with realistic sample sizes and a clear advantage of local over global inversion. The notation table, improved figures, and refined analysis of the neighborhood size (Lemma 4.11) further strengthen the manuscript.

The paper makes a novel contribution by opening a natural and important research direction. The local inversion technique elegantly addresses the key quantum-specific challenge: the lack of conditional independence that makes classical approaches work with nearest-neighbor information alone. The insight that correlations in the inverse of the covariance matrix decay fast enough for local recovery is non-trivial and of independent interest. The authors are clear about current limitations (parameter dependence beyond system size, absence of matching lower bounds, restriction to positive-temperature states for trace distance). These are natural targets for follow-up work.

Recommendation: accept. The revision has comprehensively addressed the previous reviewers' concerns.

Reviewer #4

(Remarks to the Author)
I co-reviewed this manuscript with one of the reviewers who provided the listed reports. This is part of the Nature Communications initiative to facilitate training in peer review and to provide appropriate recognition for Early Career Researchers who co-review manuscripts.

Reviewer #5

(Remarks to the Author)
We thank the authors for addressing our comments and recommend the paper for publication after the following minor points are addressed.

1. We still think that a comparison table between this work and [MMB+25]/[BMM+24]/ will be valuable in the final version and should be included. It should compare: setting/task (Hamiltonian / graph / trace-distance learning), measurement model (heterodyne, etc.), assumptions (known graph? bounded degree?), sample complexity scaling (in m, epsilon and other key parameters), computational scaling.

2. Between Eq(9) and (10) the authors define E_m as the total energy. This is confusing as this is not the Hamiltonian of the system under consideration. It would be more natural here to connect it to particle number as mentioned by the authors in their reply to our earlier question about \| S \|_\infty.

3. In (17), relation between N and \| S \|_infty, d_max etc. should be stated in loose terms as in (15). Given that there can be implicit relations between these quantities and the size of the system in some cases, it is only fair that it should be stated in the informal theorem statement.

Open Access This Peer Review File is licensed under a Creative Commons Attribution 4.0 International License, which permits use, sharing, adaptation, distribution and reproduction in any medium or format, as long as you give appropriate credit to the original author(s) and the source, provide a link to the Creative Commons license, and indicate if changes were made.
In cases where reviewers are anonymous, credit should be given to 'Anonymous Referee' and the source.
The images or other third party material in this Peer Review File are included in the article's Creative Commons license, unless indicated otherwise in a credit line to the material. If material is not included in the article's Creative Commons license and your intended use is not permitted by statutory regulation or exceeds the permitted use, you will need to obtain permission directly from the copyright holder.
To view a copy of this license, visit https://creativecommons.org/licenses/by/4.0/

We thank the referees for their constructive comments. In response, we have made several revisions throughout the manuscript. Most importantly:

- We have improved the presentation of the problem and of the algorithms adding new figures, tables and remarks.
- We have expanded the discussions on the physical relevance of the problem, especially in relation to recent papers implementing bosonic sparse Hamiltonians experimentally.
- We clarified the relation with classical algorithms and gave a more detailed account of the classical results.
- We have generated numerics in a couple of simple cases, that show that our proposal for Hamiltonian learning based on local inversion can be indeed practical and improve with respect to a naive plug-in estimation method.
- We have refined the analysis of the scaling of the number of samples with the precision: the length scale of the reconstruction can in fact be taken as $l \sim \frac{\log(\epsilon^{-1})}{\log\log(\epsilon^{-1})}$ rather than $\log\left(\epsilon^{-1}\right)$ as previously stated. This implies that the correction to ϵ^{-2} in the sample complexity are only subpolynomial in ϵ^{-1}.

We also corrected typographical errors and minor calculation mistakes, not affecting the results. Together with the new version, we provide pointers to major changes in the manuscript, and a document generated using latexdiff.

Major changes:

- Figure 1 explains the relation between the graph and the sparsity pattern of the Hamiltonian matrix. The color code for Figure 2 is now explained. The algorithm for Hamiltonian learning is now summarized in Figure 3.
- Table 1, summarizing the notation and interpretation of the parameters. Physical interpretations of the parameters are now discussed in the notation section.
- Discussion about the physical relevance of our study: connections with analog simulations of sparse bosonic Hamiltonians and recent experimental breakthroughs, and with metrology. Discussion of the Gibbs input assumption (Section 2.1).
- Extended background section on the classical literature on precision matrix estimation (Section 2.2).
- Numerics for Hamiltonian learning are now displayed in Figure 4 and 5.
- Discussion of robustness with respect to noise (Remark 4.1)
- The computational complexity is discussed in the main result section after the sample complexity theorems.
- Comparison to bounds in [MMB$^+$25] in terms of energy and purity parameters: see discussion after Theorem 3.1, Remark 4.3 and Appendix B for energy bounds for parameters $\|S\|_\infty$ and $d_{\min}$.
- The sample complexity statements and proofs have been modified as a consequence of the improved analysis in Lemma 4.11 on the size of the neighborhood sufficient for the reconstruction.

Below, we provide a detailed point-by-point reply to the referees' remarks.

Kind Regards,

Marco Fanizza, Cambyse Rouzé, Daniel Stilck França

Reply to Reviewer 1

This manuscript investigates Gaussian state learning with a special focus on learning its corresponding Hamiltonian. Fruitful results are obtained: (1) Sample complexity upper bound on learning the covariance matrix and the mean vector of Gaussian states; (2) Sample complexity upper bound on learning Gaussian states in trace distance; (3) Sample complexity upper bound on learning state Hamiltonians in trace distance; (4) Sample complexity upper bound on learning the graph of Hamiltonians. Result (1) is the foundation of Results (2,3,4). Two remarkable achievements include the $epsilon^{-2}$ *scaling in Gaussian state learning, the logarithmic sample complexity of the number of modes in learning sparse Hamiltonian. Although the results are interesting, I have several concerns on this manuscript. One of my major concerns—as detailed below—is this paper is presented in a TCS (theoretical computer science) form and is not tailored to the audience of Nature Communications.*

Reply. We thank the referee for their assessment. We have followed their suggestions for improving the presentation of the results to target the audience of Nature Communication, as we summarized in the reply to the editor above, and as we explain in detail in the following.

Major comments: (1) The motivation behind Hamiltonian learning in Gaussian state is unclear. From the manuscript, it is even unclear why the Hamiltonian defined for bosonic systems here should be called "Hamiltonian" as an analogy of the one defined for spin systems. First, if a quantum system is subject to some bosonic Hamiltonian, will it (in regular circumstances) thermalize into a Gaussian state defined by this Hamiltonian, similar to spin systems where quantum states will thermalize to Gibbs states defined by a given Hamiltonian? If not, then learning Hamiltonian from Gaussian states does not seem to have much practical relevance. Second, does sparse Hamiltonian often appear in nature or in experiments? For example, for the purpose of sensing, what kind of useful physical quantities can we learn by learning the Hamiltonian? For the purpose of quantum computing, is the goal here to benchmark Hamiltonians used for continuous variable quantum computing? How to justify sparse Hamiltonian is a cornerstone of continuous variable quantum computing? The current framework does not extend to non-Gaussian states (as a comparison, local Hamiltonians for qubit systems are universal.) Overall, since sparse bosonic Hamiltonians were not extensively studied before like for discrete-variable systems, it is important for the authors to lay out the motivations behind this research. Providing motivations solely from a perspective of TCS is not sufficient for publication in Nature Communications.

Reply. We thank the referee for raising important concerns on the experimental relevance of the problem we consider. We have added Section 2.1 adapting the following reply (some explanations in the reply are omitted in the text since they are too detailed). Bosonic Hamiltonians with finite number of modes are used to describe a multitude of interacting many-body systems, such as cold atoms in optical lattices, magnetization fields, optomechanical systems, trapped ions, and the electromagnetic field in cavities, interacting with matter [Ser17]. Among those, sparse Hamiltonian have been extensively considered for bosonic systems, as most materials are naturally described by Hamiltonians with a lattice structure, where the interactions strength decays with the distance. The sparse Hamiltonians considered in this work are models for where the interactions are assumed to be zero if vertices are not connected, for example because the corresponding sites are further than some fixed distance on a lattice. This includes the approximation of considering non-zero interactions only between first neighbors. A key example of this is a grid of harmonic oscillators, an idealization which is a cornerstone of theoretical physics, since any potential can be approximated around its

minimum by a quadratic one. In systems with higher order-interactions, the Bogoliubov-De Gennes approach is used to study excitations above the ground state, via an effective quadratic Hamiltonian. This method captures the physics of weakly-interacting dilute Bose gases [LSA+12]. Moreover, a typical quadratic Hamiltonian is also the hopping (or tight-binding) Hamiltonian on a graph, which describes particle-number-preserving transport and is the basis of all lattice models. The sharp cutoff on local interactions is quite convenient for learning questions, as it reduces the number of parameters. Considering rapidly decaying interactions would be feasible in our framework but not clearly illuminating more with respect to using a hard-cutoff.

In fact, experimentally realizing local (and thus sparse) Hamiltonians, including bosonic ones, is the goal of quantum simulation [LSA+12], both for reproducing models of condensed matter physics and for creating and studying meta-materials that cannot be found in nature. Starting from proposals for quantum simulation of the Bose-Hubbard model in cold atoms [JBC+98], the field is now very mature and exploring also Hamiltonian with non-Euclidean interaction graphs, which exhibiting non-trivial band structure due to their topology [PBSM15, CHK+20].

With current technology, it is in fact possible to realize simulators that implement Hamiltonians with a desired graph structure, in a variety of platforms and in a programmable way. Perhaps the closest work to our idealized setting is [PCK+21], which reports the engineering of various interaction graphs, including rings, chains, ladders, non-Archimedean geometries, in an array of atomic ensembles with 18 sites with 10^4 Rubidium atoms per site, interacting via spin-exchange. The collective magnetizations at each site can be treated as bosonic variables in the relevant regime, with a quadratic Hamiltonian. The paper also shows procedures to verify the interactions from two-point correlation functions of the magnetization, using a combination of symmetries of their model, an ansatz on the covariance matrix, a high inverse temperature approximation, and estimating the interaction matrix as the inverse of the covariance matrix – i.e. the classical method, expected to be valid at high temperature. Our methods give a way to estimate the interactions and the graph in these types of systems that are applicable at lower temperatures and without model-specific assumptions. We also mention [SWW+23], where quadratic bosonic Hamiltonian on programmable graphs have also been simulated via photonic synthetic lattices with up to 10^5 sites, where frequency modes are mapped to lattice sites, and [YKB+22], where lattice bosonic Hamiltonians, including honeycomb lattice structure, were engineered on 10 sites of a superconducting optomechanical system, and reconstructed the quadratic Hamiltonian with modeshape measurements. Finally, [KM23] is a theoretical proposal for lattice bosonic Hamiltonian simulation using phonons in trapped-ion crystals, interacting via the excitations of the trapped ion spins. In all the above, quadratic hopping Hamiltonians already explore interesting physics and testing their correct implementation is paramount to go forward to the simulation of interacting models.

The problem of Gaussian Hamiltonian learning can also be seen as a non-asymptotic instance of Gaussian multiparameter metrology, for which studies on the asymptotic achievable precision exist (via the Fisher information matrix, e.g. [NLSKA18]). Indeed, our algorithms also suggest many-body metrology applications [MMY+25]: without trying to achieve the optimal information-theoretic limits which are highly sensitive on precise values of the parameter themselves, assumed to be roughly known, our methods ensure that the estimate of any parameter encoded linearly in the Hamiltonian matrix would be robust with respect to uncertainties in the other parameters, since the full Hamiltonian can be estimated at once, with non-asymptotic guarantees. As an example, bosonic Gaussian systems are experimentally used as thermometric probes [PYK+15], and [CLAM22] studies how to optimize over feasible measurements (including those we use in out protocol) to infer the temperature of a Gaussian state departing from a *known* value.

As showcased by the various examples above, it is actually the case that quadratic Hamiltonians and their thermal and ground states are of *fundamental importance* for Bosonic systems and have been extensively studied from a variety of points of view and applications, even if we restrict to sparse graphs. Indeed, note that any lattice is sparse, and already the literature devoted to lattice quadratic Hamiltonians is sizeable (see e.g. [SCW06] and references therein). There is, however, one angle in which we agree with the referee: when it comes to learning or tomography, the literature on bosonic systems is much less developed. But there is now a large ongoing effort by the community

to address this gap. For instance, in the recently published [MMB+25] the authors touch upon basic questions such as lower bounds for estimating states (Gaussian or non-Gaussian) of m modes in trace distance. It is noteworthy that such questions were already answered for discrete systems over a decade ago [OW16]. Thus, we also see our work as a key part of this new effort and interest by the community to finally tackle such questions for Bosonic systems given their remarkable recent experimental progress showcased in the last paragraphs.

The referee also rightfully pointed out the important question of whether physically relevant systems do thermalize into Gaussian thermal states when evolving through a quadratic Hamiltonian and subject to reasonable mechanisms of dissipation. The textbook assumption that Gibbs states are thermal equilibrium states is often made also for bosonic systems, provided that the Gibbs state is well defined. This is the case for the quadratic Hamiltonians we consider, which are unitarily related to the sum of the harmonic oscillator Hamiltonians of the normal modes. In the theory of open quantum systems, the fact that Gibbs states are thermal equilibrium states can be justified through the microscopic derivation of the Lindblad master equation from an interaction with a large bath, using the Born-Markov and secular approximations [Dav74]. The result of this procedure is a time evolution that has the Gibbs state of the system Hamiltonian as fixed point and, if a certain ergodicity condition is satisfied, converges to the Gibbs state. The secular approximation requires that the matrix elements of the interaction Hamiltonians, written in a product basis, couple eigenstates of the system which have energies that are well-separated with respect to the inverse relaxation time. These assumptions are not inherently problematic for systems with quadratic Hamiltonians, even if they are infinite-dimensional. For example, if the system is coupled to the bath with terms of the form $\gamma a_i^\dagger \otimes B_i + h.c.$, then it only couples eigenstates with energies separated by the frequencies of the normal modes. While this approximation had tremendous success in quantum optics, it is not well justified in other settings, especially when the system of interest is large, see e.g. [SA25] where other approximation methods are proposed. Nevertheless, a recent paper showed a model that gives relaxation to Gibbs states for generic quadratic fermionic or bosonic models coupled to Gaussian baths satisfying detailed balance, where uniform interactions avoid the need of the secular approximation [SNM25]. We would also stress that for reconstructing the Hamiltonian, it would be enough that first and second moments thermalize to those of the Gibbs state, and that the covariance estimator stays sub-exponential. The former assumption is at the core of less strict definitions of thermalization [GEF19], while the latter is a very reasonable assumption for imperfect implementations of heterodyne measurements.

(2) Section 2.1 does not review any previous classical results in detail. It was only vaguely mentioned "From that, one is also usually interested in estimating the parameters of Θ, while having a scaling of the sample complexity that only scales logarithmically with the dimension of the random variables." What was the exact sample complexity achieved? What was the algorithm and was it efficient? Since the argument here is a classically useful technique—conditional independence—is not useful any more in the quantum case, will the local inversion technique introduced here for quantum also be useful for the classical case? Does it lead to the same result (derived using conditional independence)? If not, which parameter is not as good?

Reply. We have expanded and clarified the discussion on the classical case in the relevant section and we report the new information here for convenience. The problem of estimating Gaussian graphical models with sparsity is a central theme of high-dimensional statistics, and we refer to books such as [HTF09, HTW15, Wai19] for a more in-depth history of the problem. When the graph is known, a simple procedure using local inversions for each neighborhood suffices to find the coefficients very efficiently, as explained in Chapter 17 of [HTF09]. In the zero-mean case, $O(\frac{d}{\epsilon^2})$ are sufficient to learn a $d \times d$ covariance matrix in relative error, i.e. $\Sigma(1-\epsilon) \leq \hat{\Sigma} \leq \Sigma(1+\epsilon)$ [Wai19]. Applying local inversion to each neighborhood, symmetrizing, and via union bound, this means that $O(\frac{\Delta\|\Theta\|_\infty}{\epsilon^2}\log m)$ samples guarantee an entrywise error for the entries of $\Theta = \Sigma^{-1}$ of order $O(\epsilon)$, thus with no condition number dependence[1]. An essentially matching lower bound to this simple algo-

[1]It seems to us that a slight dependence on the diagonal elements of V is instead needed in the non-zero mean case,

rithm is given by Theorem 2 of [WWR10], which states that $\Omega(\frac{\Delta^2}{\epsilon^2}\log m)$ samples are necessary (here note that $\|\Theta\|_\infty \le \Delta \max_{s,t}|\Theta_{s,t}|$ by symmetry and sparsity). Let us thus stress that local inversions on neighborhoods at distance 1 would be already sufficient in the classical case, and this is because conditional independence ensures that the conditional distribution of a random variable X_i, when the other variables at distance one in the graph are known, contains the full information about the interactions of i with its first neighbors. Our local inversion method, instead, goes further in the graph to account for the lack of conditional independence.

In fact, most of the literature has directly dealt with the harder problem of estimating the precision matrix when the graph is not known, as well as estimating the graph itself. To do that, the main strategy is to build an estimator of the precision matrix as the solution of a regularized and/or constrained optimization problem, to enforce sparsity of the solution. A popular approach is the graphical LASSO, first studied in [FHT08, YL07], that consists in maximizing the l_1-regularized log-likelihood. The analysis of the sample complexity of the graphical LASSO [RWRY11] gives an upper bound $O(\frac{\Delta^2}{\alpha^2\epsilon^2}\log m)$ sample complexity, where α is a parameter encoding a certain incoherence condition of the precision matrix. Other approaches are based on estimating the precision matrix one row at a time, via LASSO [MB06], Danzig [Yua10], or l_1-constrained optimization (CLIME) [CLL11]. Simpler loss functions [ZZ14] and non-convex methods have also been considered [WRG16]. These algorithms that are all efficient to implement, scaling polynomially with the number of variables. The sample complexity is $O(\log m)$, but only if appropriate assumptions, that can be broadly linked to the condition number being bounded, hold [MVL20]. For the problem of graph selection, the paper [MVL20] showed a sample complexity $O(\frac{\Delta \log m}{\kappa^2})$, with κ being a lower bound on the relative strenghts of the interactions, and no condition number dependence, and matching the κ and m dependence in the lower bounds in [WWR10]. The tradeoff is a worse scaling in computational complexity, still polynomial in m but with a degree depending on the degree of the graph, since for each node one needs to test all possible neighborhoods (but see improvements for certain models in [KKMM20]). Our graph learning algorithm is inspired by the approach in [MVL20] (especially their DICE algorithm). However, we were not able to remove the condition number dependence, and leave it to future work.

*(3) The authors should clarify the differences between spin Hamiltonian and Gaussian bosonic Hamiltonian. Here, within the Gaussian framework, every operator can be efficiently represented using a 2m*2m matrix that scale only linearly in m, but for discrete variables, the size of the Gibbs state scales exponentially with respect to the number of qubits. Therefore, it seems less challenging to achieve the logarithmic sample complexity and the polynomial computation complexity in the Gaussian framework than in spin systems (as reviewed in the introduction part). Also, it seems that a fair comparison can only be made between Gaussian state learning and stabilizer state learning, instead of general local Hamiltonian learning.*

Reply. We thank you for prompting us to clarify the relation between spin and bosonic Hamiltonian learning. First, let us clarify that for discrete variables, knowing the expectation values of the observables appearing in the Hamiltonian is enough to reconstruct their coefficients, as a manifestation of the maximum entropy principle [Jay57, AAKS21]. The interesting setting, from a learning point of view, is when each local spin is interacting with at most a finite number (not depending on the total size of the system) of spins. In this case, the number of terms allowed in the Hamiltonian is still scaling linearly with the size of the system. Otherwise, one cannot expect Hamiltonian learning to be any more useful than general-purpose quantum tomography. In the Gaussian case with interactions with bounded degree, the coefficients to learn are also linear in the number of modes. Therefore, the number of parameters are comparable when comparing spin systems and CV and, at first glance, one may in fact think that it is the infinite dimensional case that is harder to analyze, just because the states live in a larger space. Of course, the reason this is not true is that the Hamiltonian of Gaussian states has an analytic expression as a function of the covariance matrix, collecting expectation values of the observables in the Hamiltonian. A relation of this type is not available for

which is not always treated in the literature. To learn the precision matrix alone, one can simply subtract copies of samples to get a zero-mean gaussian with twice the covariance matrix.

spin systems. A similar situation occurs in the classical case, for classical spin systems and Gaussian graphical models.

An important difference between the quantum and the classical case, is that for both classical spin systems and Gaussian models, and in fact any exponential family, the interactions between one vertex of the graph and its neighbors can be learned in principle by sampling from the neighborhood of the vertex. However, it should be noted that even the classical literature on learning exponential families beyond Gaussians (the classical version of learning Gibbs states beyond quadratic Hamiltonians) does not, to the best of our knowledge, have rigorous guarantees for learning such models efficiently [RMVL22]. Even if the case beyond quadratic Hamiltonian is certainly very important to address learning of states that cannot be simulated efficiently classically, given that even the corresponding classical problem is open, we restrict our attention in this work to quadratic Hamiltonians. We want to stress that our work is the first to successfully address, in generality, the natural quantum generalization of the main problem that is extensively studied classically in the continuous variable case. Since the status of quantum Hamiltonian learning is rapidly evolving and more efficient algorithms for discrete systems appeared [CAN25] after our initial arXiv post, we have anyway removed sentences attempting a direct comparison between the status of discrete and bosonic Hamiltonian learning, in the interest of the current and future readers. We are also not aware of a sensible qudit Hamiltonian learning problem that could be fairly compared to the Gaussian case: the main point we use is the analytic relation between the covariance matrix and the Hamiltonian, and this does not seem to be available for most studied families of discrete variable Hamiltonians. While there is an analogy between stabilizer operations and Gaussian unitaries, we are not aware of a correspondence between mixed Gaussian states, which are thermal states of quadratic Hamiltonians, and some meaningful notion of thermal stabilizer states.

(4) There are numerous parameters/notations introduced in this manuscript. It is difficult for readers to immediately remember the meanings of each parameter when they appear and it takes a lot of time to find out each definition given that there are so many parameters. A table that shows the notations, the definitions, and maybe where they appear can hugely improve the clarity. Furthermore, a comparison between the dependence of sample complexity on these parameters to previous results is lacking.

Reply. Thank you for the suggestion. We have now introduced Table 1, where we collect the notation and the relations between the parameters. For trace distance learning, we have added a comment after Theorem 3.1 on the comparison with previous and concurrent results.

(5) There is no discussion on lower bounds. For example, $1/epsilon^2$ scaling is usually optimal. Is this also the case here? Does the benefit of $1/epsilon^2$ introduce some side effects, like making dependence on other parameters significantly worse? Also, are the logarithmic sample complexity and the polynomial computation complexity optimal?

Reply. Thank you for this important question. We are not aware of concrete lower bounds in the quantum case. The paper [WWR10] has lower bounds for the classical case, that read $\Omega(\frac{\Delta^2}{\epsilon^2}\log m)$. Since in the high temperature, small $\|H\|_\infty$ limit the state behaves like classical, we expect these bounds to hold also for the quantum case. A detailed study and dedicated bounds for the quantum case would be very important, but we believe that this is beyond the scope of the present paper. On the other hand, the $\Omega\left(\frac{1}{\epsilon^2}\right)$ lower bound would result from the fact that the trace distance between two states with ϵ-close Hamiltonians would be $O(\epsilon)$ (via e.g. our continuity bounds), and this implies a standard $\Omega\left(\frac{1}{\epsilon^2}\right)$ lower bounds for distinguishing between them. The $\Omega(\log m)$ lower bound could be obtained from a standard information theoretic argument: one could encode an m-bit string $\vec{i} \in \{0,1\}^m$ into a product of thermal states of single mode harmonic oscillators of the form $\rho_{\vec{i}} = \rho_{i_1} \otimes \rho_{i_2} \otimes ... \otimes \rho_{i_m}$, by choosing the temperature of each mode among two alternatives far enough that the learning algorithm would identify the correct bit-string with high-probability. Since the mutual information of classical-quantum state $\frac{1}{n}\sum_{\vec{i}\in\{0,1\}^m} \left|\vec{i}\middle\rangle\!\middle\langle\vec{i}\right| \otimes \rho_{\vec{i}}^{\otimes n}$ would be at most linear in n, it means that $n = \Omega(\log m)$ via Fano inequality. These arguments are standard and fall short of

capturing the right complexity, therefore we would prefer not including them in the paper. For the computational complexity, listing the number of variables to learn already takes linear time in m, therefore polynomial time is unavoidable.

Other comments: (1) The result section lacks a discussion of computational complexity—although it was mentioned multiple times in introduction. Even in the Methods section, the details of computational efficiency are buried in the proofs of sample complexity. Clear algorithms should be given.

Reply. Thank you for the suggestion. We have added discussions on the computational complexity in the Results section, and added a figure with the Hamiltonian learning algorithm. The figure with the graph learning algorithm was already present, but we simplified the pseudocode. Regarding the local inversion subroutine, it is specified in the relevant Definition (see also next point).

(2) The presentation of Fig 1 is quite confusing. There are four colors. What do they represent respectively? For example, what are the differences between the yellow and the red squares in the second step? (A figure has to be self-explanatory, but here readers cannot understand its meaning unless after reading Section 4.) Maybe a drawing of an interaction graph can improve the clarity.

Reply. We welcome the suggestion of adding a figure with the interaction graph and the corresponding structure of the Hamiltonian matrix. This should clarify that the yellow squares represent the submatrices relating to the neighborhood of size 1 of the vertex 1. We also slightly modified Fig.2 and, in its caption, we give more details on the reconstruction through local inverses.

(3) The definition of Omega before Eq.(1) is confusing. First, the notation diag() was not explained. Second, It was said "where all blocks are $m \times m$ matrices", shouldn't it be 2×2?

Reply.
Thank you, we have corrected the definition using the direct sum notation.

(4) In Definition 4.8, the first sentence right below the equation should be "The Gaussian graph learning problem..."

Reply.
Thank you, corrected.

Reply to Reviewer 3

Reviewer 3 (Remarks to the Author):

This paper addresses three core learning problems for Gaussian states: inferring Hamiltonian parameters, learning the interaction graph structure, and learning the full quantum state in trace distance, all using experimentally friendly heterodyne measurements. The central achievement is the "local inversion technique," which allows for estimating the Hamiltonian from local data, leading to a breakthrough sample complexity that scales only logarithmically with the system size for Hamiltonian and graph learning. Furthermore, the work provides the first trace distance learning results for these systems with an efficient quadratic scaling in precision, under some state restrictions. The authors also develop new, independently valuable continuity bounds relating Hamiltonians and covariance matrices, underpinning their learning protocols. While the paper introduces promising learning protocols with favorable scaling in system size, the practical efficiency of these methods can be significantly impacted by a strong dependence on other system parameters, potentially leading to very high sample or computational costs for general cases or when high precision is required. The conditions under which the algorithms are truly "efficient" are quite specific and may not hold broadly without further refinements. As it stands, this contribution appears to be a technical, theoretical advance of interest primarily to the Hamiltonian-learning theory community. In the absence of additional evidence or empirical benchmarks demonstrating its wider applicability, I cannot recommend acceptance at this time.

Reply.
We thank the reviewer for recognizing the interest of our contributions. We want to stress that the current literature on quantum Hamiltonian learning for discrete systems has sample complexity bounds that also depend badly on the parameters different from m, i.e. superexponentially in the inverse temperature for state-of-the-art results [CAN25]. The focus on the scaling in m comes from the fact that it is the right parameter to encode the complexity of the Hamiltonian family. Given the fundamental importance of quadratic bosonic Hamiltonians in physics, and the current interest in implementing arbitrary lattices on graphs (see answer to Reviewer A), we believe that the proof of principle that the $O(\log m)$ scaling at any temperature is even possible, is a key contribution, from both the technical and conceptual points of view, and that it will be of interest for the wider physics community. Nevertheless, we have followed the suggestion of the reviewer and have at least implemented the Hamiltonian learning algorithm for a Hamiltonian on a 1D lattice (line). Very interestingly, the algorithm performs better than the plug-in inversion, both in the well-conditioned case and in the the badly-conditioned case, suggesting that our analysis could actually be improved. In addition, the protocol performed well already with 10^4 shots to estimate the 1D Hamiltonian in our numerics for systems with more than one thousand modes. Thus, we believe that these numerics, together with the feasible measurements, clearly indicate that the protocol is practical. In addition, although we agree that the analysis could potentially be refined at some stages, we stress once again that this is the first result in this direction. It is only natural in such situations that further refinements happen after a first breakthrough, which is the case here, as acknowledged by the referee.

Here are some of our questions: 1. The sample complexities for Hamiltonian and graph learning also exhibit, in some cases, a very steep dependence on other physical parameters like the bounds on symplectic eigenvalues ($d_{\min}$, $d_{\max}$), squeezing ($\|S\|_\infty$), and graph degree (Δ). For instance, Corollary 6.1 shows a dependency involving poly$\{\Delta^{\Delta\beta_{\max}}(\dots)^{\ln\Delta}\}$, and the number of samples N in Theorem 7.1 for graph learning scales with ζ^{-2}, where ζ itself has a complex and potentially very small value depending exponentially on l (which in turn depends on Δ, $\beta_{\max}$) and other parameters. The authors acknowledge the dependence on $d_{\min}$ (related to a condition number) and discuss its classical precedents. However, the practical utility of the "efficient" label hinges on these other parameters not leading to prohibitively large prefactors. If Δ or $d_{\max}$ are large, the actual number of samples required could become immense, limiting the applicability in some physical regimes despite the favorable $\ln(m)$ scaling. The paper assumes "constant-size bounds on the relevant parameters" for its main claims, but the impact of these "constants" being large warrants careful consideration for practical implementation.

Reply. The concerns of the referee are well-motivated, but, as we argued in the previous reply, we believe that, at this stage, showing that logarithmic sample complexity in system size is a key message that we anticipate will motivate further study to improve the dependence on the other parameters. Nevertheless, our numerics already show that in simple examples our method is already competitive with a naive plug-in method.

2. The Learn Graph Gaussian algorithm (Algorithm 1) has a stated computational complexity of $\mathcal{O}\left(m\binom{m}{\xi(l)}\binom{m-\xi(l)}{\xi(l)}\xi(l)^3\right)$. The parameter l (local neighborhood extent) is chosen based on Δ, $d_{\max}$, and the target precision κ, potentially scaling as $l = \mathcal{O}(\Delta^2 d_{\max} + \ln(1/\kappa))$. The neighborhood size $\xi(l)$ can grow as Δ^l. The authors state this is polynomial "assuming these parameters [Δ, d_{max}] to be of constant order". However, if Δ is not a very small constant, or if $d_{\max}$ is large, l can become significant. If $\xi(l)$ is not a small constant (independent of m), the binomial term $\binom{m}{\xi(l)}$ can render the algorithm super-polynomial in m. For the algorithm to be truly polynomial-time in m for general graphs, $\xi(l)$ must be $\mathcal{O}(\log m/\log\log m)$ at best, or more strictly, a constant. The conditions for $\xi(l)$ to be this small are quite restrictive (requiring very small Δ and l). This is a notable limitation for the practical efficiency of graph learning on more general or densely connected local neighborhoods.

Reply. Thanks to our refined analysis (Lemma 4.11), the dependence of l on the various parame-

ters has been improved: in both Hamitonian learning and graph learning it satisfies $l \leq \frac{2\log\frac{C_1}{\epsilon}+C_2 e}{1+\log\left(\frac{\log\frac{C_1}{\epsilon}}{C_2 e}+1\right)}$, where $C_2 = O(\text{poly}(\Delta d_{\max}))$ and $C_1 = O(\text{poly}(\|S\|_\infty e^{d_{\max}} \frac{1-e^{2\beta_{\max}}}{1-e^{2\beta_{\min}}} \frac{1}{(\kappa,\epsilon)}]))$. This should alleviate in part the concerns of the referee, e.g. the dependence on $\beta_{\max}$ becomes $l \sim \frac{d_{\max}}{\log d_{\max}}$, and the one on κ is $l \sim \frac{\log\kappa^{-1}}{\log\log\kappa^{-1}}$. Admittedly, the graph learning we propose here does not have the ambition of being the last word on the subject, as for the DICE algorithm in [MVL20] that inspires it. Rather, we believe that is important to stress that this is the first example of a graph learning algorithm for a non-trivial problem, with a favorable dependence on m, and that it will motivate further improvements, again in the same way that DICE delivered essentially optimal results for the sample complexity but left open improvements in computational complexity. We anticipate that global reconstruction methods with sparsity constraints, such as the older classical methods (that cannot avoid condition number dependence, but have controlled computational complexity, see the new literature review above), could give much more efficient sample complexities. However, this could only be addressed in a follow-up, as it is highly non-trivial how to formulate the optimization problem in the quantum case.

3. The sample complexity for Hamiltonian learning scales as $\epsilon^{-(2+4\ln\Delta)}$ and for graph learning as $\kappa^{-(2+6\ln\Delta)}$ for general graphs. The authors acknowledge this is "not the expected ϵ^{-2} (or κ^{-2})" and identify it as a potential limitation of their Taylor expansion-based approach for the general case. While acknowledged, this is a significant point because for graphs with even moderate degree Δ, the exponent on the precision can become substantially larger than 2 (e.g., if $\ln\Delta \approx 1$, the exponent is ≈ 8). This would make achieving high precision very costly in terms of samples, more so than typical learning algorithms. This is an important trade-off for achieving the $\ln m$ scaling.

Reply. Our refined analysis (Lemma 4.11) has partially solved this issue: with l being $l \sim \frac{\log\epsilon^{-1}}{\log\log\epsilon^{-1}}$, $\lim_{\epsilon\to 0}\Delta^l\epsilon^\gamma = 0$, so the contribution of the term Δ^{6l} to the sample complexity is, at all the other parameter fixed, $o(\frac{1}{\epsilon^\gamma})$ for every $\gamma > 0$. This implies a sample complexity upper bound $N = O(\frac{1}{\epsilon^{2+\gamma}})$ for every $\gamma > 0$. In practice, this behaviour is only visible at incredibly small ϵ, but already for $6\log\Delta \approx 6$, and $\epsilon \approx 10^{-6}$, $\frac{6\log\Delta}{\log\log\frac{1}{\epsilon}} \approx 2.1$. At the moment, we are not able to guess if this supolynomial correction is unavoidable, but we would also like to stress that it is not trivial that a dependence on $O(\text{poly}(\frac{1}{\epsilon})\log m)$ is even possible for general graphs (see e.g. [CAN25], which needs exponential dependence to get the $O(\log m)$ scaling for general graphs). The reviewer may have noticed, but we also remark that we get $\tilde{O}(\frac{1}{\epsilon^2})$ (in the usual sense of neglecting polylog terms) for polynomially growing neighborhoods.

4. To underscore the practical relevance and broader appeal of this work to the quantum information and physics communities, we recommend that the authors include performance benchmarks—such as explicit sample-complexity improvements—for experimentally relevant bosonic Gaussian states. Moreover, it remains unclear whether the proposed protocol maintains its advantages in the presence of realistic heterodyne-measurement imperfections and finite-sample statistical fluctuations; an analysis or empirical evaluation of its robustness under these conditions would greatly strengthen the manuscript.

Reply. We thank the referee for the suggestions. We included a numerical example of 1D states, for the case of well-conditioned and ill-conditioned Hamiltonians (see Fig. 4 in the revised main text). Importantly, we see that even with a sample size of 10^4 (which is feasible in most platforms), we are able to obtain through the local inverse method a good recovery that is essentially independent of the number of modes and that is markedly better than the global method. Furthermore, we observe a performance that is markedly better than one would anticipate solely by our bounds.

In addition to this finite sample analysis, it is worth noting that all of our bounds take into account the probability of error δ with finite samples and have a $O(\log\frac{1}{\delta})$ scaling in this parameter. Thus, our current analytical bounds already ensure that even with moderate sample sizes, the probability of substantial errors due to statistical fluctuations is negligible.

Regarding the robustness of our methods, two aspects are worth mentioning. First, in realistic

continuous-variable experiments, the dominant imperfections in heterodyne detection are (i) finite detection efficiency and (ii) electronic or dark noise in the readout stage (see e.g. [LR09, Sec. IV]). Both effects are standardly modelled by Gaussian channels: each mode is passed through an attenuator (a beam splitter mixing with vacuum) with transmissivity η, followed by additive classical Gaussian noise with variance v_{el}. At the level of the heterodyne outcomes this amounts to replacing the ideal covariance

$$\Sigma = V + \frac{\mathbb{I}}{2}$$

by

$$\Sigma_{\mathrm{meas}} = \eta\Sigma + \frac{1-\eta}{2}\mathbb{I} + v_{\mathrm{el}}\mathbb{I} = \eta V + \frac{\mathbb{I}}{2} + v'\mathbb{I}.$$

for $v' = \frac{v_{el}-\eta}{2}$. Thus, the measured data are still samples from a multivariate normal distribution. In particular, our basic assumption that heterodyne measurements produce Gaussian samples remains valid in the presence of these experimental imperfections.

This channel only rescales inter-mode covariances and adds a diagonal contribution; it does not create new off-diagonal entries in the covariance matrix. Thus, if we have good estimates on the parameters η and v_{el}, we can define rescaled estimators that allow us to obtain V from the noisy estimates with an additional sample complexity of order η^{-2}. But, in a nutshell, if we have an estimate of the strength of these noise dominant noise sources and they are independent of system size, we can define rescaled estimators that are independent of system size and take this noise into account.

More generally, any sufficiently small (this time also potentially uncharacterized or unknown) Gaussian noise (loss, thermal noise, or general Gaussian channels that are close to the identity) acts linearly on V and hence only perturbs the covariance matrix without changing the Gaussian nature of the state. Thus, our protocol will also be robust against non-dominant noise sources.

To see this, note that our error analysis is characterized in terms of the entry-wise error on the estimate of the covariance matrices. If the noise generates a covariance matrix V_{eff} that contributes with an error which is small compared to the entry-wise precision that we need from our guarantees, our algorithm will be robust and it will not be able to tell the difference between the noiseless and noisy case. In particular, it may be that the noise induces a $O(\mathrm{poly}(m))$ at the level of $\|H(V) - H(V_{\mathrm{eff}})\|_\infty$, but our estimate of $H(V)$ obtained from V_{eff} will still have error $O(1)$ because we have processed V_{eff} locally.

Note also that our concentration bounds only used the sub-Gaussian and sub-Gamma properties of the estimators. If the implementation of the heterodyne measurement is not Gaussian (in fact, the standard implementation requires an infinite-energy local oscillator) but the estimators have expectation values sufficiently close to t and V, and above sub-Gaussian and sub-Gamma properties are still satisfied, the conclusions of the analysis do not change. We refer to Remark 4.1 of the main paper for a discussion of these aspects.

Reply to Reviewer 5

Reviewer 5 (Remarks to the Author):

Overall this is an interesting paper addressing an interesting problem in quantum learning. The results are technically sound and the main ideas of the paper are well explained. The relative importance of this result seem to be overstated in some parts of the paper and relation to recent literature is not very well explained. We have the following questions/comments about the paper that I hope the authors can answer:

Reply. Thank you for recognizing the value of the paper, and for the suggestions. We have improved the comparison with recent literature.

1. In the abstract the statement: "Taken together, our results put the status of the quantum Hamiltonian learning problem for continuous variable systems in a more advanced state when compared to spins, where state-of-the-art results are either unavailable or quantitatively inferior to ours." should be removed. The

quadratic continuous variable case considered here is restricted compared to the more general local interactions considered in Baksi et.al. for the discrete case. To make this statement fair, the learning task here must be extended beyond quadratic Hamiltonians.

Reply. We thank you for prompting us to clarify the relation between spin and bosonic Hamiltonian learning. Reviewer 1 had a similar concern and we have assessed it the reply to their "major comment" (3). We have removed sentences attempting a direct comparison between the discrete and Gaussian Hamiltonian learning. We would like to repeat here that in the classical case, results on efficiently learning exponential families beyond Gaussian are much less explicit [RMVL22], making Gaussian models the main extensively studied family of graphical models (see the literature review in the reply to "major comment" (2) of Reviewer 1). Our comparison was aimed at expressing that, by recovering the $O(\log m)$ scaling, we make the gap between the classical and quantum results smaller than for general spin systems. Since the $O(\log m)$ scaling has been subsequently anyway proven in spin systems [CAN25], we have decided to avoid to make such statements, for the benefit of the reader.

2. The authors briefly mention the difference between this work and other recent results [MMB+24, Hol24] [BMM+24]. This has to be made more explicit. For instance, what extra assumptions are made in this work compared to [MMB+24]? A table showing the results and assumptions of this paper against the same from other works would be ideal for this.

Reply. Thank you for the suggestion. We have added a discussion after Theorem 3.1 to compare with previous results and report it here for convenience: Note that this is the first result for this problem with an ϵ^{-2} scaling [MMB$^+$25], together with [BMM$^+$24]. In fact, [MMB$^+$25] used a different parametrization than ours and expressed the sample complexity upper bounds in terms of an upper bound on the expected energy of the state. Theorem 4 in [MMB$^+$25] shows an upper bound $\mathcal{O}(\frac{m^7E^3}{\epsilon^4})$, while Theorem 5 in the concurrent paper [BMM$^+$24] improved this to $\mathcal{O}\left(\frac{E^4}{\epsilon^2}\left(m+\log\frac{2}{\delta}\right)\right)$. We can express our bound in terms of the expectation value of a bound on the energy of the state to learn, and of a bound $\beta_{\max} \geq d_{\max}$ as $N = \mathcal{O}\left(\frac{m^3E^4(e^{\beta_{\max}}-1)^2}{\epsilon^2}\log\left(\frac{m}{\delta}\right)\right)$. We see that we have to assume not only bounded energy but also bounded value of $\beta_{\max}$: highest values mean that the state is closer to being non-full rank, and in that case the Hamiltonian is not well-defined and our approach cannot work. This means in particular our approach does not cover pure state learning. We also mention that alternative trace distance bounds with $O(\sqrt{\epsilon})$ scaling were obtained by [Hol24b], [Hol24a], who argued that while suboptimal for the trace distance, this behaviour is tight for the Bures distance [Hol24a]. We note that using the energy as a parameter for our graphical models algorithm would not be very natural, as it typically scales with system size, even for families of models where our parameter choice does not depend on system size.

3.What are the computational complexities of the learning tasks solved in Them 3.1 - 3.3 in terms of the problem parameters?

Reply. The computational complexity are given by the time it takes to sample (the sample complexity) plus the classical post-processing. We have given more details on the computational complexity after the main theorems, and added a figure with the Hamiltonian learning algorithm, with known graph. This should clarify that the main cost in the classical post-processing involves only operations on matrices of size at most $2m \times 2m$, that can be realized e.g. via Jordan decomposition in polynomial time in m, with exponent with no dependence on the other parameters. The number of such operations is just one in the plug-in algorithm, $m+1$ in the known graph case (the number of local inversions + computing $\hat{H}$ from $\mathrm{LI}_{\mathcal{N}}(2V - i\Omega)$). For Theorem 3.3, the situation is considerably worse, as we have to iterate over all possible neighborhoods of a size that depend on the parameters of the problem. When the other parameters are bounded by constants, this still gives a $O(\mathrm{poly}(m))$ dependence, but with exponent depending on the parameters. This is explained in Remark 4.9. In fact, the stability of the matrix function operation would also depend on the singular values and eigenvalues of the matrices involved, but we believe that these details would go beyond the scope of

the paper and would require careful search for the best numerical analysis algorithms. Nevertheless, it is perhaps useful to say that the plug-in inversion cannot be performed if the estimate $(2\hat{V} - i\Omega)$ is not invertible or even just far from psd, and this is highly likely in the regime where the number of observations is less than m. In the same regime, the procedure using local inversion would be instead possible.

4. $\hat{H}$ seems to be arbitrarily introduced before Eq 20. Readers will find it easier if the computation of $\hat{H}$ is briefly explained at this point. Including the local inversion + patching algorithm as a box somewhere in the beginning of the paper will also improve readability of the paper.

Reply. Strictly speaking, the regularization of $\hat{H}$ is only needed for the trace distance bound, and we have added a pointer there. It can be done essentially for free also for the Hamiltonian learning problem, but it is not necessary to obtain the operator norm error guarantee, and thus a guarantee on the entry-wise error. We have anyway added a figure with Algorithm 1, pointing at the relevant definition of the local inversion.

5. Is the epsilon in (18) the same as (20)?

Reply. Those are generic error parameters ϵ, which are not related at that point. There, we do not need to specify them in more detail for our purposes. For the trace distance bound to work, they can be taken equal, except that the ϵ-independent upper bound on $\|V - \hat{V}\|_\infty$ must hold anyway.

6. In Theorem 4.1, what is the significance of the assumed upper bound on the operator norm between V and $\hat{V}$? Is this assumption restrictive? Does it impose additional constraints on the precision ϵ in the same theorem? Our main concern here is the following scenario: if this upper bound is too close to zero, this forces the precision to be artificially small, effectively increasing the sample complexity of some problems. This concern can be alleviated if it can be shown that the upper bound is system size independent.

Reply. Thanks for pointing this out. In the proof, we require $|V_{ij} - \hat{V}_{ij}| \le \epsilon' = O(\epsilon m^{-1.5} \|S\|_\infty^2 (e^{2\beta_{\max}} - 1))$. Since $2\|V - \hat{V}\|_\infty \le 4m\epsilon'$, the assumed upper bound $2\|V - \hat{V}\|_\infty \le 4m\epsilon' \le \|S\|_\infty^{-2} (e^{2\beta_{\max}} - 1)^{-1})$ is automatically satisfied and it does not increase the sample complexity. We have now given the bound of $\|S\|_\infty$ in terms of the energy, while $e^{2\beta_{\max}} - 1$ needs to be assumed bounded independently, as we explained in the previous point on the comparison with other trace distance bounds. Both $\|S\|_\infty$ and $\beta_{\max}$ can be system independent in a variety of models (see next reply), but, specifically for trace distance estimation, the dependence of the number of samples on the system size is anyway unavoidable.

7. How does the operator norm of S scale for physically relevant systems (local interactions, bounded interaction strength)?

Reply. Hamiltonians that commute with the total number of particles have the form $\sum_{i,j} a_i^\dagger K_{ij} a_j$, where we expressed position and momentum operators in terms of creation and annihilation operators. Here K must be a complex psd matrix and it is diagonalized by a unitary matrix. As a symplectic transformation of the phase space, this is an orthogonal symplectic transformation and it has $\|S\|_\infty = 1$. For the non-number-preserving case, we have $\|S\|_\infty \le \frac{\|H\|_\infty}{d_{\min}} \le \frac{\|H\|_\infty}{\|H^{-1}\|_\infty}$. First of all, we see that a temperature rescaling does not affect the upper bound, so that we can assume entries of H less than C in absolute value. In that case, the norm of $\|H\|_\infty$ is bounded by $2C\Delta$. However the minimum symplectic eigenvalue can be arbitrarily small, and this would reflect in a high norm of the covariance matrix and a small energy gap of the Hamiltonian operator associated to H. However, for any family of Hamiltonians with bounded entries and increasing m, just adding a constant multiple of the identity would give a problem where the minimum eigenvalue would be bounded from below. For example, a very natural family of Hamiltonian with bounded condition number is the grid of coupled harmonic oscillators on any graph:

$$H = \sum_{i=1}^{2m} \frac{P_i^2}{2} + \frac{\omega^2 X_i^2}{2} + \sum_{ij} J_{ij}(X_i - X_j)^2$$

Clearly, the part of the Hamiltonian matrix that one obtain from the couplings is positive-semidefinite, and the one obtained from the free part is positive definite, with smaller eigenvalue given by $\min\{\frac{1}{2}, \frac{\omega^2}{2}\}$. In terms of the performances of our algorithm, numerical evidence suggests that the dependence on the condition number should be much milder, but we are not yet able to prove it.

8. In the proof of lemma 4.6. What do the authors mean when they write $EE^\dagger \leq (...)$ *Is it supposed to mean that RHS -LHS is a PSD matrix. If so, please clarify this in the text. May be different notation (curly inequalities) would be helpful for denoting matrix inequalities.*

Reply.
Thank you, we clarified this in the notation section.

9. In section 4.3, can the sparse approximation be improved by using another polynomial basis. For instance, using a Chebyshev expansion instead of a Taylor expansion in Lemma 4.6?

Reply.
Thank you for the suggestion. While it is true that one can get improved approximations of the exponential via Chebyshev polynomials [AA22], at the moment, we cannot see any qualitative improvements from using them in the analysis.

Bibliography

[AA22] Amol Aggarwal and Josh Alman. Optimal-degree polynomial approximations for exponentials and gaussian kernel density estimation. In *Proceedings of the 37th Computational Complexity Conference*, CCC '22, Dagstuhl, DEU, 2022. Schloss Dagstuhl–Leibniz-Zentrum fuer Informatik.

[AAKS21] Anurag Anshu, Srinivasan Arunachalam, Tomotaka Kuwahara, and Mehdi Soleimanifar. Sample-efficient learning of interacting quantum systems. *Nature Physics*, 17(8):931–935, August 2021.

[BMM+24] Lennart Bittel, Francesco Anna Mele, Antonio Anna Mele, Salvatore Tirone, and Ludovico Lami. Optimal estimates of trace distance between bosonic gaussian states and applications to learning. *arXiv preprint arXiv:2411.02368*, 2024.

[CAN25] Chi-Fang Chen, Anurag Anshu, and Quynh T Nguyen. Learning quantum gibbs states locally and efficiently, 2025. arXiv preprint arXiv:2504.02706.

[CHK+20] Iacopo Carusotto, Andrew A. Houck, Alicia J. Kollár, Pedram Roushan, David I. Schuster, and Jonathan Simon. Photonic materials in circuit quantum electrodynamics. *Nature Physics*, 16(3):268–279, March 2020. Publisher: Nature Publishing Group.

[CLAM22] Marina F.B. Cenni, Ludovico Lami, Antonio Acín, and Mohammad Mehboudi. Thermometry of gaussian quantum systems using gaussian measurements. *Quantum*, 6:743, June 2022.

[CLL11] Tony Cai, Weidong Liu, and Xi Luo. A Constrained ℓ_1 Minimization Approach to Sparse Precision Matrix Estimation. *Journal of the American Statistical Association*, 106(494):594–607, June 2011. Publisher: ASA Website _eprint: https://doi.org/10.1198/jasa.2011.tm10155.

[Dav74] E. B. Davies. Markovian master equations. *Communications in Mathematical Physics*, 39(2):91–110, June 1974.

[FHT08] Jerome Friedman, Trevor Hastie, and Robert Tibshirani. Sparse inverse covariance estimation with the graphical lasso. *Biostatistics*, 9(3):432–441, 2008.

[GEF19] Marek Gluza, Jens Eisert, and Terry Farrelly. Equilibration towards generalized Gibbs ensembles in non-interacting theories. *SciPost Physics*, 7(3):038, September 2019.

[Hol24a] A. S. Holevo. On estimates of the bures distance between bosonic gaussian states, 2024. arXiv preprint arXiv:2412.04875.

[Hol24b] A. S. Holevo. On estimates of trace-norm distance between quantum gaussian states, 2024. arXiv preprint arXiv:2408.11400.

[HTF09] Trevor Hastie, Robert Tibshirani, and Jerome Friedman. *The Elements of Statistical Learning*. Springer Series in Statistics. Springer, New York, NY, 2009.

[HTW15] Trevor Hastie, Robert Tibshirani, and Martin Wainwright. *Statistical Learning with Sparsity: The Lasso and Generalizations*. Chapman and Hall/CRC, New York, May 2015.

[Jay57] E. T. Jaynes. Information theory and statistical mechanics. *Phys. Rev.*, 106:620–630, May 1957.

[JBC+98] D. Jaksch, C. Bruder, J. I. Cirac, C. W. Gardiner, and P. Zoller. Cold Bosonic Atoms in Optical Lattices. *Physical Review Letters*, 81(15):3108–3111, October 1998. Publisher: American Physical Society.

[KKMM20] Jonathan Kelner, Frederic Koehler, Raghu Meka, and Ankur Moitra. Learning some popular gaussian graphical models without condition number bounds. *Advances in Neural Information Processing Systems*, 33:10986–10998, 2020.

[KM23] Or Katz and Christopher Monroe. Programmable Quantum Simulations of Bosonic Systems with Trapped Ions. *Physical Review Letters*, 131(3):033604, July 2023. Publisher: American Physical Society.

[LR09] A. I. Lvovsky and M. G. Raymer. Continuous-variable optical quantum-state tomography. *Reviews of Modern Physics*, 81(1):299–332, March 2009.

[LSA+12] Maciej Lewenstein, Anna Sanpera, Verònica Ahufinger, Maciej Lewenstein, Anna Sanpera, and Verònica Ahufinger. *Ultracold Atoms in Optical Lattices: Simulating quantum many-body systems*. Oxford University Press, Oxford, New York, March 2012.

[MB06] Nicolai Meinshausen and Peter Bühlmann. High-dimensional graphs and variable selection with the Lasso. *The Annals of Statistics*, 34(3):1436–1462, June 2006. Publisher: Institute of Mathematical Statistics.

[MMB+25] Francesco A. Mele, Antonio A. Mele, Lennart Bittel, Jens Eisert, Vittorio Giovannetti, Ludovico Lami, Lorenzo Leone, and Salvatore F. E. Oliviero. Learning quantum states of continuous-variable systems. *Nature Physics*, November 2025.

[MMY+25] Victor Montenegro, Chiranjib Mukhopadhyay, Rozhin Yousefjani, Saubhik Sarkar, Utkarsh Mishra, Matteo G. A. Paris, and Abolfazl Bayat. Review: Quantum metrology and sensing with many-body systems. *Physics Reports*, 1134:1–62, July 2025.

[MVL20] Sidhant Misra, Marc Vuffray, and Andrey Y. Lokhov. Information theoretic optimal learning of gaussian graphical models. In Jacob Abernethy and Shivani Agarwal, editors, *Proceedings of Thirty Third Conference on Learning Theory*, volume 125 of *Proceedings of Machine Learning Research*, pages 2888–2909. PMLR, 09–12 Jul 2020.

[NLSKA18] Rosanna Nichols, Pietro Liuzzo-Scorpo, Paul A. Knott, and Gerardo Adesso. Multiparameter Gaussian Quantum Metrology. *Physical Review A*, 98(1):012114, July 2018. arXiv:1711.09132 [quant-ph].

[OW16] Ryan O'Donnell and John Wright. Efficient quantum tomography. In Daniel Wichs and Yishay Mansour, editors, *Proceedings of the 48th Annual ACM SIGACT Symposium on Theory of Computing, STOC 2016, Cambridge, MA, USA, June 18-21, 2016*, pages 899–912. ACM, 2016.

[PBSM15] V. Peano, C. Brendel, M. Schmidt, and F. Marquardt. Topological Phases of Sound and Light. *Physical Review X*, 5(3):031011, July 2015. arXiv:1409.5375 [cond-mat].

[PCK+21] Avikar Periwal, Eric S. Cooper, Philipp Kunkel, Julian F. Wienand, Emily J. Davis, and Monika Schleier-Smith. Programmable interactions and emergent geometry in an array of atom clouds. *Nature*, 600(7890):630–635, December 2021. Publisher: Nature Publishing Group.

[PYK+15] T. P. Purdy, P.-L. Yu, N. S. Kampel, R. W. Peterson, K. Cicak, R. W. Simmonds, and C. A. Regal. Optomechanical raman-ratio thermometry. *Physical Review A*, 92(3), September 2015.

[RMVL22] Christopher X. Ren, Sidhant Misra, Marc Vuffray, and Andrey Y. Lokhov. Learning continuous exponential families beyond gaussian, 2022.

[RWRY11] Pradeep Ravikumar, Martin J. Wainwright, Garvesh Raskutti, and Bin Yu. High-dimensional covariance estimation by minimizing ℓ_1-penalized log-determinant divergence. *Electronic Journal of Statistics*, 5(none):935–980, January 2011. Publisher: Institute of Mathematical Statistics and Bernoulli Society.

[SA25] Matteo Scandi and Álvaro M. Alhambra. Thermalization in open many-body systems and KMS detailed balance, November 2025. arXiv:2505.20064 [quant-ph].

[SCW06] Norbert Schuch, J. Ignacio Cirac, and Michael M. Wolf. Quantum states on harmonic lattices. *Communications in Mathematical Physics*, 267(1):65–92, 2006.

[Ser17] Alessio Serafini. *Quantum continuous variables: a primer of theoretical methods*. CRC press, 2017.

[SNM25] Koki Shiraishi, Masaya Nakagawa, and Takashi Mori. Davies equation without the secular approximation: Reconciling locality with quantum thermodynamics for open quadratic systems, July 2025. arXiv:2507.10080 [quant-ph].

[SWW+23] Alen Senanian, Logan G. Wright, Peter F. Wade, Hannah K. Doyle, and Peter L. McMahon. Programmable large-scale simulation of bosonic transport in optical synthetic frequency lattices. *Nature Physics*, 19(9):1333–1339, September 2023. Publisher: Nature Publishing Group.

[Wai19] Martin J. Wainwright. *High-Dimensional Statistics: A Non-Asymptotic Viewpoint*. Cambridge Series in Statistical and Probabilistic Mathematics. Cambridge University Press, Cambridge, 2019.

[WRG16] Lingxiao Wang, Xiang Ren, and Quanquan Gu. Precision matrix estimation in high dimensional gaussian graphical models with faster rates. In *Artificial Intelligence and Statistics*, pages 177–185. PMLR, 2016.

[WWR10] Wei Wang, Martin J. Wainwright, and Kannan Ramchandran. Information-theoretic bounds on model selection for gaussian markov random fields. In *2010 IEEE International Symposium on Information Theory*, pages 1373–1377, 2010.

[YKB+22] Amir Youssefi, Shingo Kono, Andrea Bancora, Mahdi Chegnizadeh, Jiahe Pan, Tatiana Vovk, and Tobias J. Kippenberg. Topological lattices realized in superconducting circuit optomechanics. *Nature*, 612(7941):666–672, December 2022. Publisher: Nature Publishing Group.

[YL07] Ming Yuan and Yi Lin. Model Selection and Estimation in the Gaussian Graphical Model. *Biometrika*, 94(1):19–35, 2007. Publisher: [Oxford University Press, Biometrika Trust].

[Yua10] Ming Yuan. High Dimensional Inverse Covariance Matrix Estimation via Linear Programming. *Journal of Machine Learning Research*, 11(79):2261–2286, 2010.

[ZZ14] Teng Zhang and Hui Zou. Sparse precision matrix estimation via lasso penalized d-trace loss. *Biometrika*, 101(1):103–120, 2014.

We thank the referees for their positive comments. The final version is heavily restructured to adhere to Nature Communications guidelines. In a final comprehensive consistency check aided by recent large language models, several corrections and clarifications were made, with minor changes of exponents and numerical constants but with no repercussions on the central claims. Most importantly:

- A correction to the account of the known results in the classical case: the reported dependence on the degree and the norm of the precision matrix were incorrect.
- The bookkeping of the analysis trace distance learning algorithm has been corrected, most importantly correcting the final statement on the dependence on $\beta_{\max}$. The previous logic was correct, but the final statement was only valid for $2\beta_{\max} > 1$.
- The bookkeping of the analysis of the Hamiltonian learning algorithm in the polynomial growth regime has been corrected, most importantly correcting the logarithmic dependence on ϵ.
- The bookkeping of the analysis of the graph learning algorithm has been corrected.
- Numerics has been rerun with a seed to ensure reproducibility, and we fixed minor inconsistencies with the algorithm analyzed in the main text.

A document with highlighted changes is also supplied. Since the structure changed considerably, we used GPT 5.6 to prepare a visually useful diff.

We address the remaining points in the following.

Reply to Reviewer 5

1. We still think that a comparison table between this work and [MMB+25]/[BMM+24]/ will be valuable in the final version and should be included. It should compare: setting/task (Hamiltonian / graph / trace-distance learning), measurement model (heterodyne, etc.), assumptions (known graph? bounded degree?), sample complexity scaling (in m, epsilon and other key parameters), computational scaling.

Reply. We have added a comparison table, with the level of detail we feel appropriate for a concise summary.

We also introduced the term *bounded parameters assumptions* for the assumption that all parameters other than the target precision and the number of modes as bounded by constants. Under this condition, the total energy is itself bounded by a constant. We therefore chose not to express our trace-distance learning result in terms of the total energy in the comparison table. Moreover, using the extensive average energy in the parametrization of the sample complexity is not necessarily a good choice. Its use in previous formulations was partly motivated by the simplicity of the resulting presentation. Subsequent results (which we do not discuss in the text as they appeared after our submission) have instead used quantities such as the operator norm of the covariance matrix, see e.g. Theorem 5 in [CMF+26]. More recent work shows that the energy dependence can be avoided altogether [CFG+26]. We have nevertheless retained a discussion of the energy-based reformulation of our result in the main text, with details in the Supplementary Information. For Hamiltonian and graph learning, the table displays only the dependence on the number of modes and on the relevant precision parameter. The complete dependence on the remaining parameters is considerably more involved and would make the table difficult to read: it is stated explicitly in the Supplementary Information.

2. Between Eq(9) and (10) the authors define E_m as the total energy. This is confusing as this is not the Hamiltonian of the system under consideration. It would be more natural here to connect it to particle number as mentioned by the authors in their reply to our earlier question about $\|S\|_\infty$.

Reply. We have changed that sentence to avoid ambiguity. Now we clarify that E_m is the standard harmonic-oscillator energy observable, which is directly related to the total particle-number operator.

3. In (17), relation between N and $\|S\|_\infty$, d_{max} etc. should be stated in loose terms as in (15). Given that there can be implicit relations between these quantities and the size of the system in some cases, it is only fair that it should be stated in the informal theorem statement.

Reply. We agree that the assumptions should be visible directly in the informal theorem statement. By adding the bounded parameters assumptions in the main text, we are able to recall it in the theorem statements, avoiding any misunderstanding for a reader that only looks at the statement. However, we feel that writing the complete dependence in the main text would be lenghty and obscure the message. The details are available in the Supplementary Information.

Kind Regards,

Marco Fanizza, Cambyse Rouzé, Daniel Stilck França

Bibliography

[CFG+26] Senrui Chen, Marco Fanizza, Filippo Girardi, Ludovico Lami, Francesco Anna Mele, Michael Walter, and Freek Witteveen. Optimal tomography of bosonic and fermionic gaussian states. *arXiv preprint arXiv:2607.11847*, 2026.

[CMF+26] Senrui Chen, Francesco Anna Mele, Marco Fanizza, Alfred Li, Zachary Mann, Hsin-Yuan Huang, Yanbei Chen, and John Preskill. Towards sample-optimal learning of bosonic gaussian quantum states. *arXiv preprint arXiv:2603.18136*, 2026.
